# The Efficacy and Acceptability of Non‐Invasive Brain Stimulation Interventions for Obsessive‐Compulsive Disorder Management: A Network Meta‐Analysis Based on 24 Stimulation Methods

**DOI:** 10.1111/acps.13809

**Published:** 2025-03-31

**Authors:** Ping‐Tao Tseng, Chih‐Wei Hsu, Chao‐Ming Hung, Chih‐Sung Liang, Hung‐Yu Wang, Brendon Stubbs, Andre F. Carvalho, Andre R. Brunoni, Kuan‐Pin Su, Yu‐Kang Tu, Yi‐Cheng Wu, Tien‐Yu Chen, Dian‐Jeng Li, Pao‐Yen Lin, Yen‐Wen Chen, Kuo‐Chuan Hung, Jiann‐Jy Chen, Bing‐Syuan Zeng, Cheng‐Ta Li

**Affiliations:** ^1^ Department of Psychology College of Medical and Health Science, Asia University Taichung Taiwan; ^2^ Prospect Clinic for Otorhinolaryngology & Neurology Kaohsiung Taiwan; ^3^ Institute of Biomedical Sciences, National Sun Yat‐sen University Kaohsiung Taiwan; ^4^ Institute of Precision Medicine, National Sun Yat‐sen University Kaohsiung Taiwan; ^5^ Department of Psychiatry Taipei Veterans General Hospital Taipei Taiwan; ^6^ Department of Psychiatry Kaohsiung Chang Gung Memorial Hospital and Chang Gung University College of Medicine Kaohsiung Taiwan; ^7^ Division of General Surgery, Department of Surgery, E‐Da Cancer Hospital I‐Shou University Kaohsiung Taiwan; ^8^ School of Medicine, College of Medicine I‐Shou University Kaohsiung Taiwan; ^9^ Department of Psychiatry, Beitou Branch, Tri‐Service General Hospital; School of Medicine National Defense Medical Center Taipei Taiwan; ^10^ Graduate Institute of Medical Sciences, National Defense Medical Center Taipei Taiwan; ^11^ Kaohsiung Municipal Kai‐Syuan Psychiatric Hospital Kaohsiung City Taiwan; ^12^ Department of Psychological Medicine Institute of Psychiatry, Psychology and Neuroscience, King's College London London UK; ^13^ Centre for Sport Science and University Sports University of Vienna Vienna Austria; ^14^ Innovation in Mental and Physical Health and Clinical Treatment (IMPACT) Strategic Research Centre, School of Medicine, Barwon Health Deakin University Geelong Victoria Australia; ^15^ Service of Interdisciplinary Neuromodulation, National Institute of Biomarkers in Psychiatry, Laboratory of Neurosciences (LIM‐27), Departamento e Instituto de Psiquiatria Faculdade de Medicina da University of Sao Paulo Sao Paulo Brazil; ^16^ Departamento de Ciências Médicas Faculdade de Medicina da University of Sao Paulo Sao Paulo Brazil; ^17^ Department of Psychiatry & Mind‐Body Interface Laboratory (MBI‐Lab) China Medical University Hospital Taichung Taiwan; ^18^ College of Medicine China Medical University Taichung Taiwan; ^19^ An‐Nan Hospital China Medical University Tainan Taiwan; ^20^ Institute of Epidemiology & Preventive Medicine, College of Public Health, National Taiwan University Taipei Taiwan; ^21^ Department of Dentistry National Taiwan University Hospital Taipei Taiwan; ^22^ Department of Sports Medicine Landseed International Hospital Taoyuan Taiwan; ^23^ Department of Psychiatry Tri‐Service General Hospital; School of Medicine, National Defense Medical Center Taipei Taiwan; ^24^ Institute of Brain Science, National Yang Ming Chiao Tung University Taipei Taiwan; ^25^ Department of Addiction Science Kaohsiung Municipal Kai‐Syuan Psychiatric Hospital Kaohsiung Taiwan; ^26^ Kaohsiung Chang Gung Memorial Hospital and Chang Gung University College of Medicine Kaohsiung Taiwan; ^27^ Department of Anesthesiology Chi Mei Medical Center Tainan Taiwan; ^28^ Department of Otorhinolaryngology, E‐Da Cancer Hospital I‐Shou University Kaohsiung Taiwan; ^29^ Department of Internal Medicine, E‐Da Cancer Hospital I‐Shou University Kaohsiung Taiwan; ^30^ Division of Psychiatry, School of Medicine National Yang Ming Chiao Tung University Taipei Taiwan; ^31^ Institute of Brain Science and Brain Research Center, School of Medicine, National Yang Ming Chiao Tung University Taipei Taiwan

**Keywords:** network meta‐analysis, non‐invasive brain stimulation, obsessive‐compulsive disorder, OCD, rTMS, tDCS

## Abstract

**Introduction:**

Despite the high lifetime prevalence and elevated disability rates, treatments for obsessive‐compulsive disorder (OCD) have limited efficacy. Considering the abnormal connectivity in the cortical‐striatal‐thalamic‐cortical loop circuits in OCD, several randomized controlled trials (RCTs) have addressed the efficacy of different non‐invasive brain stimulation (NIBS) modalities for the management of OCD. However, these RCTs yielded inconclusive results.

**Methods:**

This network meta‐analysis (NMA) included RCTs of NIBS interventions, such as transcranial direct current stimulation (tDCS) and various repetitive transcranial magnetic stimulation (rTMS), in OCD patients. The primary outcomes were changes in the overall severity of OCD and acceptability (i.e., dropout rates).

**Results:**

This NMA of 34 eligible RCTs (1089 participants) and 24 different NIBS interventions revealed that three NIBS interventions significantly improved overall OCD severity compared with sham controls, which were high‐frequency rTMS over the dorsolateral prefrontal cortex (DLPFC) [mean difference (MD) = −10.81, 95% confidence intervals (95% CIs) = −20.80 to −0.82], high‐frequency deep TMS over the dorsal medial prefrontal cortex/anterior cingulate cortex (dmPFC/ACC) (MD = −9.74, 95% CIs = −16.42 to −3.06), and low‐frequency rTMS over the right DLPFC (MD = −4.70, 95% CIs = −8.84 to −0.57).

**Conclusions:**

This study highlighted that excitatory stimulation over the dmPFC/ACC and bilateral DLPFC, or inhibitory stimulation over the right DLPFC, was associated with significant improvements in overall OCD severity. Further large‐scale RCTs with longer follow‐up periods are needed to investigate the true impact of NIBS‐based intervention to manage OCD.

**Trial Registration:**PROSPERO: CRD42023394953

**Abbreviations**
95% CIs: 95% confidence intervals
ACC: anterior cingulate cortex
alpha‐TMS‐F3+F4: alpha EEG guided‐TMS over F3+F4
a‐tDCS‐Fp3+c‐tDCS‐F8: anode tDCS over Fp3 plus cathode tDCS over F8
a‐tDCS‐Fz+c‐tDCS‐extracephalic: anode tDCS over Fz plus cathode tDCS over extracephalic region
CBT: cognitive behavioral therapy
CGI‐S: clinical global impression scale‐severity
cTBS: continuous theta burst stimulation
cTBS‐F3+F4: cTBS over F3+F4
cTBS‐Fp1: cTBS over Fp1
cTBS‐Fp2: cTBS over Fp2
c‐tDCS‐Fp1+a‐tDCS‐O2: cathode tDCS over Fp1 plus anode tDCS over O2
c‐tDCS‐Fp2+a‐tDCS‐F5: cathode tDCS over Fp2 plus anode tDCS over F5
c‐tDCS‐Fz+a‐tDCS‐extracephalic: cathode tDCS over Fz plus anode tDCS over extracephalic region
DLPFC: dorsolateral prefrontal cortex
dmPFC: dorsal medial prefrontal cortex
DSM: Diagnostic and Statistical Manual of Mental Disorders
dTMS: deep TMS
EEG: electroencephalography
Hf‐dTMS‐Fp1Fp2: high‐frequency dTMS over Fp1Fp2
Hf‐rTMS‐F3+F4: high‐frequency rTMS over F3+F4
Hf‐rTMS‐F3: high‐frequency rTMS over F3
Hf‐rTMS‐F4: high‐frequency rTMS over F4
ICD: International Classification of Diseases
Lf‐dTMS‐Fp1Fp2: low‐frequency dTMS over Fp1Fp2
Lf‐rTMS‐F3: low‐frequency rTMS over F3
Lf‐rTMS‐F4: low‐frequency rTMS over F4
Lf‐rTMS‐F5+F6: low‐frequency rTMS over F5+F6
Lf‐rTMS‐Fp1: low‐frequency rTMS over Fp1
Lf‐rTMS‐Fp2: low‐frequency rTMS over Fp2
Lf‐rTMS‐Fz: low‐frequency rTMS over Fz
MD: mean difference
neuronavigated Lf‐rTMS‐F5+F6: neuronavigated low‐frequency rTMS over F5+F6
neuronavigated Lf‐rTMS‐F6: neuronavigated low‐frequency rTMS over F6
NIBS: non‐invasive brain stimulation
NMA: network meta‐analysis
OCD: obsessive‐compulsive disorder
OFC: orbitofrontal cortex
OR: odds ratio
prLf‐rTMS‐F3: priming and low‐frequency rTMS over F3
RCT: randomized controlled trial
rTMS: repetitive TMS
Sham: sham control
SMD: standardized mean difference
SUCRA: surface under the cumulative ranking curve
tDCS: transcranial direct current stimulation
TMS: transcranial magnetic stimulation
YBOCS: Yale‐Brown obsessive compulsive scale

**Summary**

- What is already known on this topic
  ○ Obsessive‐compulsive disorder (OCD) is a severe mental illness associated with high prevalence and morbidity (lifetime prevalence 2.5%–3.0%) with only partial response to the current pharmacologic therapy.
  ○ Although the treatment of OCD remains a clinical challenge, emerging modalities for non‐invasive brain stimulation appear promising.
  ○ However, the previous randomized controlled trials and network meta‐analyses (NMAs) provided controversial results.
- What this study adds
  ○ High‐frequency repetitive transcranial magnetic stimulation (rTMS) over the bilateral dorsolateral prefrontal cortex (DLPFC), high‐frequency deep transcranial magnetic stimulation (dTMS) over the bilateral dorsal medial prefrontal cortex (dmPFC)/anterior cingulate cortex (ACC), and low‐frequency rTMS over the right DLPFC were associated with a significant improvement in the overall severity of OCD.
  ○ In addition, low‐frequency rTMS over the right DLPFC was associated with the greatest improvement in symptoms of depression and anxiety.
  ○ All investigated NIBS modalities were associated with similar acceptability relative to the sham control groups.
- How this study might affect research, practice or policy
  ○ Our study provides updated evidence that excitatory stimulation of the dmPFC/ACC or DLPFC significantly improves overall OCD severity. Inhibitory stimulation over the right DLPFC not only significantly improves overall OCD severity, but also symptoms of depression and anxiety.
- Significant Outcomes
  ○ Excitatory stimulation of the dmPFC/ACC or DLPFC significantly improves overall OCD severity.
  ○ Inhibitory stimulation over the right DLPFC not only significantly improves overall OCD severity but also symptoms of depression and anxiety.
  ○ None of the investigated NIBS modalities were associated with significantly different acceptability relative to the sham control groups.
- Limitations
  ○ Heterogeneity of the participants (e.g., comorbidities, concomitant psychotropic medication, baseline OCD severity, timing of NIBS intervention, definition of response rate, and follow‐up duration).
  ○ The blindness of the RCTs may not have been completely guaranteed because of the limitations of the commercial instruments employed.
  ○ The relatively short treatment duration and overall study duration might limit the observation of treatment efficacy progression, because some patients undergoing neurostimulation would exhibit a delayed response phenomenon.

## 1 Introduction

Obsessive‐compulsive disorder (OCD) is a severe mental illness associated with high prevalence and morbidity (lifetime prevalence 2.5%–3.0%) [1] and is associated with significant impairments in quality of life. Currently, the first‐line treatment strategies for OCD are cognitive–behavioral therapy with/without selective serotonin reuptake inhibitors, which usually have unsatisfactory response rates (i.e., less than 50% response rate) [2].

Although the treatment of OCD remains a clinical challenge, emerging modalities for non‐invasive brain stimulation (NIBS), such as transcranial direct current stimulation (tDCS) and repetitive transcranial magnetic stimulation (rTMS), appear promising. These techniques can induce changes in neuronal activity according to the stimulation parameters. For example, tDCS can lead to regional neuronal membrane polarization and either an increase or decrease in cortical excitability [3]. Similarly, rTMS relies on electromagnetic fields to produce micro‐electric current in the target brain, thereby promoting excitatory or inhibitory effects on brain function [4]. Several large‐scale studies have addressed the application of different NIBS modalities in different neuropsychiatric diseases. Of these different devices using rTMS protocols for OCD, some of the devices applying rTMS have received FDA approval. Most recently, bilateral high‐frequency rTMS stimulation of the dorsomedial prefrontal cortex (dmPFC) received FDA approval in August 2020 [5]. Several randomized controlled trials (RCTs) have also addressed the efficacy of bilateral high‐frequency rTMS of dmPFC stimulation in the management of OCD [6, 7]. However, in addition to the aforementioned FDA approved device, some novel NIBSs were also recommended as options for OCD treatment, such as deep transcranial magnetic stimulation (dTMS) [8]. The application of dTMS, via its deep penetration property, could stimulate both dorsal mPFC and ACC regions [7], which had been found to be dysfunctional in OCD patients [9, 10]. Therefore, it might theoretically provide a superior efficacy compared to those devices stimulating one site only. There is a lack of evidence exploring the comparative benefits regarding those new devices and the aforementioned FDA approved devices.

Several network meta‐analyses (NMAs) have been published, but the results are controversial due to several methodological issues [11, 12, 13]. First, those previous NMAs mainly focused on rTMS modalities but did not include all NIBS modalities [11, 12], which might limit the generation of inferences to guide clinical practice and research. Second, some NMAs may have oversimplified the classification of various NIBS protocols. For example, by treating different NIBS modalities as the same experimental arm [13], excluding RCTs with multiple target regions [12], or focusing on only one brain region in a single treatment arm [11]. Oversimplified classifications may increase the inconsistency in results and limit the clinical utility of NIBS. Third, some meta‐analyses may have employed looser exclusion criteria for included studies. For example, combining included studies with quasi‐randomization designs [14, 15], or including potential replicate samples that may have come from the same source [16, 17, 18]. This can weaken evidence‐based grading recommendations.

A comprehensive NMA can help resolve such controversial findings resulting from these methodological issues; by following a more rigorous design and including all types of NIBS from all available unique RCTs, it can provide a more consistent portrait of the efficacy and acceptability of different NIBS approaches for the management of OCD. Furthermore, it clarifies the relative merits of multiple interventions, a key feature that the aforementioned pairwise meta‐analyses were unable to address.

### 1.1 Aims of the Study

We conducted a comprehensive NMA to compare the efficacy and acceptability of various NIBS methods and protocols for the management of OCD with the hope of providing clearer clinical and research guidance in this emerging field of scientific inquiry.

## 2 Methods

### 2.1 General Study Guidelines

This NMA was conducted in accordance with the extended 2020 version of the Preferred Reporting Items for Systematic Reviews and Meta‐Analyses Guidelines (PRISMA) (Table S1–S10). The current study was approved by the Institutional Review Board of the Tri‐Service General Hospital, National Defense Medical Center (TSGHIRB no. B‐109–29) and registered a priori in PROSPERO (CRD42023394953).

### 2.2 NMA Objectives

This study compared the effects of different NIBS methods and protocols on the severity of OCD symptoms in patients with OCD. The PICO (population, intervention, comparison, outcome) setting of the current NMA was as follows: (1) *Population*: Adult patients with an established diagnosis of OCD, either according to the DSM or ICD diagnostic systems; (2) *interventions*: Treatment with a course of NIBS treatment (at least 1‐week treatment duration), including dTMS, rTMS, or tDCS; (3) *comparison*: The results of the active interventions were compared with those of the sham control or active control groups; and (4) *outcome*: Change in the severity of OCD. Regarding the version of the *Diagnostic and Statistical Manual of Mental Disorders* (DSM) and the *International Classification of Diseases* (ICD), because of the presumed wide variety of publication years, we did not set any limitation to a specific version of the DSM or ICD. Only RCTs investigating changes in the severity of OCD symptoms after NIBS as their primary or secondary outcome were deemed eligible for inclusion in the current NMA.

### 2.3 Search Strategies and Screening Process

We searched the ClinicalKey, Cochrane CENTRAL, EMBASE, ProQuest, PubMed, ScienceDirect, and Web of Science databases for RCTs from inception through January 27, 2023 (for the detailed search strategy, please see Table S2). To identify reports from the grey literature and unpublished studies, we searched ClinicalTrials.gov. Language restrictions were not applied in the literature search. The search strategy was further augmented by the manual search of the reference lists of eligible articles as well as previous review articles and meta‐analyses/network meta‐analyses on this topic. The overall screening and selection strategy included two stages, which were performed by independent reviewers: The first stage comprised title and abstract screening, and the second was full‐text screening and selection.

### 2.4 Eligibility Criteria

To comply with the transitivity assumptions and reduce heterogeneity across the included studies, we applied the following stringent inclusion criteria: (1) RCTs; (2) application of NIBS interventions; (3) participants with an established diagnosis of OCD; and (4) studies comparing the efficacy of different NIBS strategies to relieve participants' OCD‐related symptoms.

Studies were excluded if they (1) were not RCTs, (2) did not assess the severity of OCD symptoms using validated methods, (3) were not related to NIBS, (4) did not recruit participants with OCD, or (5) applied less than 1 week of NIBS stimulation. In cases of duplicate reporting (i.e., different articles based on the same sample), we included the article with the largest sample size.

### 2.5 Data Extraction

Two authors independently screened the references, extracted relevant information from the articles, and evaluated the risk of bias of the included studies. Disagreements were resolved through consensus or discussion with a third investigator. Whenever data were lacking in the original manuscript, the corresponding authors or co‐authors were contacted to obtain the full original data on at least two occasions over a one‐week period. We followed a flowchart in accordance with the procedures of other NMAs. The nomenclature of brain mapping was based on 10–20 electroencephalography mapping (i.e., F3 for the left DLPFC, Fp1 for the left mPFC, and F4 for the right DLPFC). The nomenclature and classification of the treatment arms were named according to our previous five NMAs on NIBS approaches for other conditions [19, 20, 21, 22, 23, 24, 25].

### 2.6 Outcomes

The co‐primary outcomes were (1) changes in the overall OCD severity scores after NIBS management relative to the sham control and (2) acceptability (i.e., dropout rate). The dropout rate was defined when patients left the study for any reason before the study's completion. The choice of co‐primary outcomes was based on widely accepted rationales from other NMAs investigating NIBS [22, 23, 24]. Secondary outcomes included improvements in clinical impression (i.e., changes in clinical global impression‐severity [CGI‐S]), depression, and anxiety symptoms. The response rate was assessed by calculating the number of participants with a sufficient response to the NIBS modalities. The definition of response varied among RCTs. We considered the final outcomes at the last visit available in each eligible RCT to account for the possible delayed beneficial effects of NIBS on OCD symptoms.

### 2.7 Risk of Bias Assessment

Two authors independently evaluated the risk of bias (inter‐rater reliability = 0.87) for each domain (i.e., selection bias, performance bias, detection bias, attrition bias, reporting bias, and other biases) based on the Cochrane risk of bias tool. Specifically, the item “other bias” was assessed for any potential bias not addressed by the existing risk of bias items [26].

### 2.8 Statistical Analysis

The NMA was performed using STATA (version 16.0; StataCorp, College Station, TX, USA). We employed the *mvmeta* command in STATA [27]. For continuous variables, we estimated the standardized mean differences (SMDs) with 95% confidence intervals (95% CIs). If all recruited RCTs selected the same rating scales for an outcome, we used mean differences (MD) because they might provide more information to clinicians than SMD. For categorical variables (i.e., dropout rate and adverse event rates), we estimated the odds ratios (OR) and 95% CIs. As required by the *mvmeta* command in STATA, we applied a 0.5 zero‐cell correction during the meta‐analysis if a study had zero events in either the intervention or control arm. However, if a study had zeros in all the intervention and control arms, we did not perform a correction procedure because of the increased risk of bias; instead, we excluded these studies from our analysis [28, 29]. All pairwise meta‐analyses and NMA procedures were conducted using random effects and frequentist models, respectively. Heterogeneity among the included studies was evaluated using the tau value, which is the estimated standard deviation of the effects across the studies. All comparisons were performed using a two‐tailed test, and *p* values < 0.05 were considered statistically significant.

This study used a mixed comparison with generalized linear mixed models to analyze direct and indirect comparisons in the NMA [30]. Specifically, indirect comparisons were conducted using transitivity, meaning that differences between treatments A and B could be calculated from their comparisons with a third treatment, C. To compare multiple treatment arms, this study combined the direct and indirect evidence of the included studies [31]. The restricted maximum likelihood method was used to evaluate the variance between the studies [32]. To provide additional clinical applications, this study calculated the relative ranking probabilities of the effects of all treatments on the target outcomes. In brief, the surface under the cumulative ranking curve (SUCRA) indicated the percentage of the mean rank of each intervention relative to an imaginary intervention that was optimal without uncertainty [33]. This study used comparison‐adjusted funnel plots and Egger's regression to evaluate potentially small study effects and publication bias. In addition, this study evaluated potential inconsistencies using a loop‐specific approach, node splitting, and a design‐by‐treatment model.

Finally, in line with the rationale of another NMA [23, 24], this study assessed the effectiveness of different sham interventions (i.e., changes in overall OCD severity) to justify our assumption of transitivity. Specifically, we computed the changes in overall OCD severity relative to tDCS sham therapy and TMS sham therapy using Comprehensive Meta‐Analysis software (version 3; Biostat, Englewood, NJ, USA). Furthermore, this study conducted subgroup analyses focusing on RCTs with sham controls, but not active controls.

## 3 Results

**FIGURE 1:**
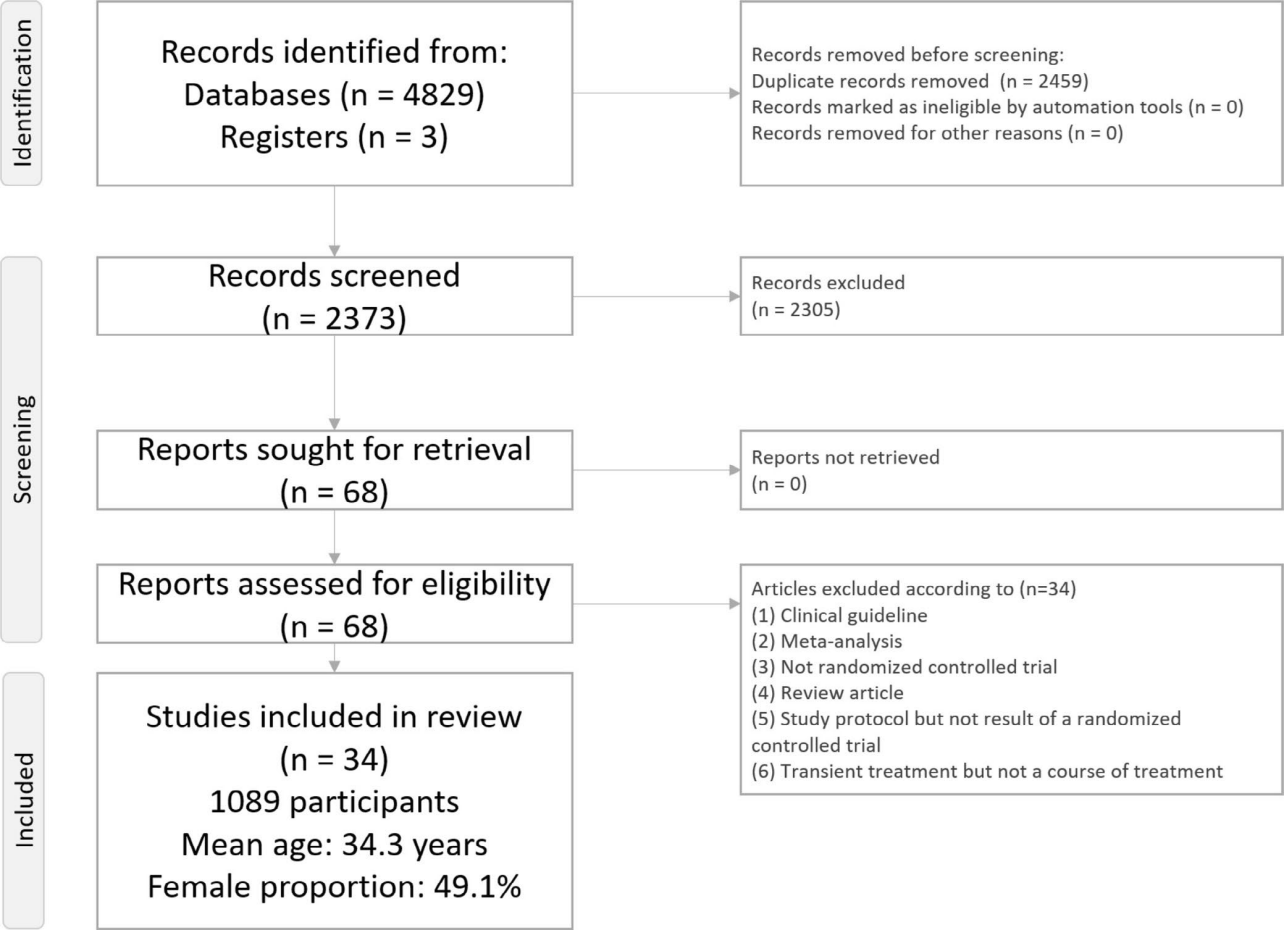
Network meta‐analysis flowchart.

**FIGURE 2:**
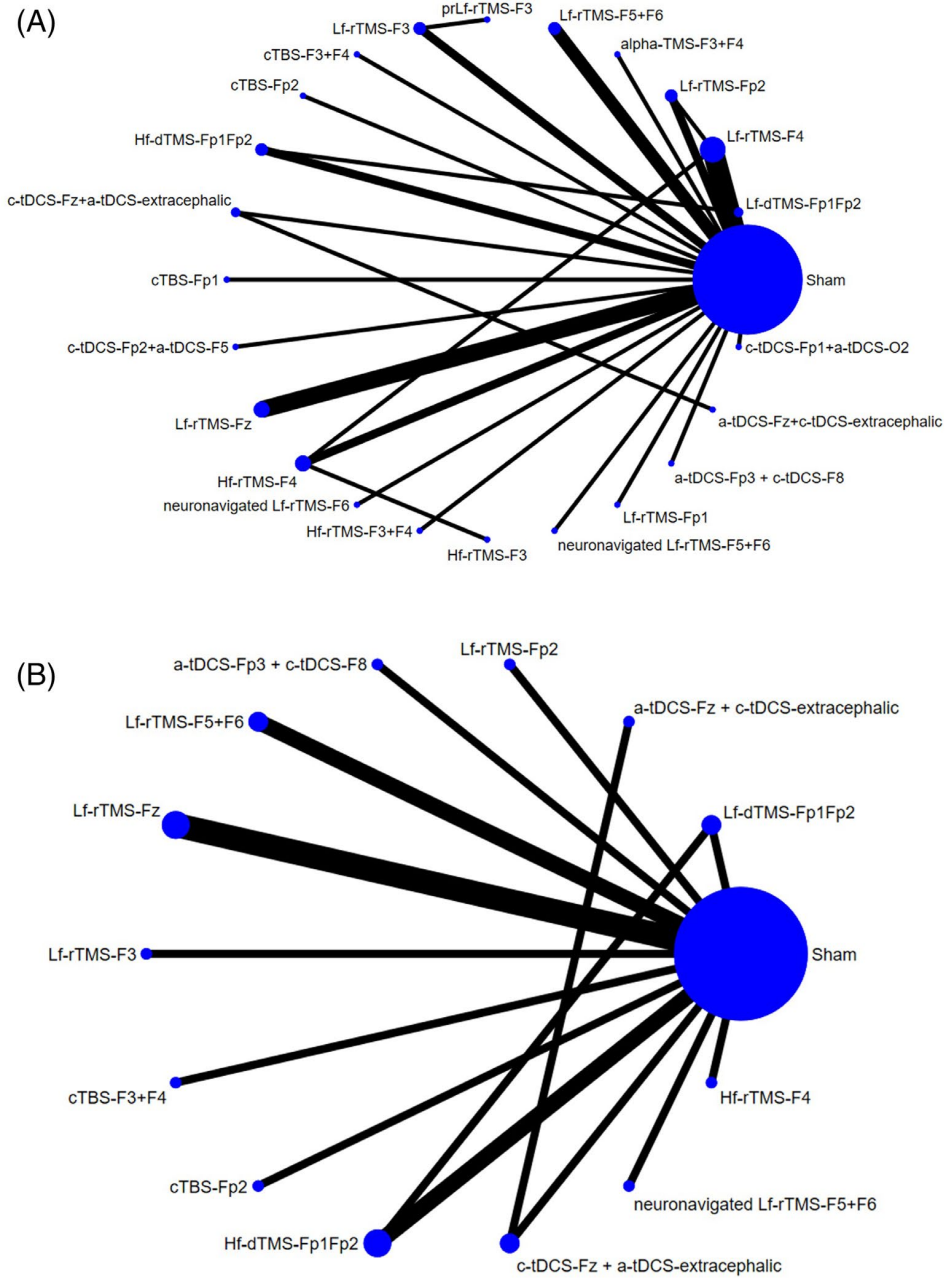
Network structure of (A) changes in overall severity of OCD and (B) acceptability with respect to drop‐out rate. Lines between nodes represent direct comparisons between trials, and circle size is proportional to the size of the population that received each treatment. Line thickness is proportional to the number of trials connected to the network.

After initial screening of the records from the literature search, 68 articles were retrieved for a full‐text review (Figure 1). Thirty‐four articles were excluded for various reasons (Figure 1 and Table S3). Finally, 34 RCTs published between 2004 and 2022 were included in the current meta‐analysis (Table S4). Figure 2A,B illustrates the network structure of the treatment arms.

### 3.1 Characteristics of the Included Studies

The baseline characteristics of the study participants are listed in Table S4. A total of 1089 participants with OCD (mean age 34.3; range: 27.1–42.9 years; mean proportion 49.1%, range: 15.0%–81.7%) were included. The mean treatment duration and mean overall study duration (i.e., treatment + follow‐up duration) were 3.5 (range: 1–8) weeks and 8.8 (range: 2–26) weeks, respectively. None of the included RCTs prohibited concurrent pharmacological treatments or psychotherapy during the study period. Therefore, the NIBS, either TMS or tDCS, were used as an adjunctive treatment in their OCD management strategy. The diagnosis of OCD was confirmed based on the *Diagnostic and Statistical Manual of Mental Disorders* (DSM‐IV, DSM‐IV‐TR, and DSM‐5) *or International Classification of Diseases* criteria. The response rate was defined as a 25%, 30%, 35%, or 40% reduction in the Yale‐Brown Obsessive‐Compulsive Scale (YBOCS) score.

### 3.2 Co‐Primary Outcomes: Changes in OCD Overall Severity

**FIGURE 3:**
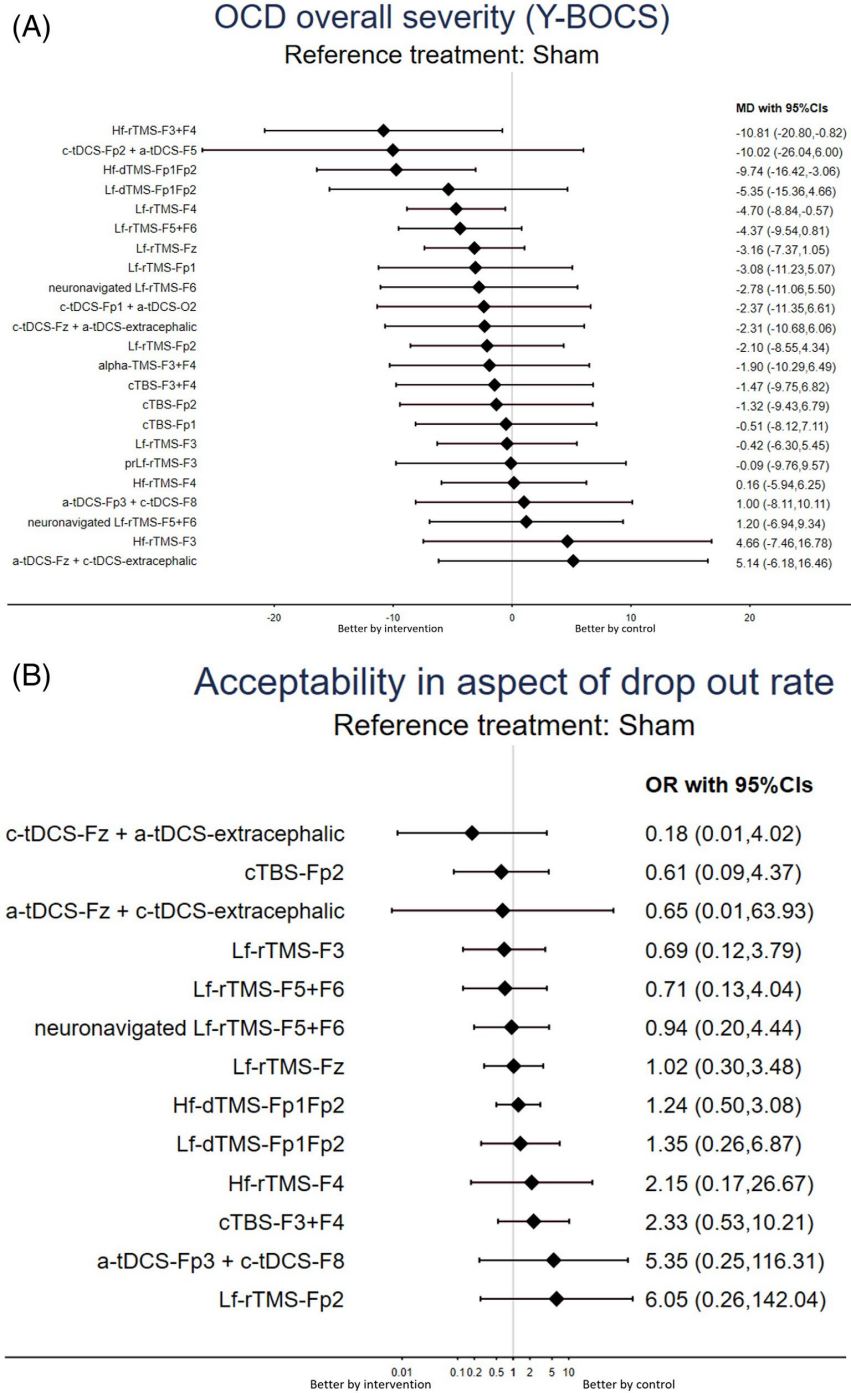
Forest plot of (A) changes in OCD overall severity and (B) acceptability in aspect of drop‐out rate. (A) When the effect size is < 0, the specified intervention is associated with a greater improvement in OCD overall severity compared with the sham controls. (B) When the effect size is < 1, the specified intervention is associated with a better acceptability (i.e., lower drop‐out rate) compared with the sham controls. Abbreviations: 95% CIs: 95% confidence intervals; ACC, anterior cingulate cortex; alpha‐TMS‐F3+F4, alpha EEG guided‐TMS over F3+F4; a‐tDCS‐Fp3+c‐tDCS‐F8, anode tDCS over Fp3 plus cathode tDCS over F8; a‐tDCS‐Fz+c‐tDCS‐extracephalic, anode tDCS over Fz plus cathode tDCS over extracephalic region; CBT, cognitive behavioral therapy; CGI‐S, clinical global impression scale‐severity; cTBS, continuous theta burst stimulation; cTBS‐F3+F4, cTBS over F3+F4; cTBS‐Fp1, cTBS over Fp1; cTBS‐Fp2, cTBS over Fp2; c‐tDCS‐Fp1+a‐tDCS‐O2, cathode tDCS over Fp1 plus anode tDCS over O2; c‐tDCS‐Fp2+a‐tDCS‐F5, cathode tDCS over Fp2 plus anode tDCS over F5; c‐tDCS‐Fz+a‐tDCS‐extracephalic, cathode tDCS over Fz plus anode tDCS over extracephalic region; DLPFC, dorsolateral prefrontal cortex; dmPFC, dorsal medial prefrontal cortex; DSM, Diagnostic and Statistical Manual of Mental Disorders; dTMS, deep TMS; EEG, electroencephalography; Hf‐dTMS‐Fp1Fp2, high‐frequency dTMS over Fp1Fp2; Hf‐rTMS‐F3, high‐frequency rTMS over F3; Hf‐rTMS‐F3+F4, high‐frequency rTMS over F3+F4; Hf‐rTMS‐F4, high‐frequency rTMS over F4; ICD, International Classification of Diseases; Lf‐dTMS‐Fp1Fp2, low‐frequency dTMS over Fp1Fp2; Lf‐rTMS‐F3, low‐frequency rTMS over F3; Lf‐rTMS‐F4, low‐frequency rTMS over F4; Lf‐rTMS‐F5+F6, low‐frequency rTMS over F5+F6; Lf‐rTMS‐Fp1, low‐frequency rTMS over Fp1; Lf‐rTMS‐Fp2, low‐frequency rTMS over Fp2; Lf‐rTMS‐Fz, low‐frequency rTMS over Fz; MD, mean difference; neuronavigated Lf‐rTMS‐F5+F6, neuronavigated low‐frequency rTMS over F5+F6; neuronavigated Lf‐rTMS‐F6, neuronavigated low‐frequency rTMS over F6; NIBS, non‐invasive brain stimulation; NMA, network meta‐analysis; OCD, obsessive‐compulsive disorder; OFC, orbitofrontal cortex; OR, odds ratio; prLf‐rTMS‐F3, priming and low‐frequency rTMS over F3; RCT, randomized controlled trial; rTMS, repetitive TMS; Sham, sham control; SMD, standardized mean difference; SUCRA, surface under the cumulative ranking curve; tDCS, transcranial direct current stimulation; TMS, transcranial magnetic stimulation; YBOCS, Yale‐Brown obsessive compulsive scale.

High‐frequency rTMS over F3+F4 (Hf‐rTMS‐F3+F4), high‐frequency dTMS over Fp1Fp2 (Hf‐dTMS‐Fp1Fp2), and low‐frequency rTMS over F4 (Lf‐rTMS‐F4) were associated with significantly better improvement in overall OCD severity than the sham controls were (Table 1A and Figure 3A). According to the SUCRA, Hf‐rTMS‐F3+F4 was associated with the greatest improvement, followed by Hf‐dTMS‐Fp1Fp2 (Table S5A).

**TABLE 1A:** League table of the overall OCD severity (YBOCS).

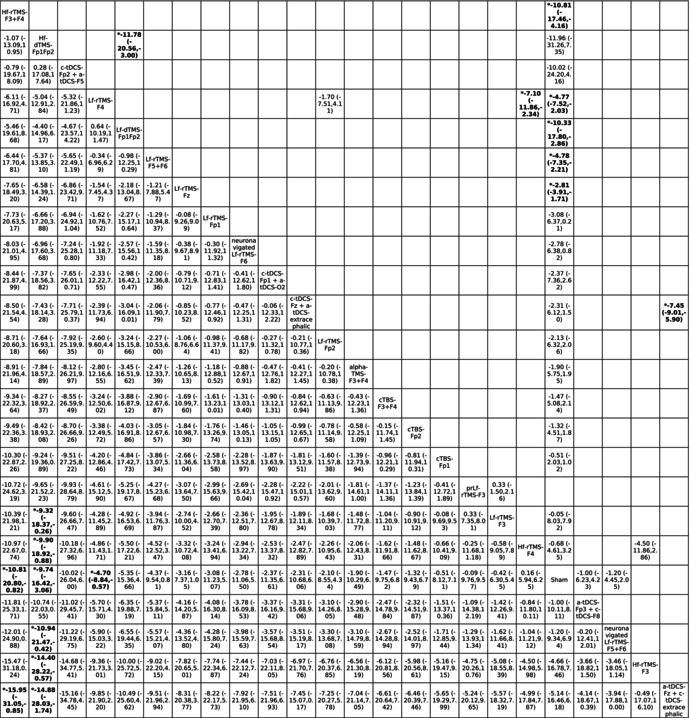

*Note*: Pairwise (upper‐right portion) and network (lower‐left portion) meta‐analysis results are presented as estimate effect sizes for the outcome of improvement of overall OCD severity. Interventions are reported in order of mean ranking of severity improvement, and outcomes are expressed as mean difference (MD) (95% confidence intervals). For the pairwise meta‐analyses, MD of less than 0 indicate that the treatment specified in the row got more improvement than that specified in the column. For the network meta‐analysis (NMA), MD of less than 0 indicate that the treatment specified in the column got more improvement than that specified in the row. Bold results marked with * indicate statistical significance.

The assumption of transitivity (i.e., to assume that there was no significant inconsistency within the Node “sham controls”) was evaluated according to the rationale of a previous NMA [23, 24]. We noticed both TMS sham controls and tDCS sham controls were associated with significant improvement in overall OCD severity. Furthermore, no significant differences were detected between the TMS and tDCS sham control groups (*p* = 0.305; Figure S2A).

The subgroup analysis focusing on RCTs with sham‐controlled arm revealed similar findings: Hf‐rTMS‐F3+F4, Hf‐dTMS‐Fp1Fp2, and Lf‐rTMS‐F4 were associated with significantly greater alleviation of OCD overall severity compared with the sham controls (Table S6A; Figures S1A– and S2B). According to the SUCRA, Hf‐rTMS‐F3+F4 was associated with the greatest improvement, followed by Hf‐dTMS‐Fp1Fp2 (Table S5B).

### 3.3 Co‐Primary Outcome: Acceptability With Respect to Drop‐Out Rate

None of the investigated NIBS modalities were associated with significantly different acceptability relative to the sham control groups (Tables 1A and 1B; Table S5C; Figures 2B and 3B).

**TABLE 1B:** League table of the acceptability in aspects of drop‐out rate.

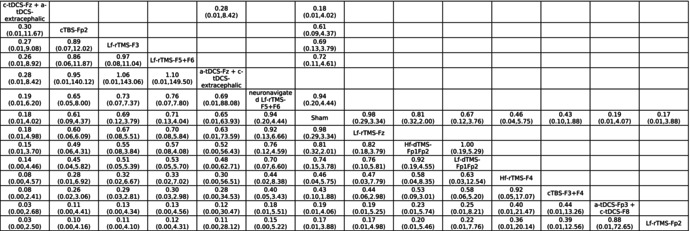

*Note*: Pairwise (upper‐right portion) and network (lower‐left portion) meta‐analysis results are presented as estimate effect sizes for the outcome of acceptability in aspect of drop‐out rate. Interventions are reported in order of mean ranking of acceptability, and outcomes are expressed as odds ratio (OR) (95% confidence intervals). For the pairwise meta‐analyses, OR of less than 1 indicate that the treatment specified in the row got more acceptability than that specified in the column. For the network meta‐analysis (NMA), OR of less than 1 indicate that the treatment specified in the column got more acceptability than that specified in the row. Bold results marked with * indicate statistical significance.

Abbreviations: 95% CIs: 95% confidence intervals; ACC, anterior cingulate cortex; alpha‐TMS‐F3+F4, alpha EEG guided‐TMS over F3+F4; a‐tDCS‐Fp3+c‐tDCS‐F8, anode tDCS over Fp3 plus cathode tDCS over F8; a‐tDCS‐Fz+c‐tDCS‐extracephalic, anode tDCS over Fz plus cathode tDCS over extracephalic region; CBT, cognitive behavioral therapy; CGI‐S, clinical global impression scale‐severity; cTBS, continuous theta burst stimulation; cTBS‐F3+F4, cTBS over F3+F4; cTBS‐Fp1, cTBS over Fp1; cTBS‐Fp2, cTBS over Fp2; c‐tDCS‐Fp1+a‐tDCS‐O2, cathode tDCS over Fp1 plus anode tDCS over O2; c‐tDCS‐Fp2+a‐tDCS‐F5, cathode tDCS over Fp2 plus anode tDCS over F5; c‐tDCS‐Fz+a‐tDCS‐extracephalic, cathode tDCS over Fz plus anode tDCS over extracephalic region; DLPFC, dorsolateral prefrontal cortex; dmPFC, dorsal medial prefrontal cortex; DSM, diagnostic and statistical manual of mental disorders; dTMS, deep TMS; EEG, electroencephalography; Hf‐dTMS‐Fp1Fp2, high‐frequency dTMS over Fp1Fp2; Hf‐rTMS‐F3, high‐frequency rTMS over F3; Hf‐rTMS‐F3+F4, high‐frequency rTMS over F3+F4; Hf‐rTMS‐F4, high‐frequency rTMS over F4; ICD, International Classification of Diseases; Lf‐dTMS‐Fp1Fp2, low‐frequency dTMS over Fp1Fp2; Lf‐rTMS‐F3, low‐frequency rTMS over F3; Lf‐rTMS‐F4, low‐frequency rTMS over F4; Lf‐rTMS‐F5+F6, low‐frequency rTMS over F5+F6; Lf‐rTMS‐Fp1, low‐frequency rTMS over Fp1; Lf‐rTMS‐Fp2, low‐frequency rTMS over Fp2; Lf‐rTMS‐Fz, low‐frequency rTMS over Fz; MD, mean difference; neuronavigated Lf‐rTMS‐F5+F6, neuronavigated low‐frequency rTMS over F5+F6; neuronavigated Lf‐rTMS‐F6, neuronavigated low frequency rTMS over F6; NIBS, non‐invasive brain stimulation; NMA, network meta‐analysis; OCD, obsessive‐compulsive disorder; OFC, orbitofrontal cortex; OR, odds ratio; prLf‐rTMS‐F3, priming and low frequency rTMS over F3; RCT, randomized controlled trial; rTMS, repetitive TMS; Sham, sham control; SMD, standardized mean difference; SUCRA, surface under the cumulative ranking curve; tDCS, transcranial direct current stimulation; TMS, transcranial magnetic stimulation; YBOCS, Yale‐Brown obsessive compulsive scale.

### 3.4 Secondary Outcome: Changes in Clinical Impression (CGI‐S)

For this secondary outcome, all the RCTs used the CGI‐S to rate the clinical impression. Therefore, the effect size was calculated using the MD and its 95% CIs. The Hf‐rTMS‐F3+F4, Lf‐rTMS‐F4, and alpha electroencephalography guided‐TMS over bilateral DLPFC (alpha‐TMS‐F3+F4) were associated with significantly greater improvement in clinical impression compared with the sham controls (Table S6B; Figures S1–S4B and S2C). According to SUCRA, Hf‐rTMS‐F3+F4 was associated with the greatest improvement, followed by Lf‐rTMS‐F4 (Table S5D).

### 3.5 Secondary Outcome: Changes in Depression

Only the Lf‐rTMS‐F4 was associated with significantly greater improvement in depression than the sham control groups did (Table S6C; Figures S1–S4C and S2D). According to SUCRA, Lf‐rTMS‐F4 was associated with the greatest improvement among all the investigated NIBS treatments (Table S5E).

### 3.6 Secondary Outcome: Changes in Anxiety Symptoms

Only the Lf‐rTMS‐F4 was associated with significantly better alleviation of anxiety symptoms than the sham control (Table S6D; Figures S1–S4D and S2E). According to SUCRA, Lf‐rTMS‐F4 was associated with the greatest alleviation among all the investigated NIBS modalities (Table S5F).

### 3.7 Secondary Outcome: Response Rate

The result of this secondary outcome of the current NMA revealed that the low‐frequency rTMS over Fz (Lf‐rTMS‐Fz), Hf‐rTMS‐F3+F4, alpha‐TMS‐F3+F4, low‐frequency rTMS over Fp2 (Lf‐rTMS‐Fp2), neuronavigated low‐frequency rTMS over F6 (neuronavigated Lf‐rTMS‐F6), Hf‐dTMS‐Fp1Fp2, Lf‐rTMS‐F4, and low‐frequency rTMS over F5 and F6 (Lf‐rTMS‐F5+F6) were associated with significantly greater response rates than the sham controls (Table S6E; Figures S1–S4E and S2F). According to SUCRA, Lf‐rTMS‐Fz was associated with the highest response rate, followed by Hf‐rTMS‐F3+F4, among all investigated NIBS modalities (Table S5G).

### 3.8 Risk of Bias, Publication Bias, Inconsistency, and Heterogeneity

Of the included studies, 88.6% (211/238 items), 10.1% (24/238 items), and 1.3% (3/238 items) had an overall low, unclear, and high risk of bias, respectively. The unclear reporting of allocation concealment in the studies contributed to the risk of bias (Figures S3A and S3B). Funnel plots of publication bias across the included studies (Figure S4A–L) revealed general symmetry. No significant publication bias was detected among the articles included in the NMA using Egger's test. The NMA did not demonstrate inconsistencies in terms of either local inconsistency (assessed using the loop‐specific approach and node splitting) or global inconsistency (determined using the design‐by‐treatment method) (Tables S7 and S8), except for the overall OCD severity of the global inconsistency using the design‐by‐treatment method (*p* = 0.006). No significant heterogeneity was detected among most comparisons based on tau values (Table S9). The grading of the recommendations, assessment, development, and evaluation revealed that the quality of evidence in the NMA ranged from low to medium (Table S10).

## 4 Discussion

This study demonstrated that, compared with sham controls, excitatory stimulation over specific areas in the bilateral prefrontal cortex (i.e., Hf‐dTMS‐Fp1Fp2 and Hf‐rTMS‐F3+F4) or inhibitory stimulation over the right DLPFC (i.e., Lf‐rTMS‐F4) was associated with a significant improvement in the overall severity of OCD. In contrast, inhibitory stimulation over the right DLPFC (Lf‐rTMS‐F4) was associated with the greatest improvement in mood‐related components (depression and anxiety symptoms). Finally, all investigated NIBS modalities were associated with similar acceptability as that of the sham control groups.

### 4.1 The Role of Excitatory Stimulations Over the Bilateral Prefrontal Cortex and Associated Regions

In the current NMA, both rTMS (i.e., Hf‐rTMS‐F3+F4) and dTMS (i.e., Hf‐dTMS‐Fp1Fp2) devices were associated with significant improvement in OCD symptoms compared with controls. The efficacy of dTMS devices were similar with rTMS one. To be specific, the experiment arm “Hf‐rTMS‐F3+F4” indicated “20‐Hz rTMS over left DLPFC + right DLPFC”. The experiment arm “Hf‐dTMS‐Fp1Fp2” represented “20‐Hz dTMS over bilateral dmPFC and ACC with/without concomitant cognitive behavioral therapy (CBT) or pharmacotherapy”. These additional aforementioned findings were different from the previous NMAs, which only revealed the superiority of LF‐rTMS over the DLPFC among the investigated rTMS modalities [11, 12]. This discrepancy could be the result of the following issues. First, in contrast to previous NMAs that merged different NIBS modalities into one experimental arm [13], excluded RCTs involving multiple targeted regions [12], or focused only on one brain region in one experimental arm [11], the current NMA separately investigated the efficacy and acceptability of individual NIBS modalities in different brain regions. The rationale of this analytical strategy was based on our previous NMA, which noticed a priming effect [19] or additive effect [20] following the application of NIBS in different brain regions. Second, to avoid the methodological issues in previous NMAs [11, 12], we conducted the current comprehensive NMA with stricter inclusion criteria. To be specific, we only included RCTs with true randomization procedures, but not those with quasi‐randomization procedures [14, 15]. Furthermore, to avoid duplication, we only included RCTs [16, 17] of the same sample sources once [18]. Therefore, the current NMA is able to provide more updated, convincing, and comprehensive evidence than previous NMAs.

The rationale for the application of NIBS modalities over bilateral dorsal mPFC/ACC and bilateral DLPFC relies mainly on the dysfunctional cortical‐striatal‐thalamic‐cortical loop circuit discussed earlier [10], in which the DLPFC and OFC/ACC components have become the major targets of NIBS modalities to date. Further, the rationale of designation of dTMS over these regions relied on its merits of deep penetration. Specifically, the dTMS procedure could deeply penetrate to ACC regions so that it could stimulate both dorsal mPFC and ACC regions [7]. As we know, the ACC, which plays a role in thought‐motivation‐emotion integration [34], had been found to be dysfunctional in OCD patients [9]. On the other hand, the abnormal hypoactivity in the left DLPFC had been found in OCD patients in a previous positron emission tomography [35], which might support the hypothesis that the hypoactive DLPFC might lose its ability to inhibit striatal and thalamic neuronal activity related to OCD symptoms [18]. In addition, the application of excitatory NIBS modalities (i.e., high‐frequency TMS) might be associated with improvement in the effectiveness of synapses between the activated neurons of the target brain regions [36]. Therefore, currently available basic and clinical trials might support the rationale of application of excitatory stimulations over the bilateral prefrontal cortex and associated regions to manage OCD severity.

### 4.2 The Role of Inhibitory Stimulation Over the Right DLPFC

Another important finding of the current NMA was that the efficacy of inhibitory stimulation of the right DLPFC (i.e., Lf‐rTMS‐F4) was associated with both improvement in overall OCD severity and in mood‐related components (i.e., depression and anxiety symptoms). The experimental arm “Lf‐rTMS‐F4” in the current NMA indicated “1‐Hz rTMS over right DLPFC”. As we know, mood components also play an important role in OCD prognosis or therapeutic predictors [37]. Furthermore, the OCD symptoms and concurrent depression/anxiety symptoms had bidirectional and cross‐lagged relationship. That is, the improvement of concurrent depression/anxiety symptoms would also assist in improvement of OCD symptoms [38]. Therefore, to manage the mood components would be beneficial in OCD treatment. As we know, the Lf‐rTMS‐F4 exerted an inhibitory effect toward the right DLPFC and finally increased the activity at the striatum and thalamus [39], which could consequently modulate the aforementioned dysfunctional cortical‐striatal‐thalamic‐cortical loop circuit [10]. Therefore, the application of low‐frequency rTMS over right DLPFC can be effective in managing depression [40], anxiety symptoms [41], and, reciprocally, the OCD symptoms [42].

### 4.3 Limitations

This study had some limitations to be addressed. First, the NMA may have been underpowered because of the heterogeneity of the participants (e.g., comorbidities, concomitant psychotropic medication, baseline OCD severity, timing of NIBS intervention, definition of response rate, and follow‐up duration). Second, although most RCTs included a sham control in their study design, the blindness of the RCTs may not have been completely guaranteed because of the limitations of the commercial instruments employed. Third, both TMS sham controls and tDCS sham controls contributed to a significant alleviation of overall OCD severity. The significant placebo effect in these two sham therapy groups might be due to (1) the direct placebo effect of the sham therapy and (2) the potential therapeutic effect of concurrent psychotropic medication or psychotherapy on OCD symptoms [6, 7]. Fourth, the relatively short treatment duration and overall study duration (i.e., a mean treatment duration of 3.5 weeks and mean overall study duration of 8.8 weeks among the included RCTs) might limit the observation of treatment efficacy progression, because some patients undergoing neurostimulation would exhibit a delayed response phenomenon. Finally, the variation of the definition of response rate across the recruited RCTs would impose potential bias in the current NMA.

## 5 Conclusion

This NMA revealed that excitatory stimulation over the bilateral dmPFC/ACC or bilateral DLPFC or inhibitory stimulation over the right DLPFC was associated with a significant improvement in the overall severity of OCD. In addition, inhibitory stimulation of the right DLPFC was associated with the greatest improvement in mood‐related components (i.e., depression and anxiety symptoms). All investigated NIBS modalities were associated with acceptability, similar to the sham control group. Our findings provide a rationale for designing future large‐scale RCTs with longer follow‐up periods to investigate the association between NIBS and symptom severity in patients with OCD.

## Author Contributions

Ping‐Tao Tseng, Chih‐Wei Hsu, Chao‐Ming Hung, and Chih‐Sung Liang, who contributed equally as first authors, took the whole responsibility in literature search, data extraction, data analysis, and manuscript draft. Hung‐Yu Wang, Brendon Stubbs, Andre F. Carvalho, Andre R. Brunoni, Kuan‐Pin Su, Yu‐Kang Tu, Yi‐Cheng Wu, Tien‐Yu Chen, Dian‐Jeng Li, Pao‐Yen Lin, Yen‐Wen Chen, Kuo‐Chuan Hung, and Jiann‐Jy Chen contributed significantly in study design, concept formation, and major revision of manuscript. Ping‐Tao Tseng, Bing‐Syuan Zeng, and Cheng‐Ta Li, who contributed equally as corresponding authors, took the whole responsibility in communication, manuscript revision, and manuscript submission.

## Ethics Statement

The Institutional Review Board of the Tri‐Service General Hospital has confirmed that no ethical approval is required (TSGHIRB: B‐109‐29).

## Consent

The authors have nothing to report.

## Conflicts of Interest

The authors declare no conflicts of interest.

## Supporting information


Figures S1–S4.

Tables S1–S10.

## Data Availability

The data that support the findings of this study are available from the corresponding author upon reasonable request.
