# Supplementary material for: The Efficacy and Acceptability of Non‐Invasive Brain Stimulation Interventions for Obsessive‐Compulsive Disorder Management: A Network Meta‐Analysis Based on 24 Stimulation Methods
Source: Acta Psychiatr Scand. 2025 Mar 31;152(2):112–24. doi: 10.1111/acps.13809 (PMC12213009; doi:10.1111/acps.13809)
Supplement: Supplementary file 1 — Figures S1–S4. [file ACPS-152-112-s002.pptx]

## Slide 1
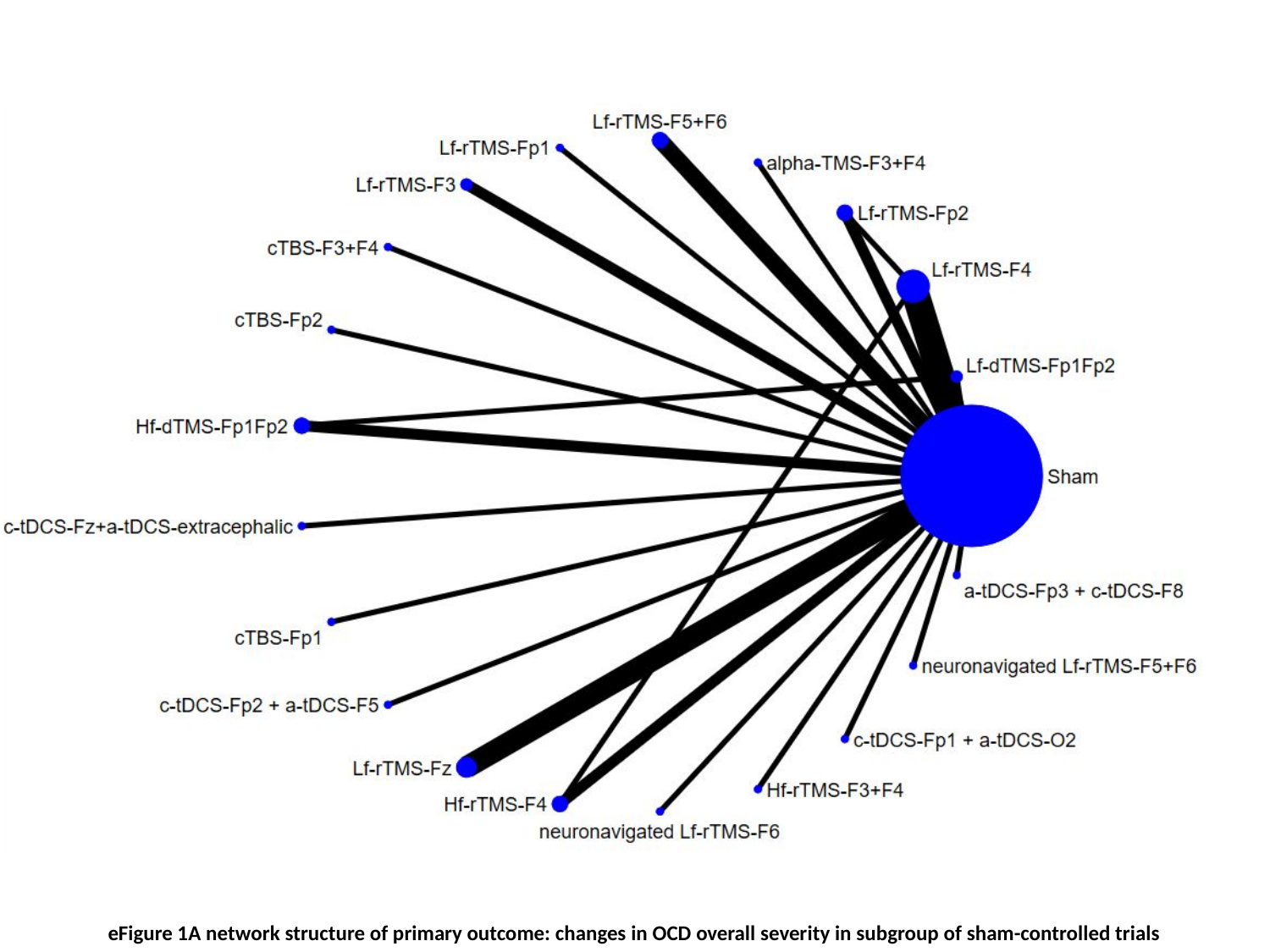

eFigure 1A network structure of primary outcome: changes in OCD overall severity in subgroup of sham-controlled trials

## Slide 2
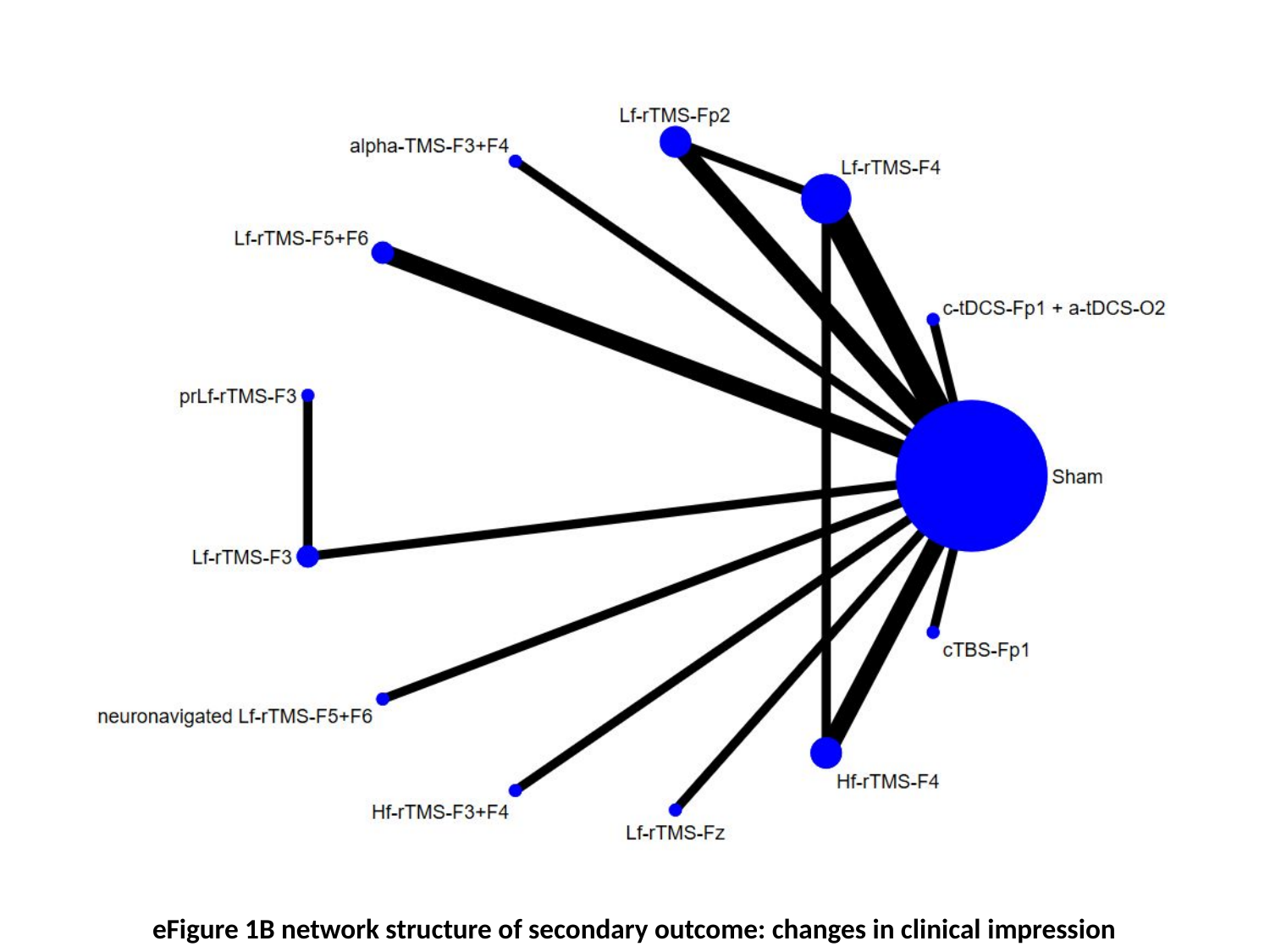

eFigure 1B network structure of secondary outcome: changes in clinical impression

## Slide 3
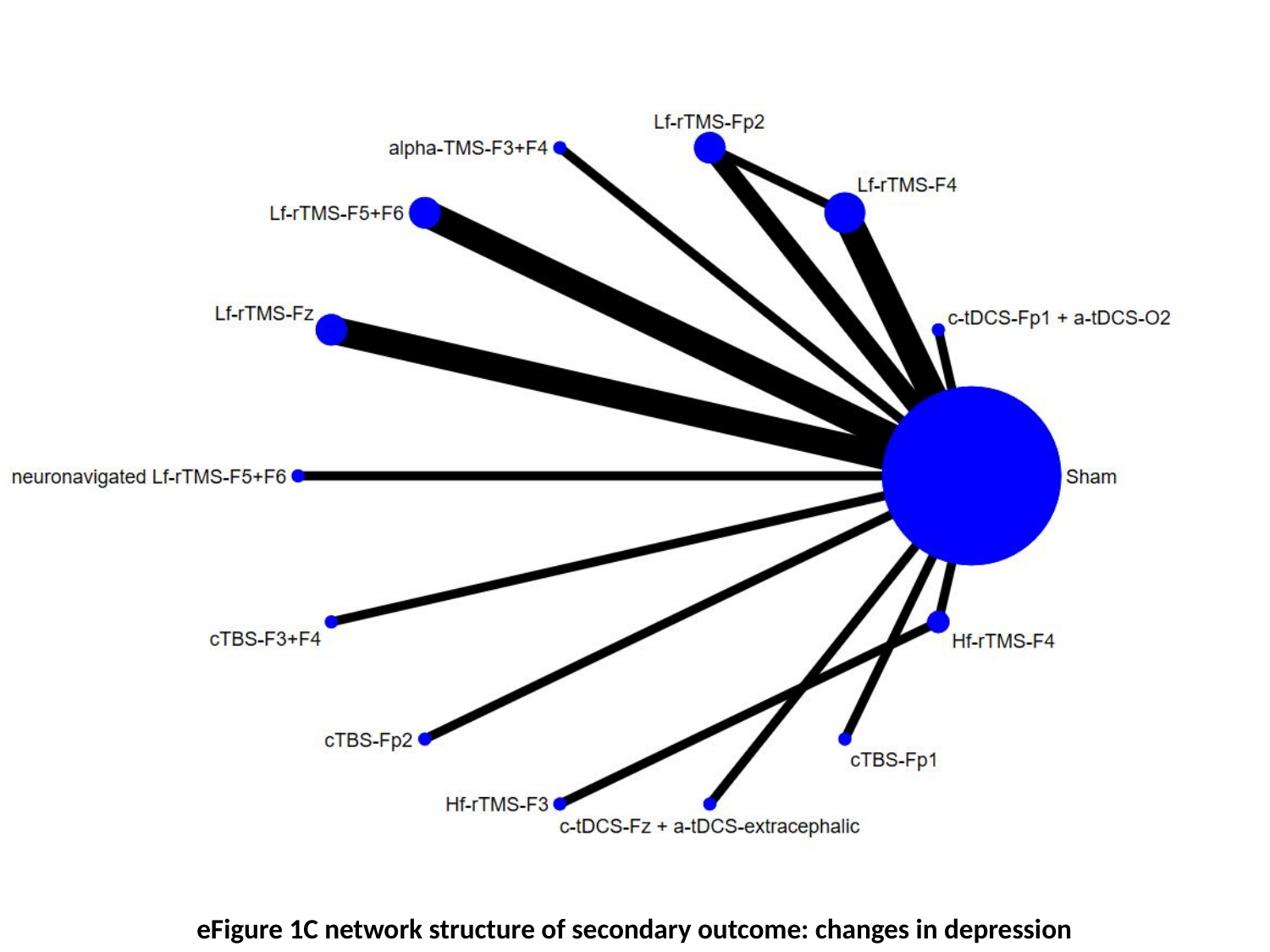

eFigure 1C network structure of secondary outcome: changes in depression

## Slide 4
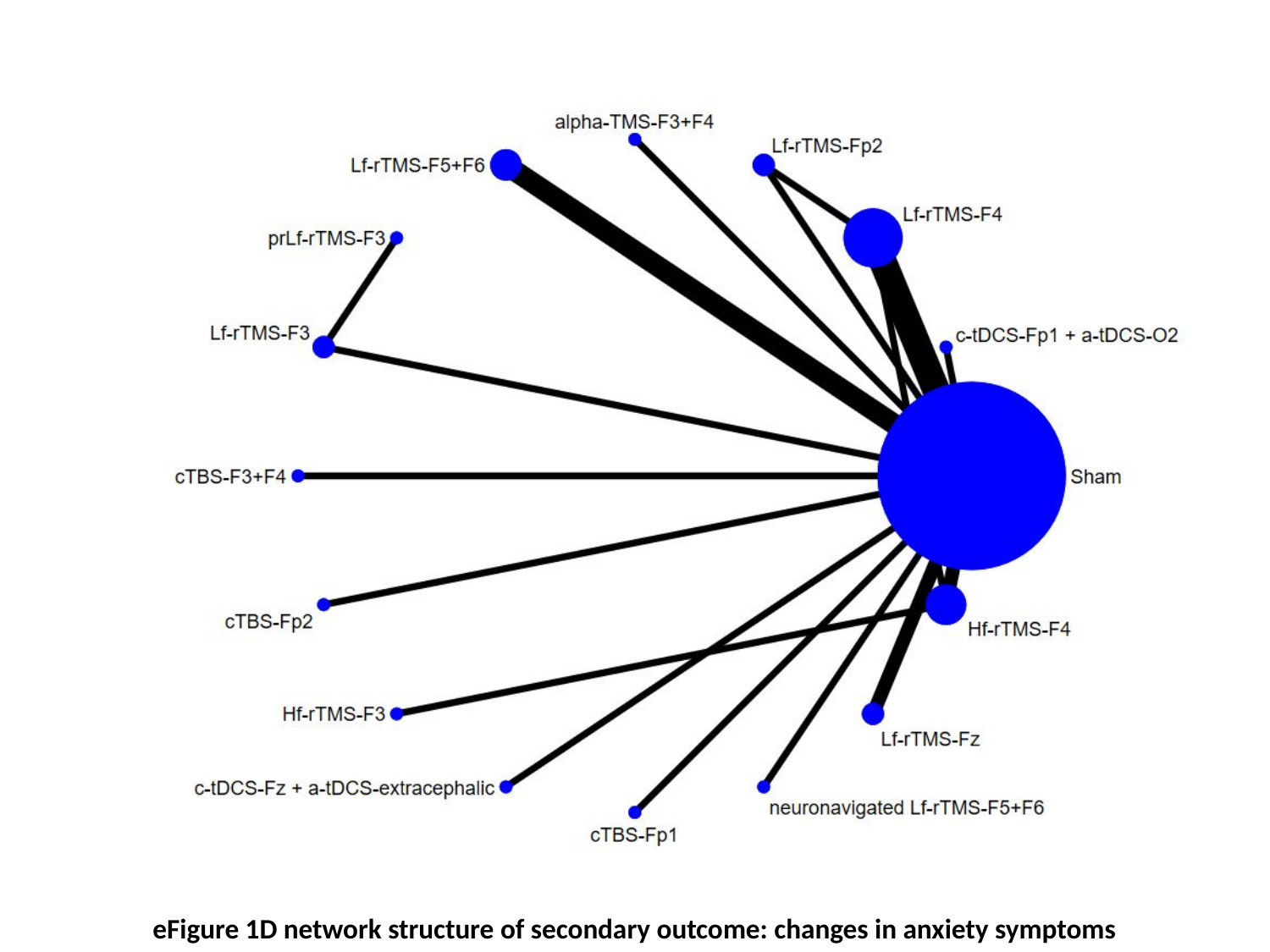

eFigure 1D network structure of secondary outcome: changes in anxiety symptoms

## Slide 5
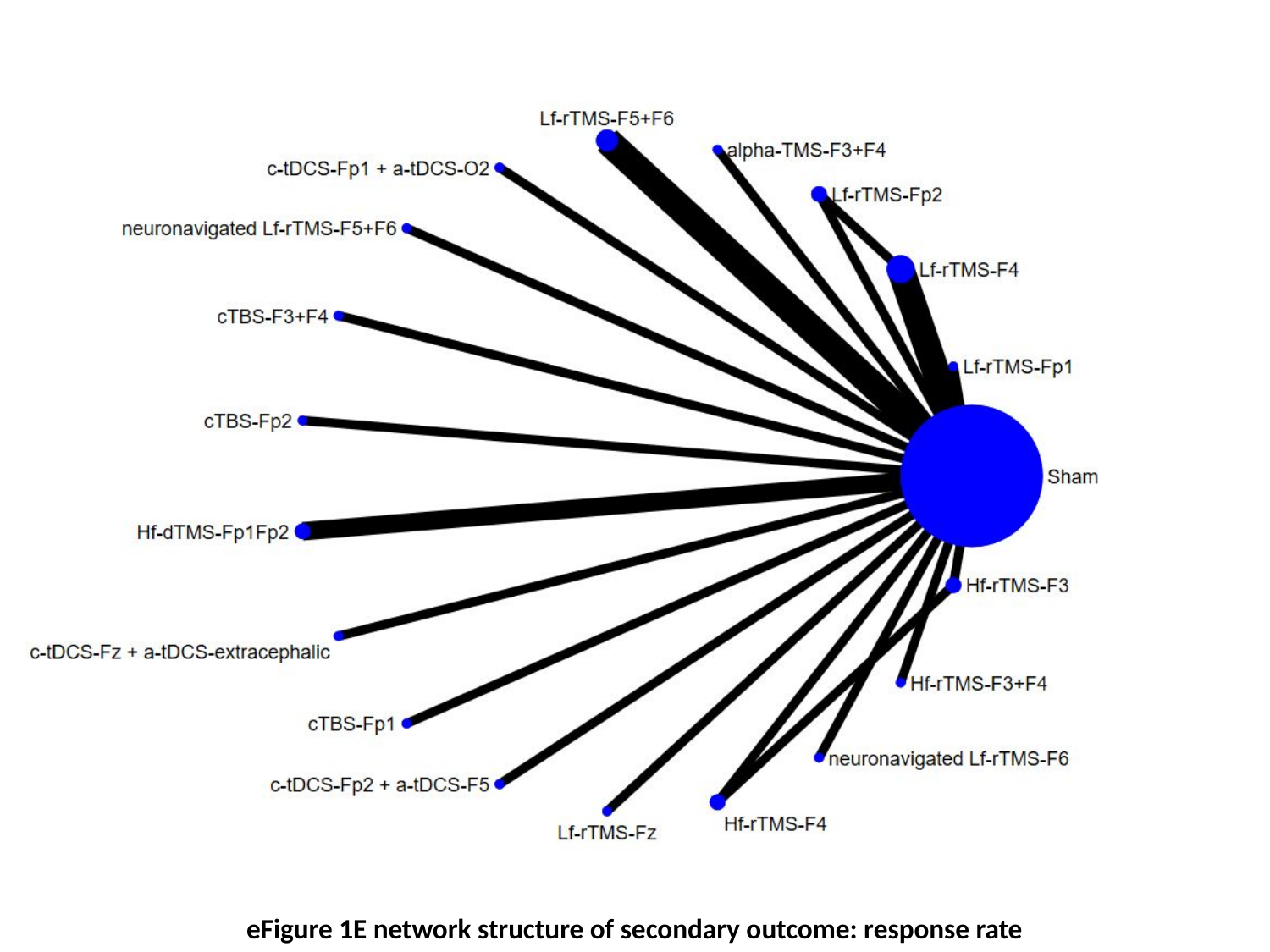

eFigure 1E network structure of secondary outcome: response rate

## Slide 6
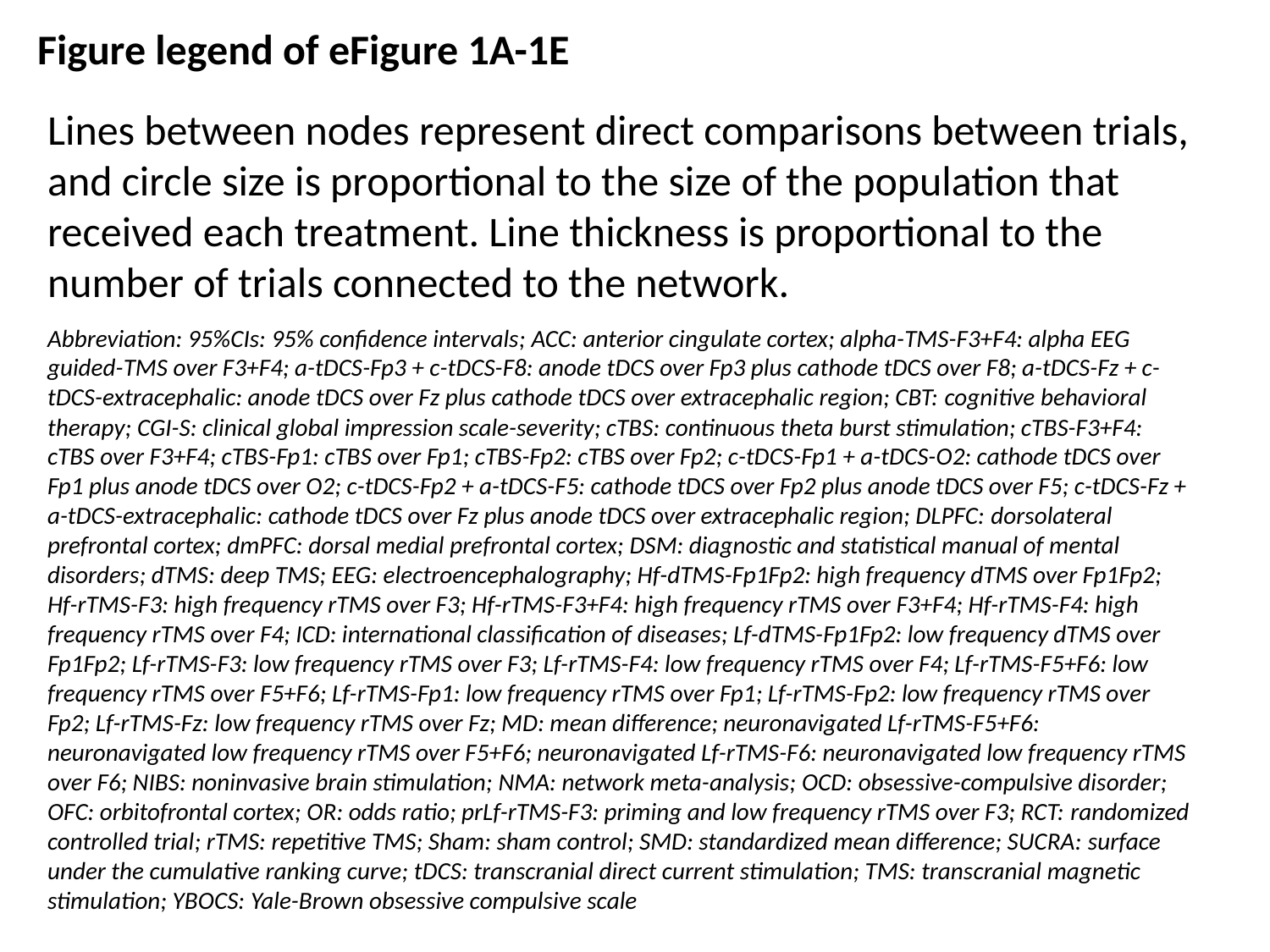

Figure legend of eFigure 1A-1E
Lines between nodes represent direct comparisons between trials, and circle size is proportional to the size of the population that received each treatment. Line thickness is proportional to the number of trials connected to the network.
Abbreviation: 95%CIs: 95% confidence intervals; ACC: anterior cingulate cortex; alpha-TMS-F3+F4: alpha EEG guided-TMS over F3+F4; a-tDCS-Fp3 + c-tDCS-F8: anode tDCS over Fp3 plus cathode tDCS over F8; a-tDCS-Fz + c-tDCS-extracephalic: anode tDCS over Fz plus cathode tDCS over extracephalic region; CBT: cognitive behavioral therapy; CGI-S: clinical global impression scale-severity; cTBS: continuous theta burst stimulation; cTBS-F3+F4: cTBS over F3+F4; cTBS-Fp1: cTBS over Fp1; cTBS-Fp2: cTBS over Fp2; c-tDCS-Fp1 + a-tDCS-O2: cathode tDCS over Fp1 plus anode tDCS over O2; c-tDCS-Fp2 + a-tDCS-F5: cathode tDCS over Fp2 plus anode tDCS over F5; c-tDCS-Fz + a-tDCS-extracephalic: cathode tDCS over Fz plus anode tDCS over extracephalic region; DLPFC: dorsolateral prefrontal cortex; dmPFC: dorsal medial prefrontal cortex; DSM: diagnostic and statistical manual of mental disorders; dTMS: deep TMS; EEG: electroencephalography; Hf-dTMS-Fp1Fp2: high frequency dTMS over Fp1Fp2; Hf-rTMS-F3: high frequency rTMS over F3; Hf-rTMS-F3+F4: high frequency rTMS over F3+F4; Hf-rTMS-F4: high frequency rTMS over F4; ICD: international classification of diseases; Lf-dTMS-Fp1Fp2: low frequency dTMS over Fp1Fp2; Lf-rTMS-F3: low frequency rTMS over F3; Lf-rTMS-F4: low frequency rTMS over F4; Lf-rTMS-F5+F6: low frequency rTMS over F5+F6; Lf-rTMS-Fp1: low frequency rTMS over Fp1; Lf-rTMS-Fp2: low frequency rTMS over Fp2; Lf-rTMS-Fz: low frequency rTMS over Fz; MD: mean difference; neuronavigated Lf-rTMS-F5+F6: neuronavigated low frequency rTMS over F5+F6; neuronavigated Lf-rTMS-F6: neuronavigated low frequency rTMS over F6; NIBS: noninvasive brain stimulation; NMA: network meta-analysis; OCD: obsessive-compulsive disorder; OFC: orbitofrontal cortex; OR: odds ratio; prLf-rTMS-F3: priming and low frequency rTMS over F3; RCT: randomized controlled trial; rTMS: repetitive TMS; Sham: sham control; SMD: standardized mean difference; SUCRA: surface under the cumulative ranking curve; tDCS: transcranial direct current stimulation; TMS: transcranial magnetic stimulation; YBOCS: Yale-Brown obsessive compulsive scale

## Slide 7
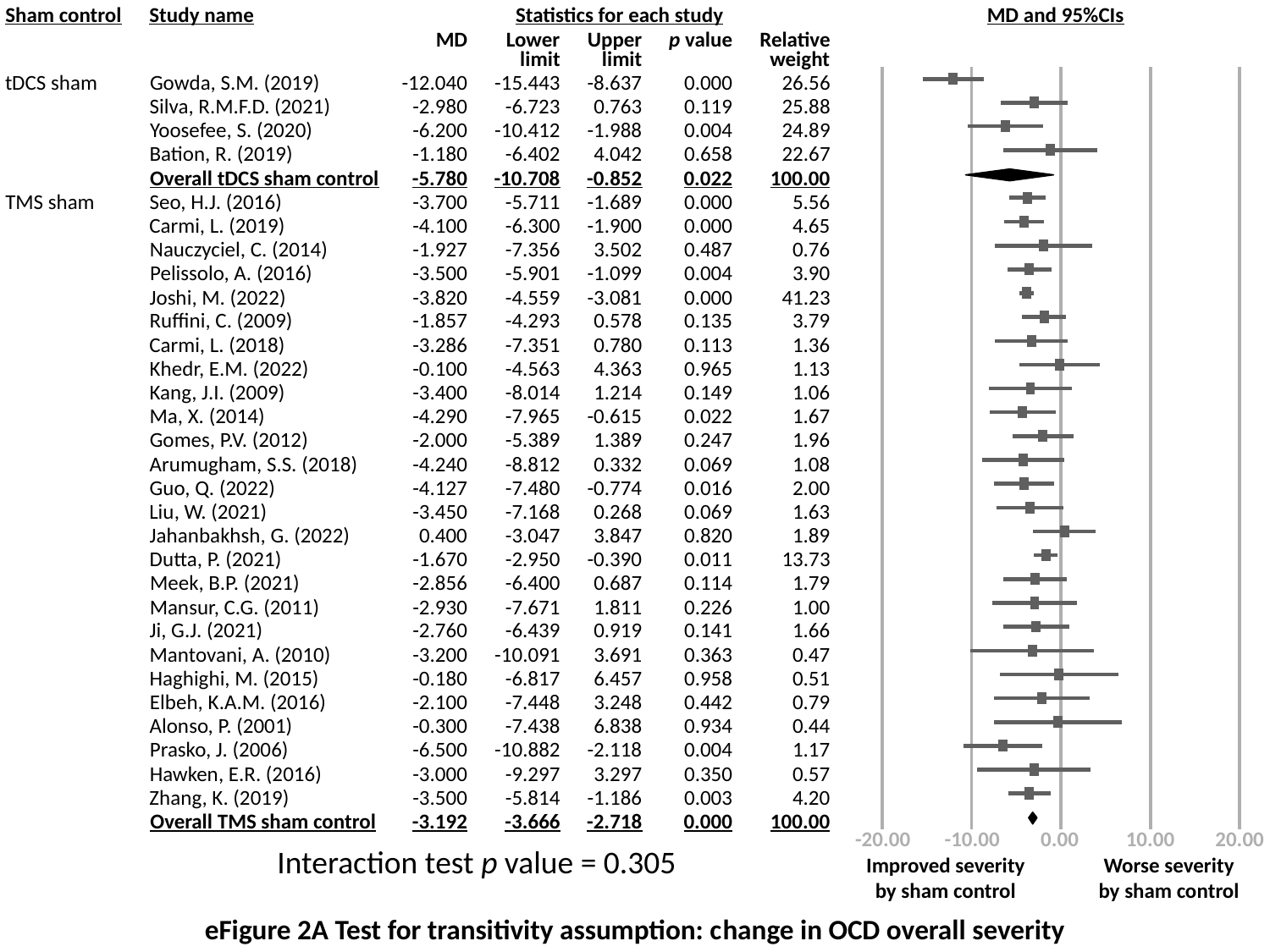

Sham control
Study name
Statistics for each study
MD and 95%CIs
MD
Lower
Upper
p value
Relative
limit
limit
weight
tDCS sham
Gowda, S.M. (2019)
-12.040
-15.443
-8.637
0.000
26.56
Silva, R.M.F.D. (2021)
-2.980
-6.723
0.763
0.119
25.88
Yoosefee, S. (2020)
-6.200
-10.412
-1.988
0.004
24.89
Bation, R. (2019)
-1.180
-6.402
4.042
0.658
22.67
Overall tDCS sham control
-5.780
-10.708
-0.852
0.022
100.00
TMS sham
Seo, H.J. (2016)
-3.700
-5.711
-1.689
0.000
5.56
Carmi, L. (2019)
-4.100
-6.300
-1.900
0.000
4.65
Nauczyciel, C. (2014)
-1.927
-7.356
3.502
0.487
0.76
Pelissolo, A. (2016)
-3.500
-5.901
-1.099
0.004
3.90
Joshi, M. (2022)
-3.820
-4.559
-3.081
0.000
41.23
Ruffini, C. (2009)
-1.857
-4.293
0.578
0.135
3.79
Carmi, L. (2018)
-3.286
-7.351
0.780
0.113
1.36
Khedr, E.M. (2022)
-0.100
-4.563
4.363
0.965
1.13
Kang, J.I. (2009)
-3.400
-8.014
1.214
0.149
1.06
Ma, X. (2014)
-4.290
-7.965
-0.615
0.022
1.67
Gomes, P.V. (2012)
-2.000
-5.389
1.389
0.247
1.96
Arumugham, S.S. (2018)
-4.240
-8.812
0.332
0.069
1.08
Guo, Q. (2022)
-4.127
-7.480
-0.774
0.016
2.00
Liu, W. (2021)
-3.450
-7.168
0.268
0.069
1.63
Jahanbakhsh, G. (2022)
0.400
-3.047
3.847
0.820
1.89
Dutta, P. (2021)
-1.670
-2.950
-0.390
0.011
13.73
Meek, B.P. (2021)
-2.856
-6.400
0.687
0.114
1.79
Mansur, C.G. (2011)
-2.930
-7.671
1.811
0.226
1.00
Ji, G.J. (2021)
-2.760
-6.439
0.919
0.141
1.66
Mantovani, A. (2010)
-3.200
-10.091
3.691
0.363
0.47
Haghighi, M. (2015)
-0.180
-6.817
6.457
0.958
0.51
Elbeh, K.A.M. (2016)
-2.100
-7.448
3.248
0.442
0.79
Alonso, P. (2001)
-0.300
-7.438
6.838
0.934
0.44
Prasko, J. (2006)
-6.500
-10.882
-2.118
0.004
1.17
Hawken, E.R. (2016)
-3.000
-9.297
3.297
0.350
0.57
Zhang, K. (2019)
-3.500
-5.814
-1.186
0.003
4.20
Overall TMS sham control
-3.192
-3.666
-2.718
0.000
100.00
-20.00
-10.00
0.00
10.00
20.00
Interaction test p value = 0.305
Improved severity by sham control
Worse severity by sham control
eFigure 2A Test for transitivity assumption: change in OCD overall severity

## Slide 8
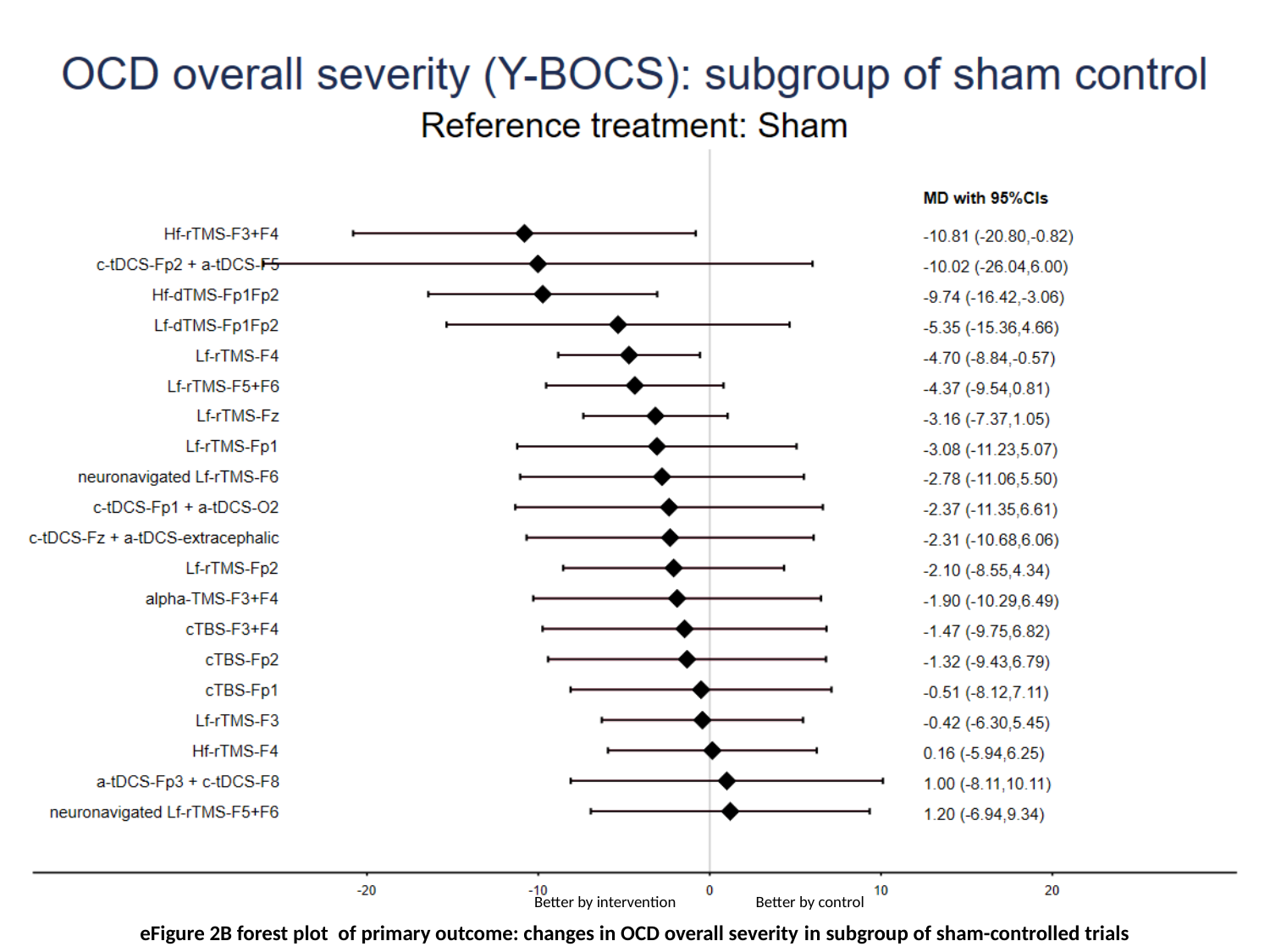

Better by intervention
Better by control
eFigure 2B forest plot of primary outcome: changes in OCD overall severity in subgroup of sham-controlled trials

## Slide 9
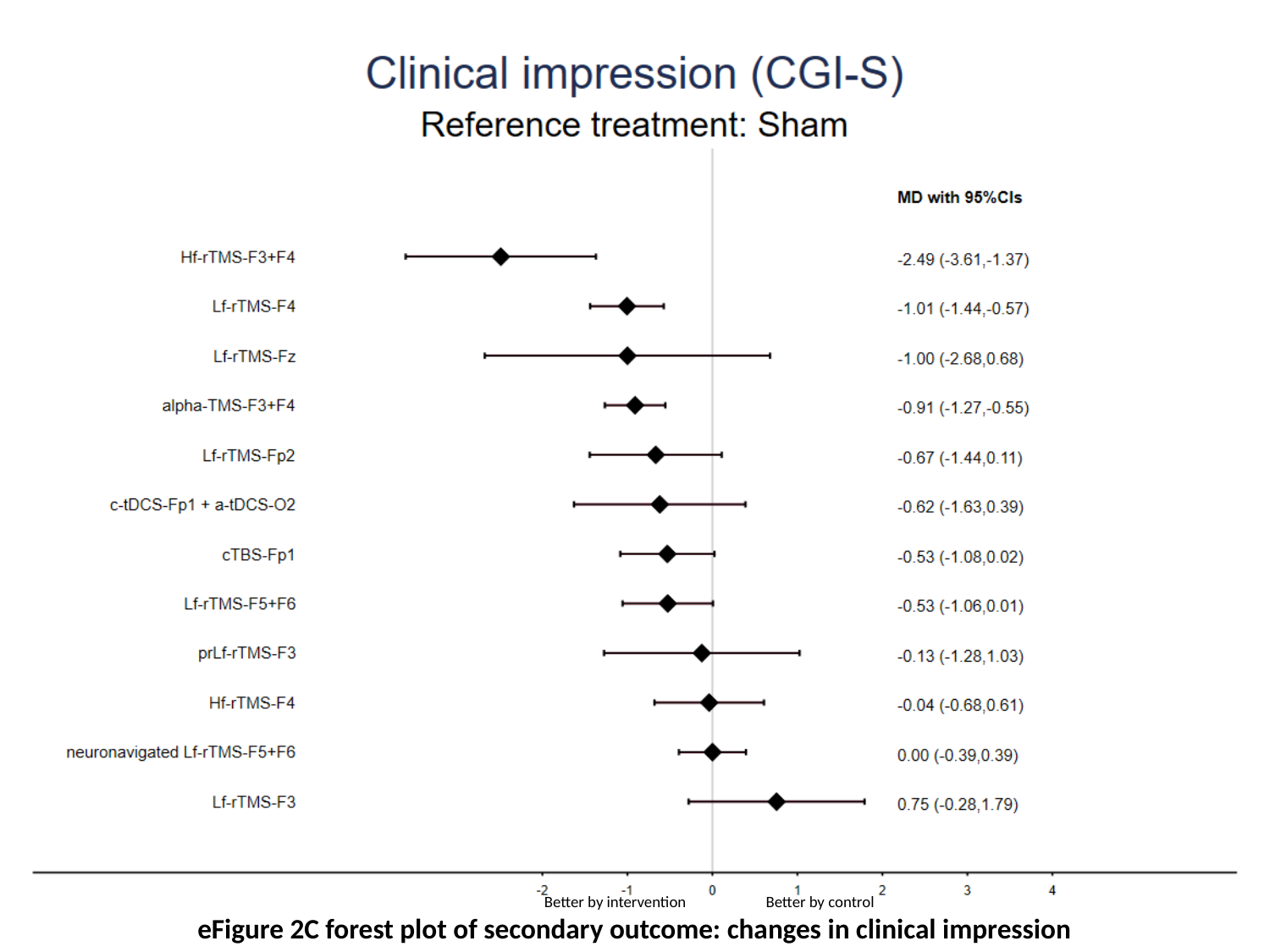

Better by intervention
Better by control
eFigure 2C forest plot of secondary outcome: changes in clinical impression

## Slide 10
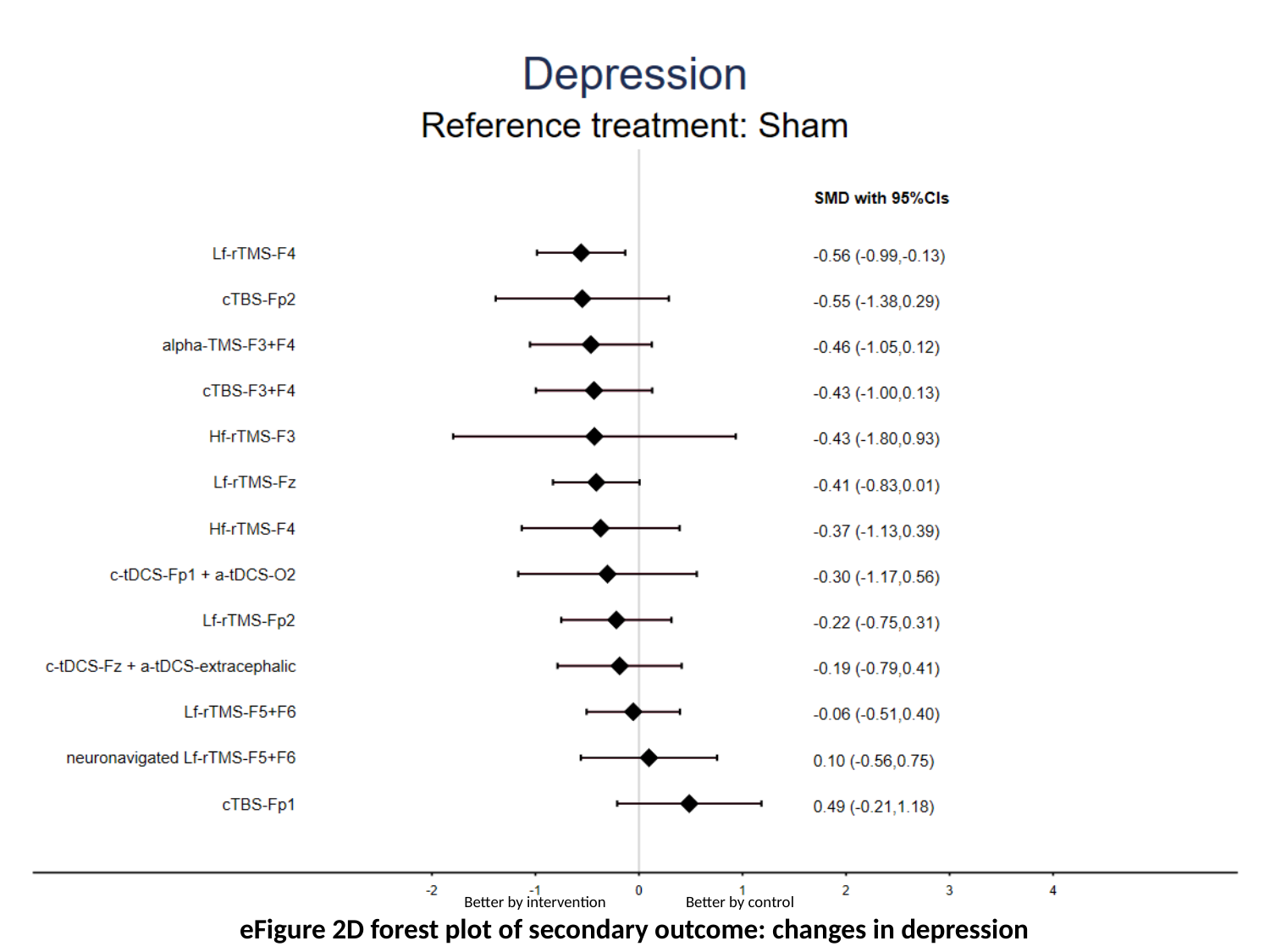

Better by intervention
Better by control
eFigure 2D forest plot of secondary outcome: changes in depression

## Slide 11
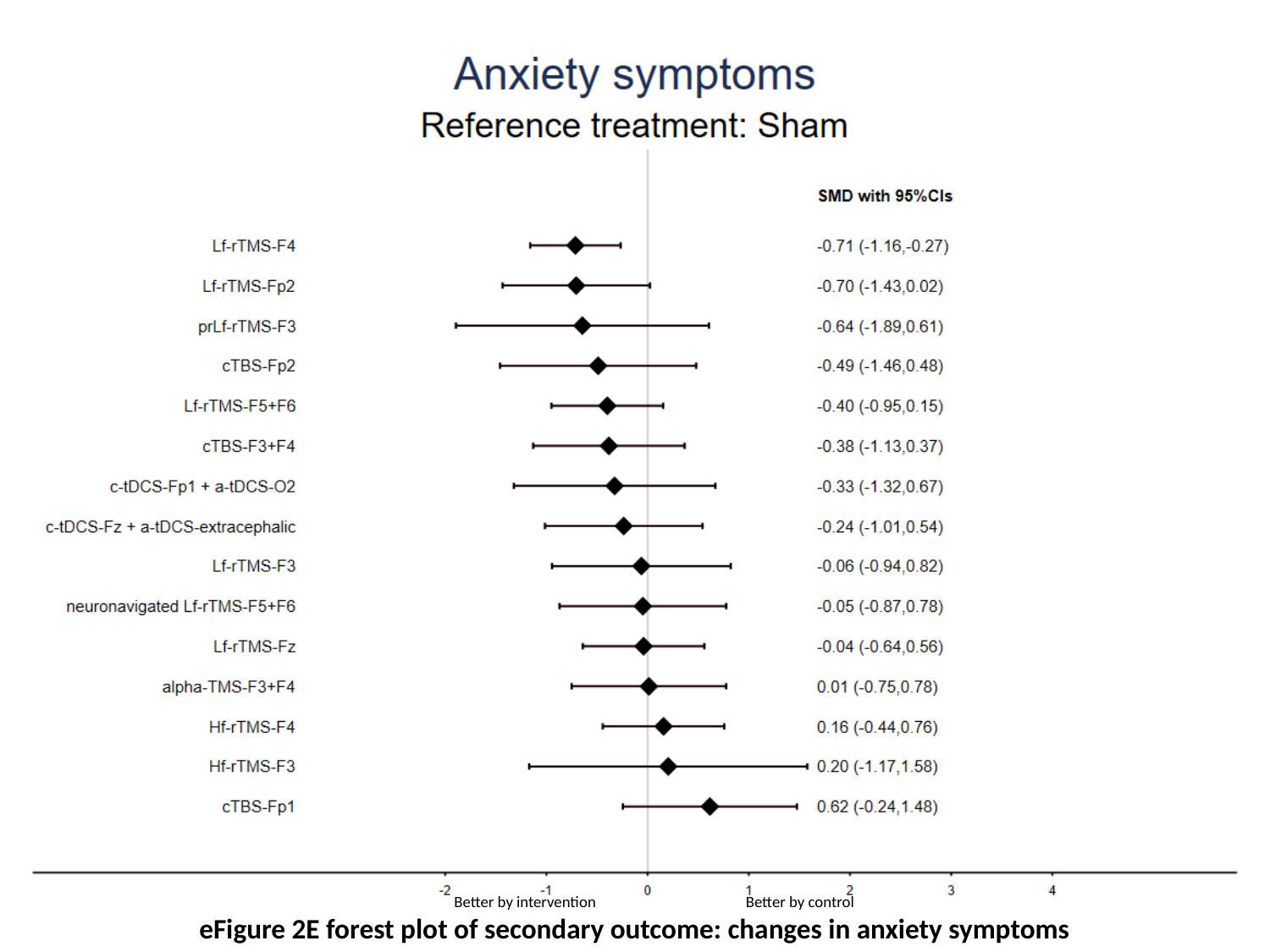

Better by intervention
Better by control
eFigure 2E forest plot of secondary outcome: changes in anxiety symptoms

## Slide 12
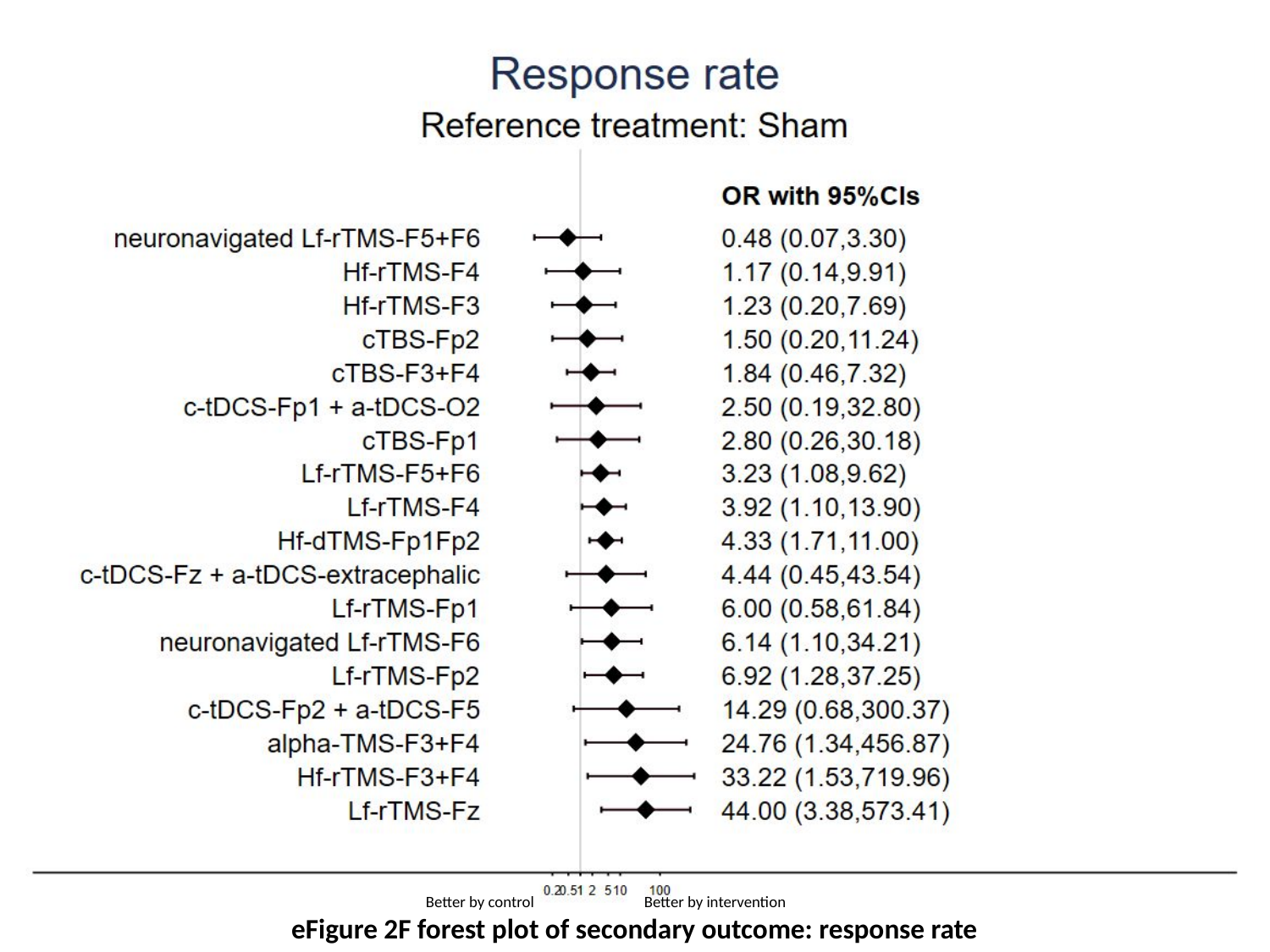

Better by control
Better by intervention
eFigure 2F forest plot of secondary outcome: response rate

## Slide 13
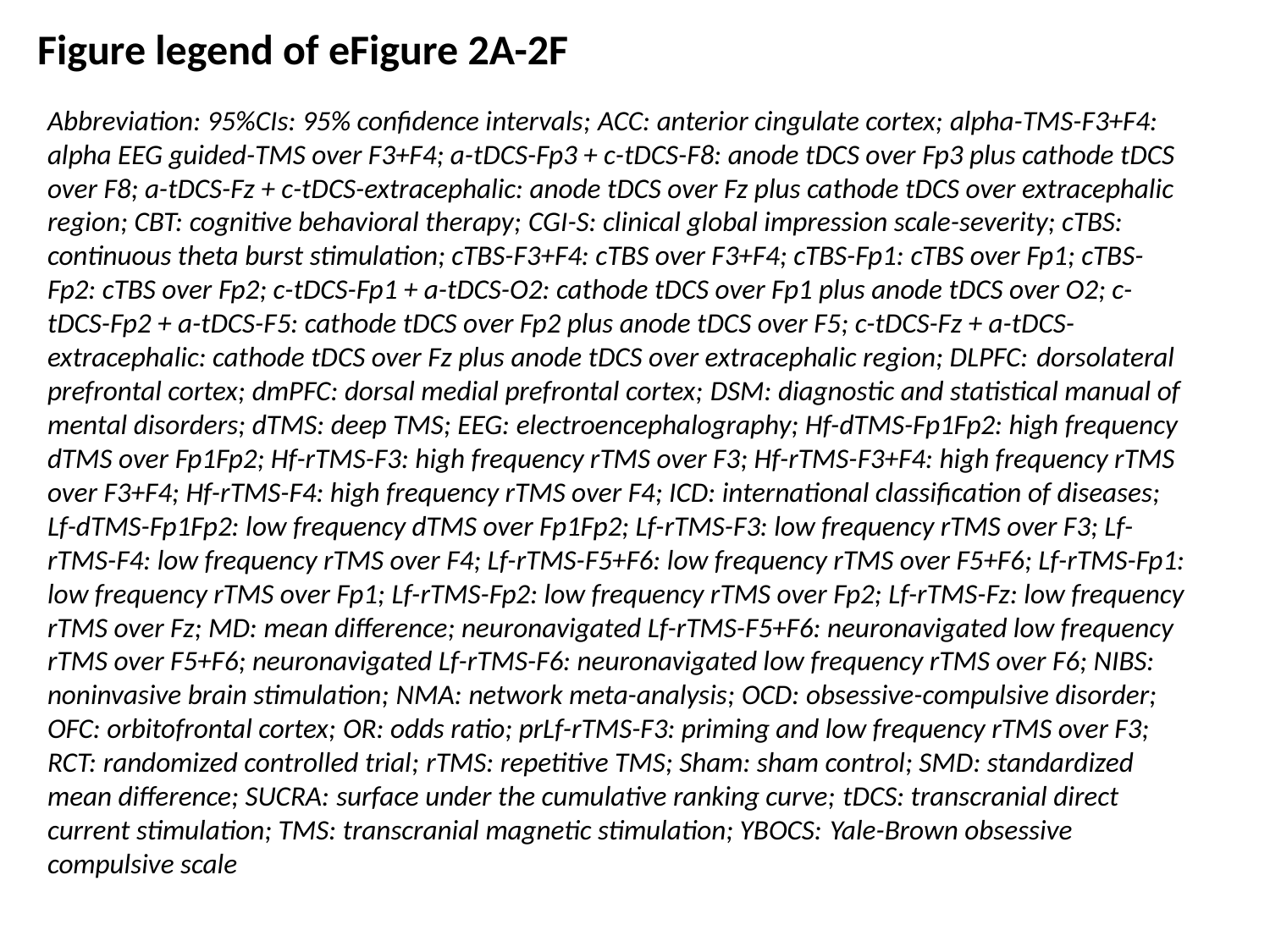

Figure legend of eFigure 2A-2F
Abbreviation: 95%CIs: 95% confidence intervals; ACC: anterior cingulate cortex; alpha-TMS-F3+F4: alpha EEG guided-TMS over F3+F4; a-tDCS-Fp3 + c-tDCS-F8: anode tDCS over Fp3 plus cathode tDCS over F8; a-tDCS-Fz + c-tDCS-extracephalic: anode tDCS over Fz plus cathode tDCS over extracephalic region; CBT: cognitive behavioral therapy; CGI-S: clinical global impression scale-severity; cTBS: continuous theta burst stimulation; cTBS-F3+F4: cTBS over F3+F4; cTBS-Fp1: cTBS over Fp1; cTBS-Fp2: cTBS over Fp2; c-tDCS-Fp1 + a-tDCS-O2: cathode tDCS over Fp1 plus anode tDCS over O2; c-tDCS-Fp2 + a-tDCS-F5: cathode tDCS over Fp2 plus anode tDCS over F5; c-tDCS-Fz + a-tDCS-extracephalic: cathode tDCS over Fz plus anode tDCS over extracephalic region; DLPFC: dorsolateral prefrontal cortex; dmPFC: dorsal medial prefrontal cortex; DSM: diagnostic and statistical manual of mental disorders; dTMS: deep TMS; EEG: electroencephalography; Hf-dTMS-Fp1Fp2: high frequency dTMS over Fp1Fp2; Hf-rTMS-F3: high frequency rTMS over F3; Hf-rTMS-F3+F4: high frequency rTMS over F3+F4; Hf-rTMS-F4: high frequency rTMS over F4; ICD: international classification of diseases; Lf-dTMS-Fp1Fp2: low frequency dTMS over Fp1Fp2; Lf-rTMS-F3: low frequency rTMS over F3; Lf-rTMS-F4: low frequency rTMS over F4; Lf-rTMS-F5+F6: low frequency rTMS over F5+F6; Lf-rTMS-Fp1: low frequency rTMS over Fp1; Lf-rTMS-Fp2: low frequency rTMS over Fp2; Lf-rTMS-Fz: low frequency rTMS over Fz; MD: mean difference; neuronavigated Lf-rTMS-F5+F6: neuronavigated low frequency rTMS over F5+F6; neuronavigated Lf-rTMS-F6: neuronavigated low frequency rTMS over F6; NIBS: noninvasive brain stimulation; NMA: network meta-analysis; OCD: obsessive-compulsive disorder; OFC: orbitofrontal cortex; OR: odds ratio; prLf-rTMS-F3: priming and low frequency rTMS over F3; RCT: randomized controlled trial; rTMS: repetitive TMS; Sham: sham control; SMD: standardized mean difference; SUCRA: surface under the cumulative ranking curve; tDCS: transcranial direct current stimulation; TMS: transcranial magnetic stimulation; YBOCS: Yale-Brown obsessive compulsive scale

## Slide 14
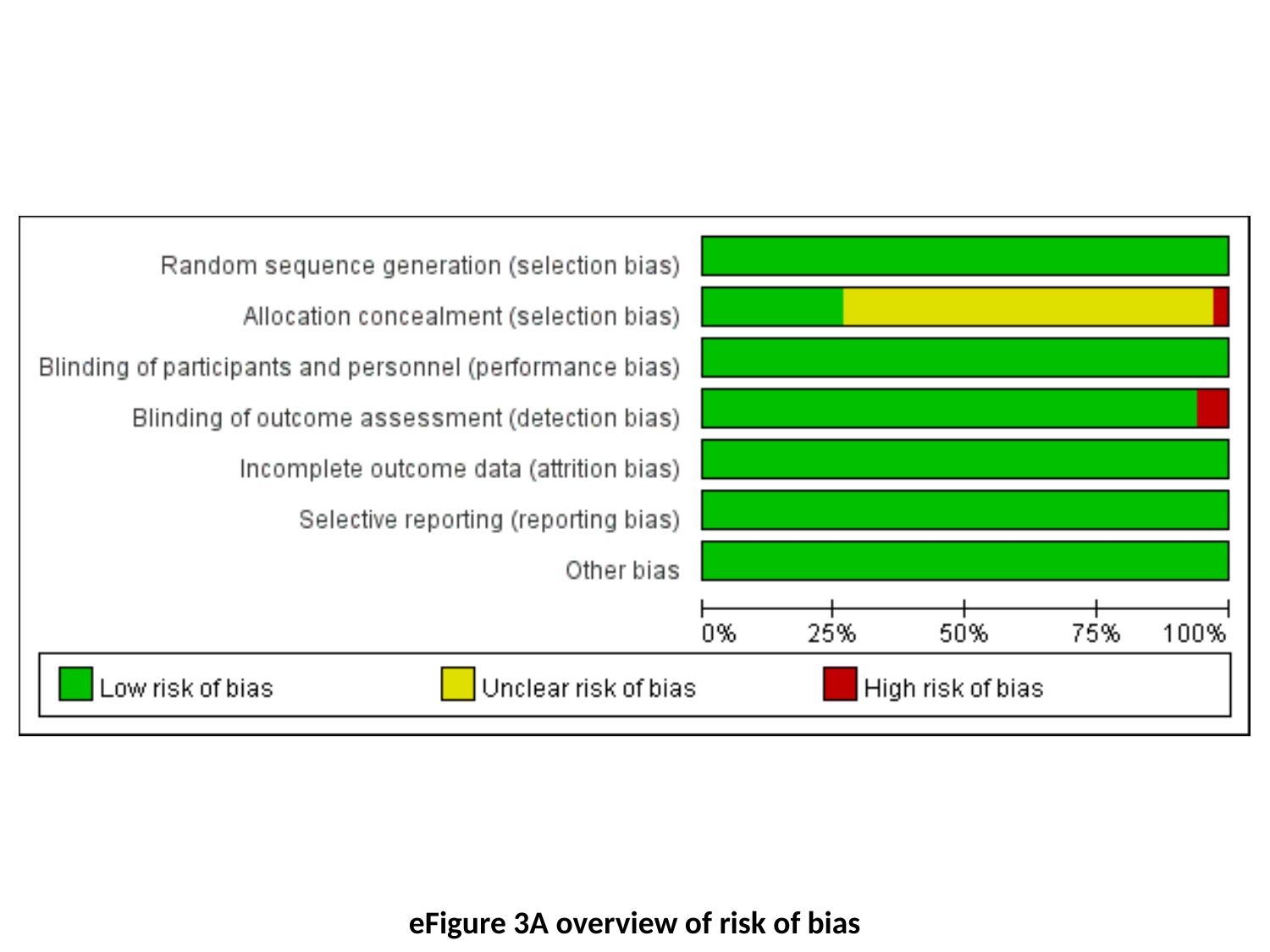

eFigure 3A overview of risk of bias

## Slide 15
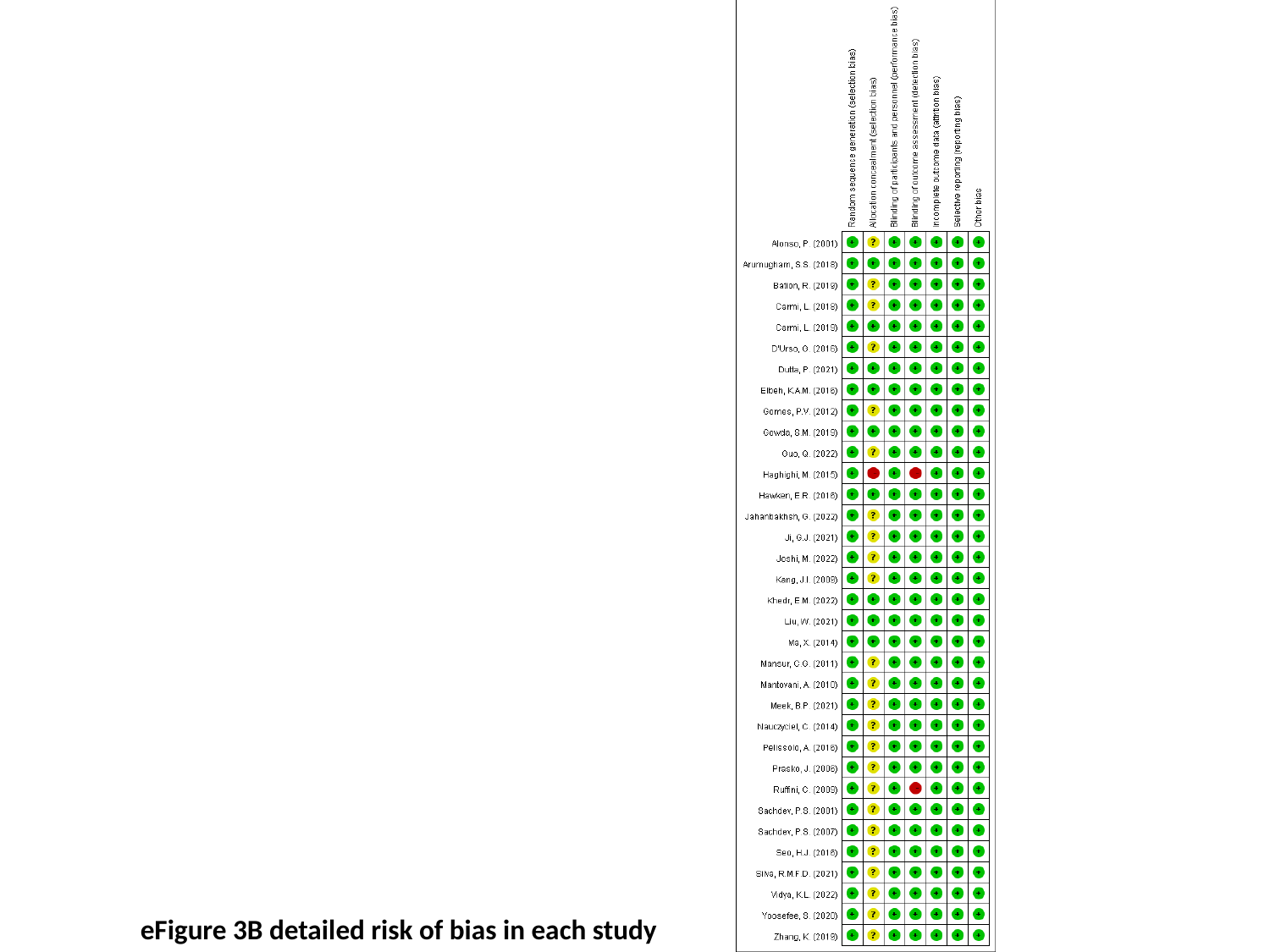

eFigure 3B detailed risk of bias in each study

## Slide 16
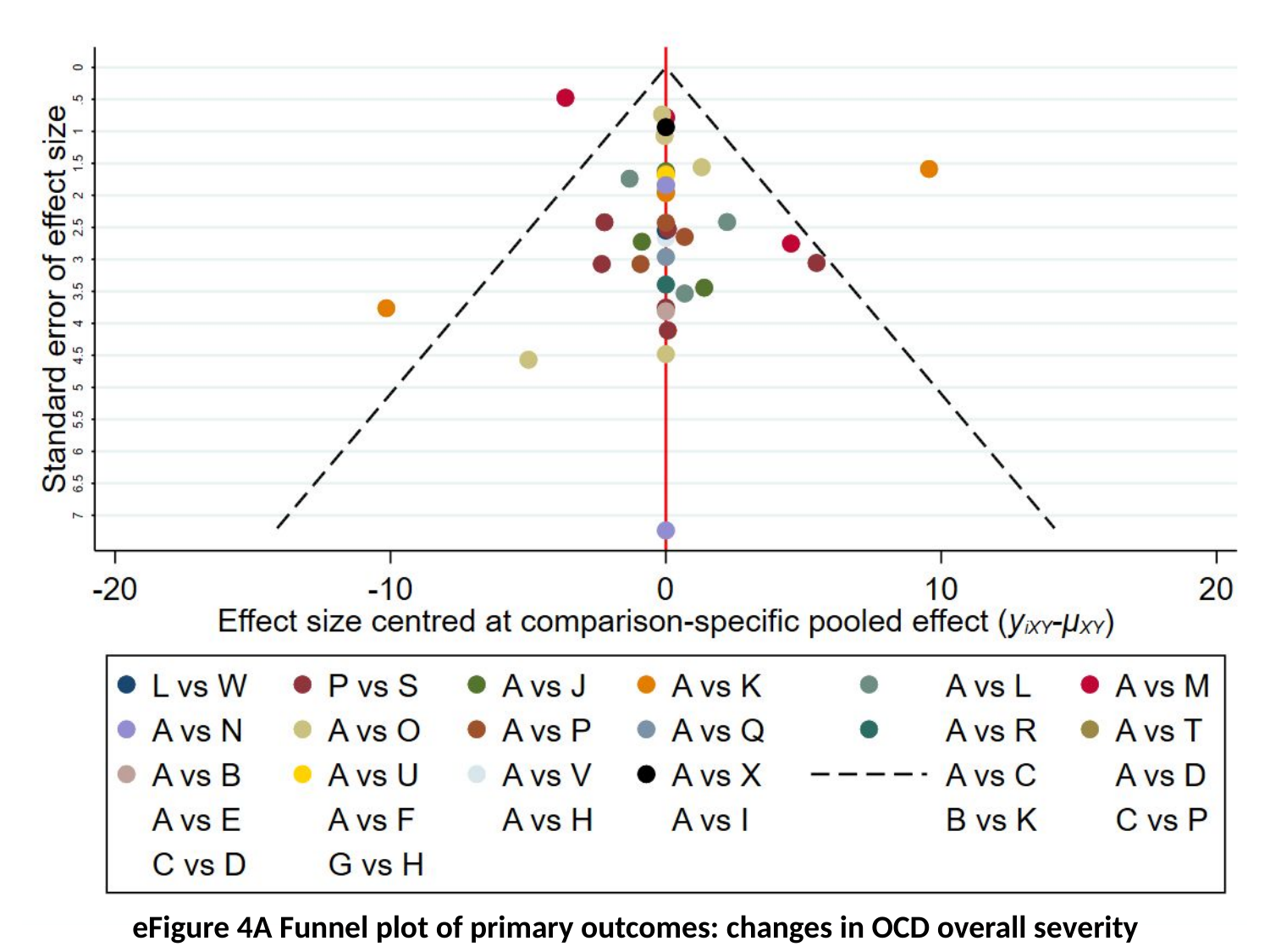

eFigure 4A Funnel plot of primary outcomes: changes in OCD overall severity

## Slide 17
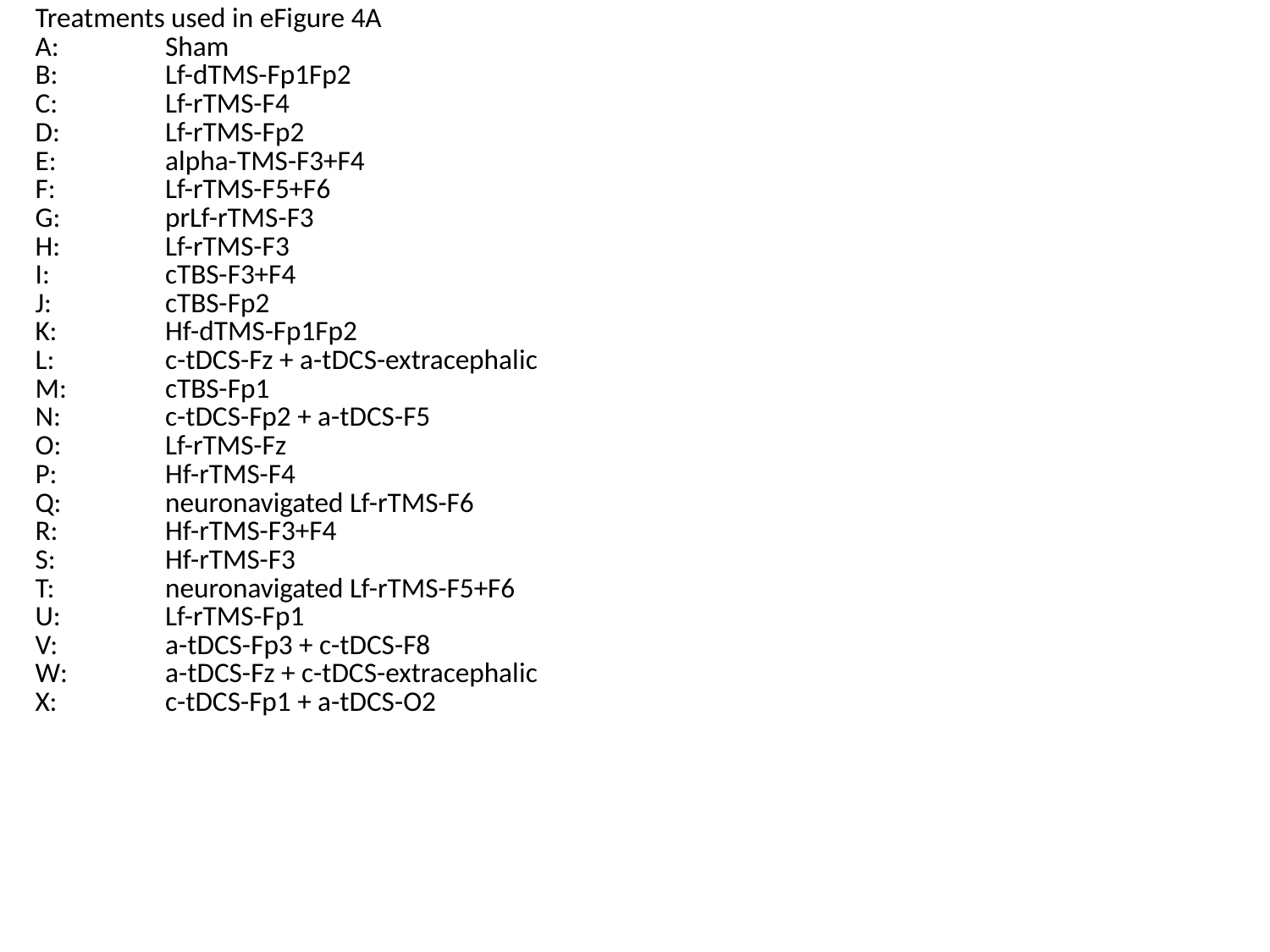

| Treatments used in eFigure 4A | |
| --- | --- |
| A: | Sham |
| B: | Lf-dTMS-Fp1Fp2 |
| C: | Lf-rTMS-F4 |
| D: | Lf-rTMS-Fp2 |
| E: | alpha-TMS-F3+F4 |
| F: | Lf-rTMS-F5+F6 |
| G: | prLf-rTMS-F3 |
| H: | Lf-rTMS-F3 |
| I: | cTBS-F3+F4 |
| J: | cTBS-Fp2 |
| K: | Hf-dTMS-Fp1Fp2 |
| L: | c-tDCS-Fz + a-tDCS-extracephalic |
| M: | cTBS-Fp1 |
| N: | c-tDCS-Fp2 + a-tDCS-F5 |
| O: | Lf-rTMS-Fz |
| P: | Hf-rTMS-F4 |
| Q: | neuronavigated Lf-rTMS-F6 |
| R: | Hf-rTMS-F3+F4 |
| S: | Hf-rTMS-F3 |
| T: | neuronavigated Lf-rTMS-F5+F6 |
| U: | Lf-rTMS-Fp1 |
| V: | a-tDCS-Fp3 + c-tDCS-F8 |
| W: | a-tDCS-Fz + c-tDCS-extracephalic |
| X: | c-tDCS-Fp1 + a-tDCS-O2 |

## Slide 18
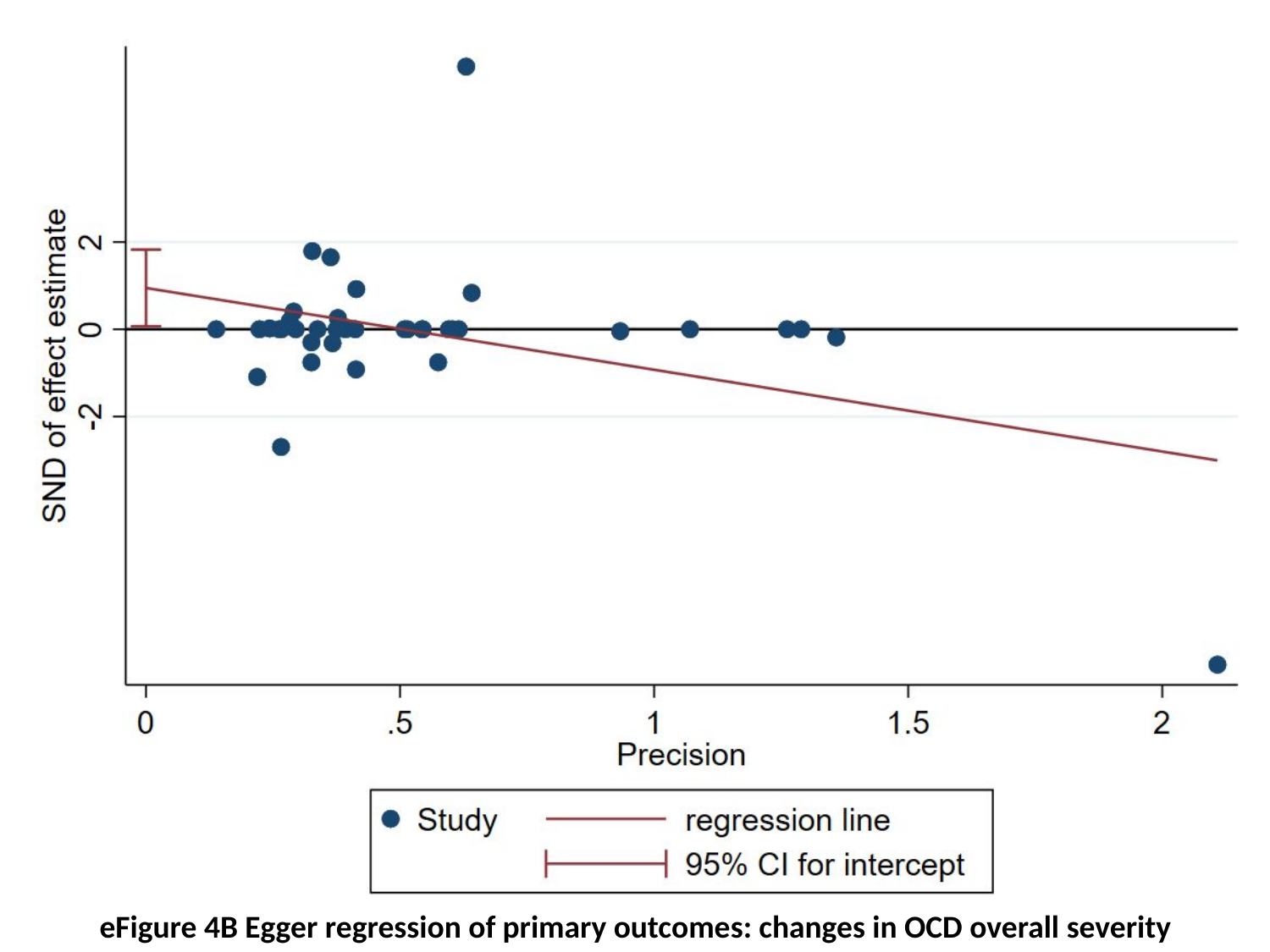

eFigure 4B Egger regression of primary outcomes: changes in OCD overall severity

## Slide 19
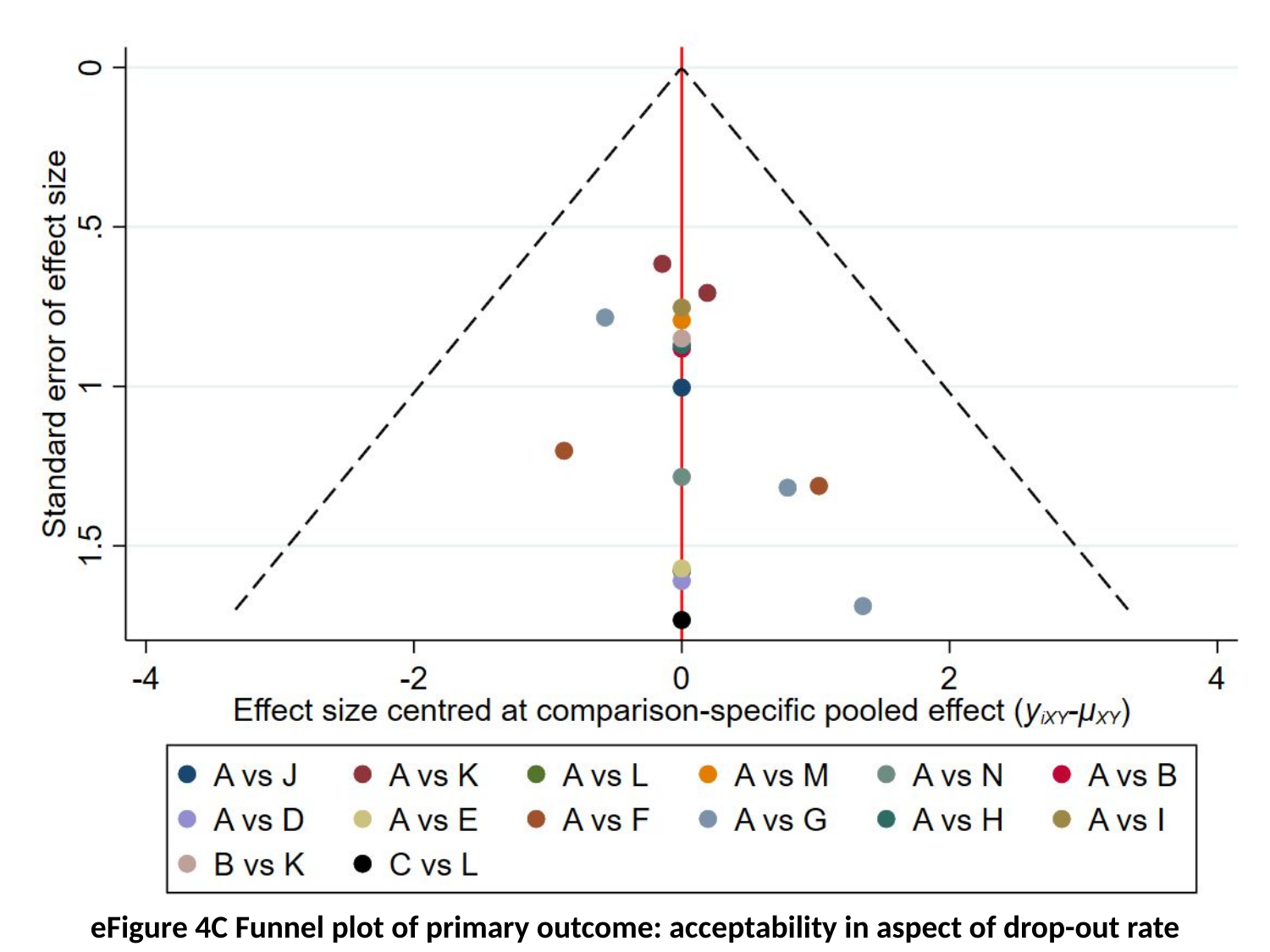

eFigure 4C Funnel plot of primary outcome: acceptability in aspect of drop-out rate

## Slide 20
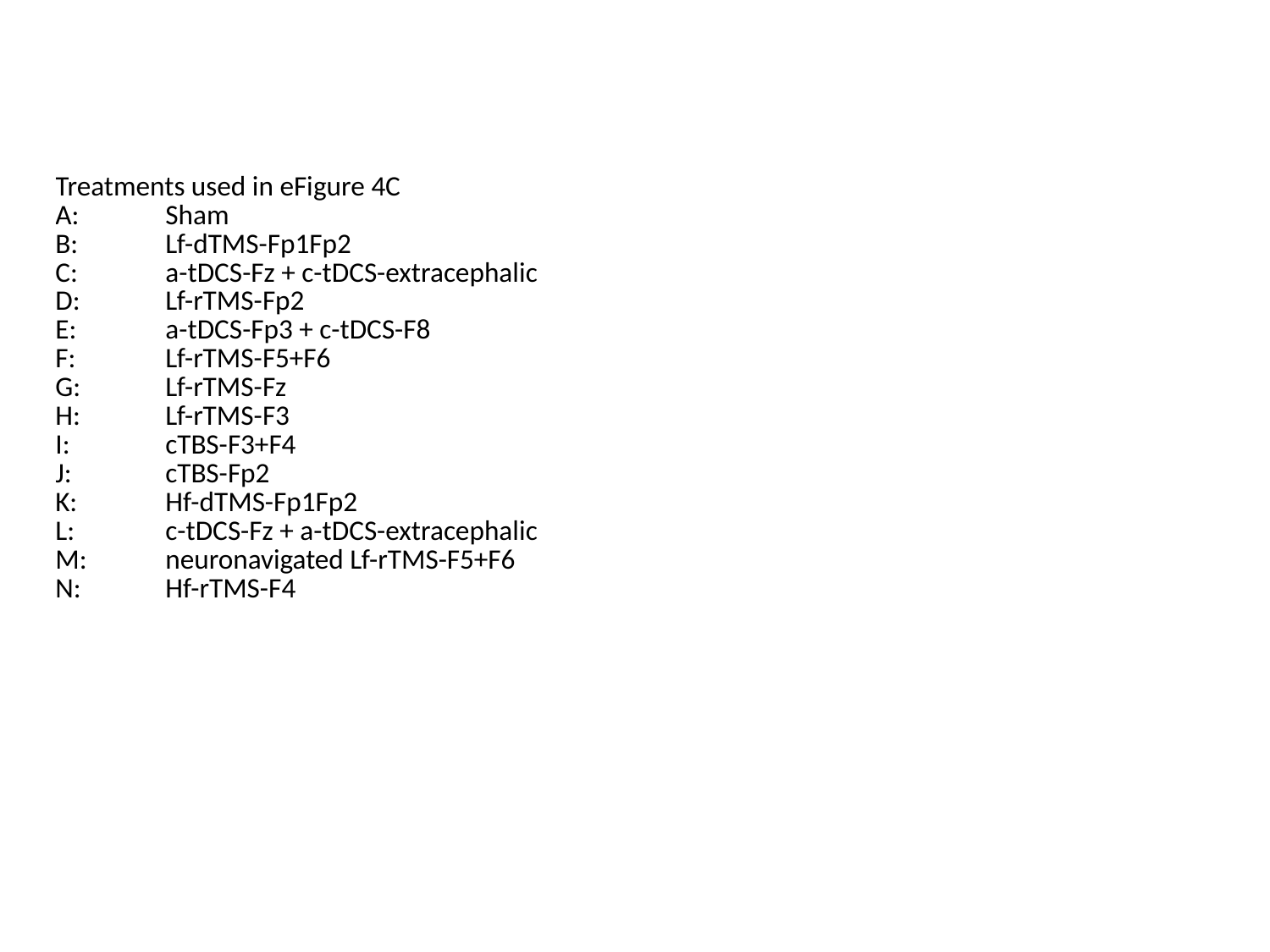

| Treatments used in eFigure 4C | |
| --- | --- |
| A: | Sham |
| B: | Lf-dTMS-Fp1Fp2 |
| C: | a-tDCS-Fz + c-tDCS-extracephalic |
| D: | Lf-rTMS-Fp2 |
| E: | a-tDCS-Fp3 + c-tDCS-F8 |
| F: | Lf-rTMS-F5+F6 |
| G: | Lf-rTMS-Fz |
| H: | Lf-rTMS-F3 |
| I: | cTBS-F3+F4 |
| J: | cTBS-Fp2 |
| K: | Hf-dTMS-Fp1Fp2 |
| L: | c-tDCS-Fz + a-tDCS-extracephalic |
| M: | neuronavigated Lf-rTMS-F5+F6 |
| N: | Hf-rTMS-F4 |

## Slide 21
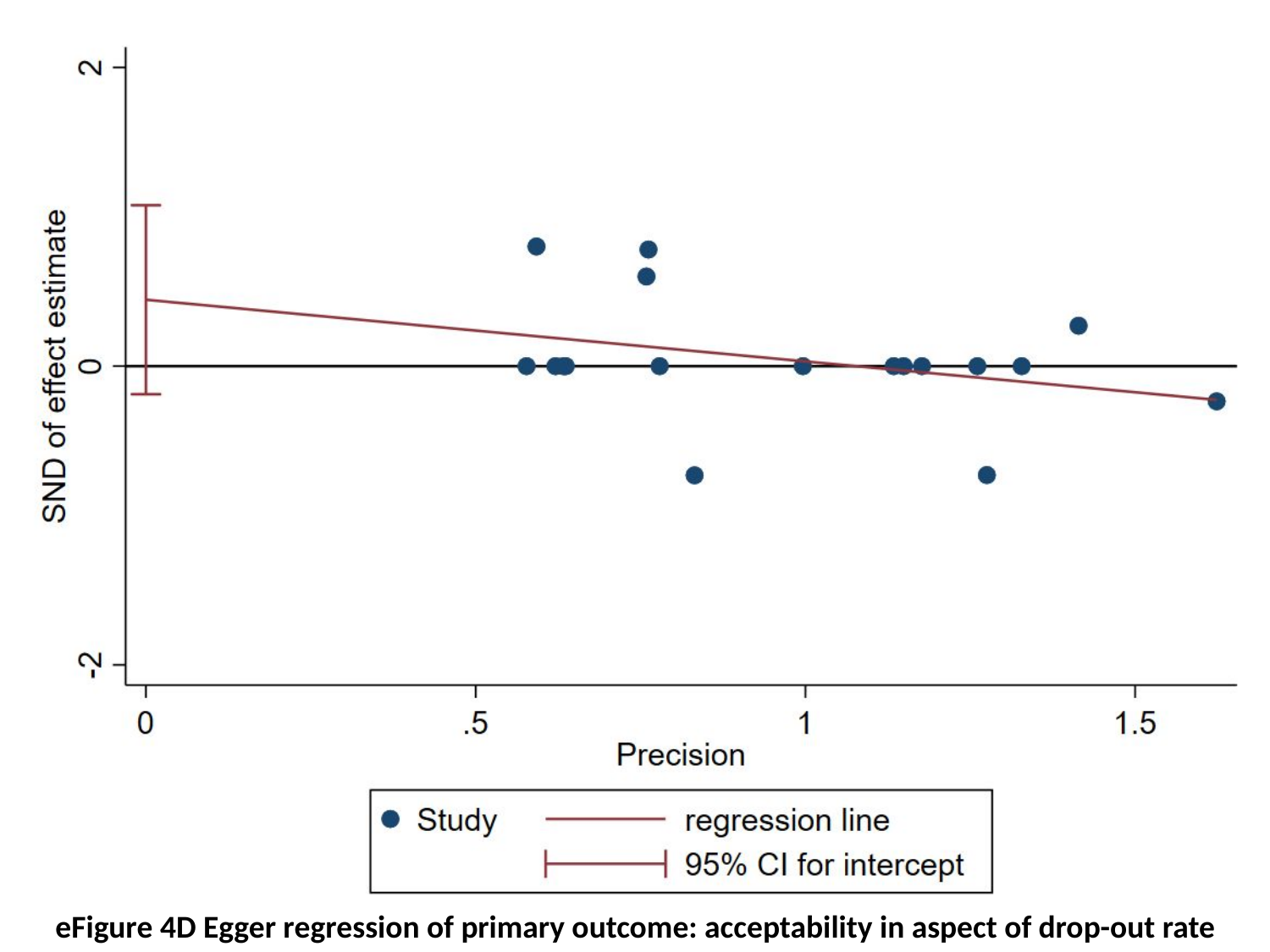

eFigure 4D Egger regression of primary outcome: acceptability in aspect of drop-out rate

## Slide 22
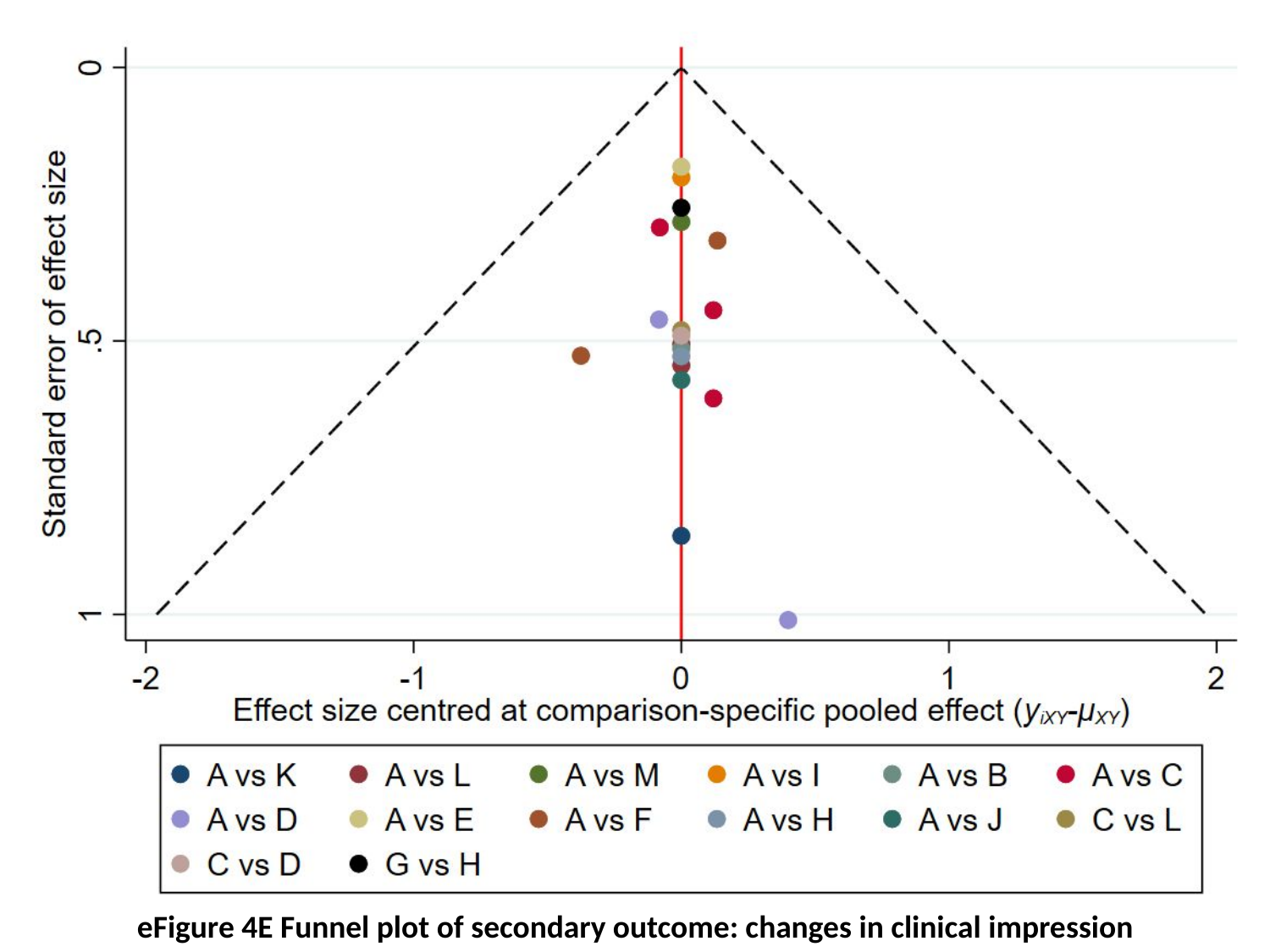

eFigure 4E Funnel plot of secondary outcome: changes in clinical impression

## Slide 23
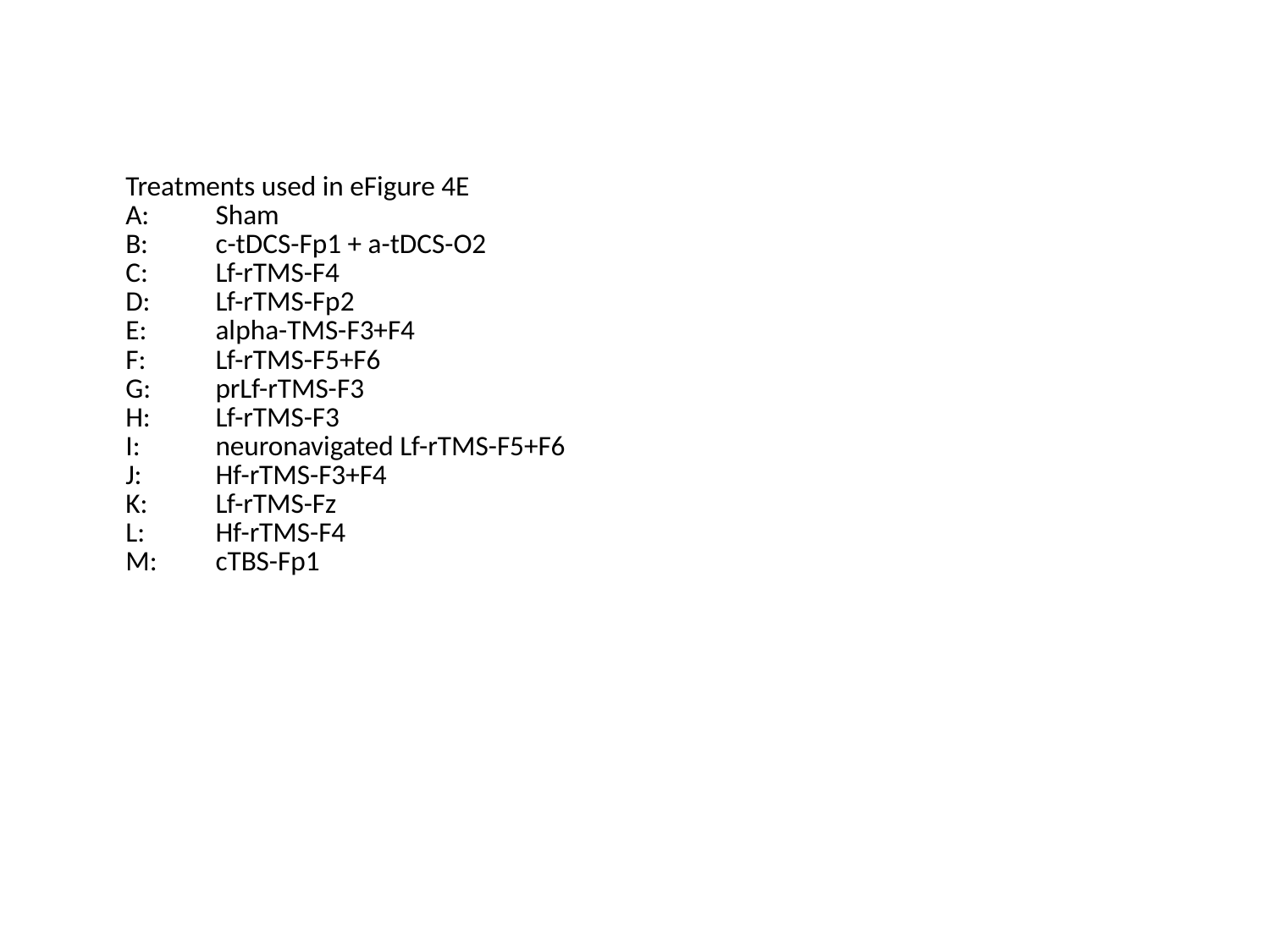

| Treatments used in eFigure 4E | |
| --- | --- |
| A: | Sham |
| B: | c-tDCS-Fp1 + a-tDCS-O2 |
| C: | Lf-rTMS-F4 |
| D: | Lf-rTMS-Fp2 |
| E: | alpha-TMS-F3+F4 |
| F: | Lf-rTMS-F5+F6 |
| G: | prLf-rTMS-F3 |
| H: | Lf-rTMS-F3 |
| I: | neuronavigated Lf-rTMS-F5+F6 |
| J: | Hf-rTMS-F3+F4 |
| K: | Lf-rTMS-Fz |
| L: | Hf-rTMS-F4 |
| M: | cTBS-Fp1 |

## Slide 24
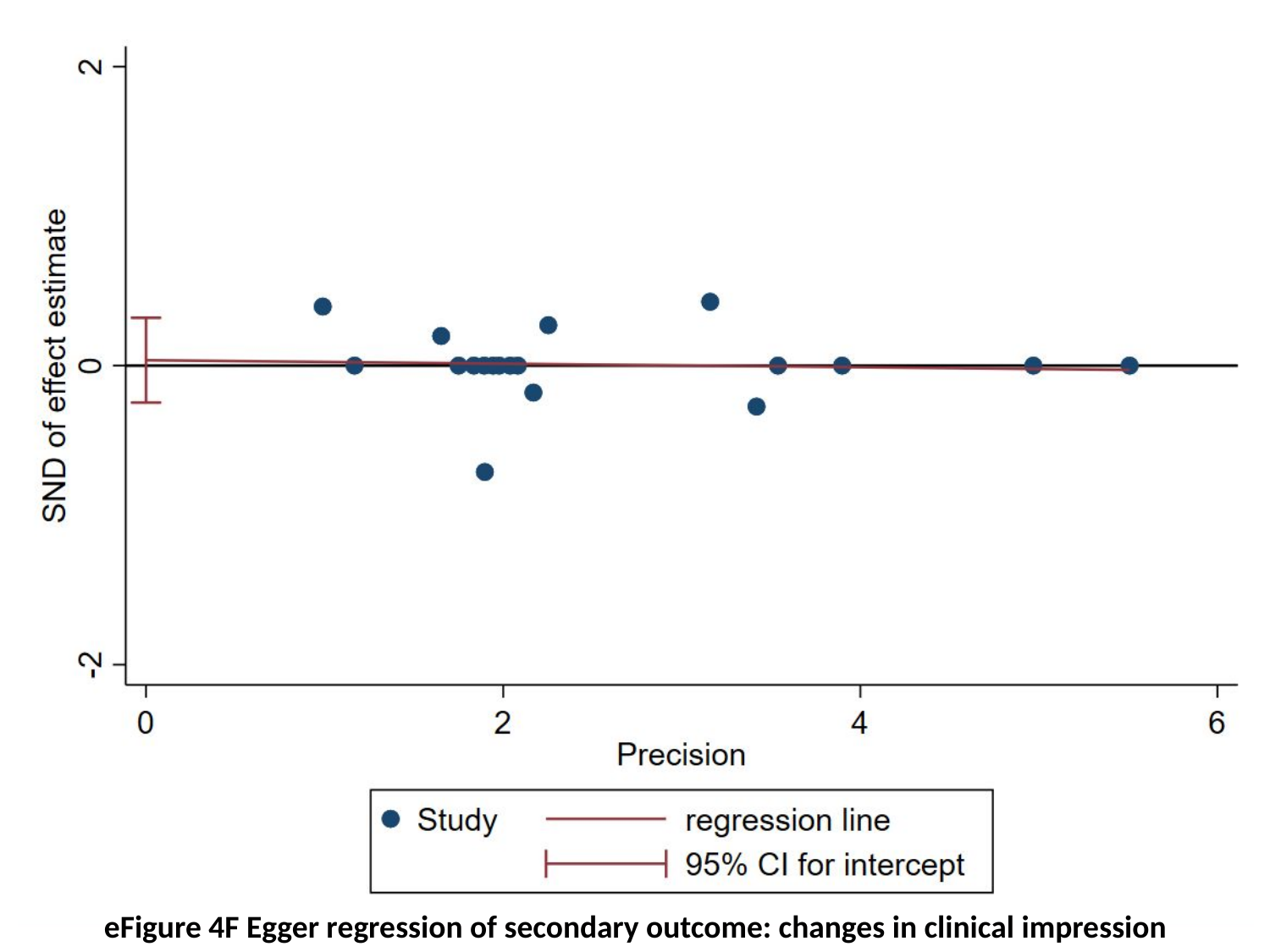

eFigure 4F Egger regression of secondary outcome: changes in clinical impression

## Slide 25
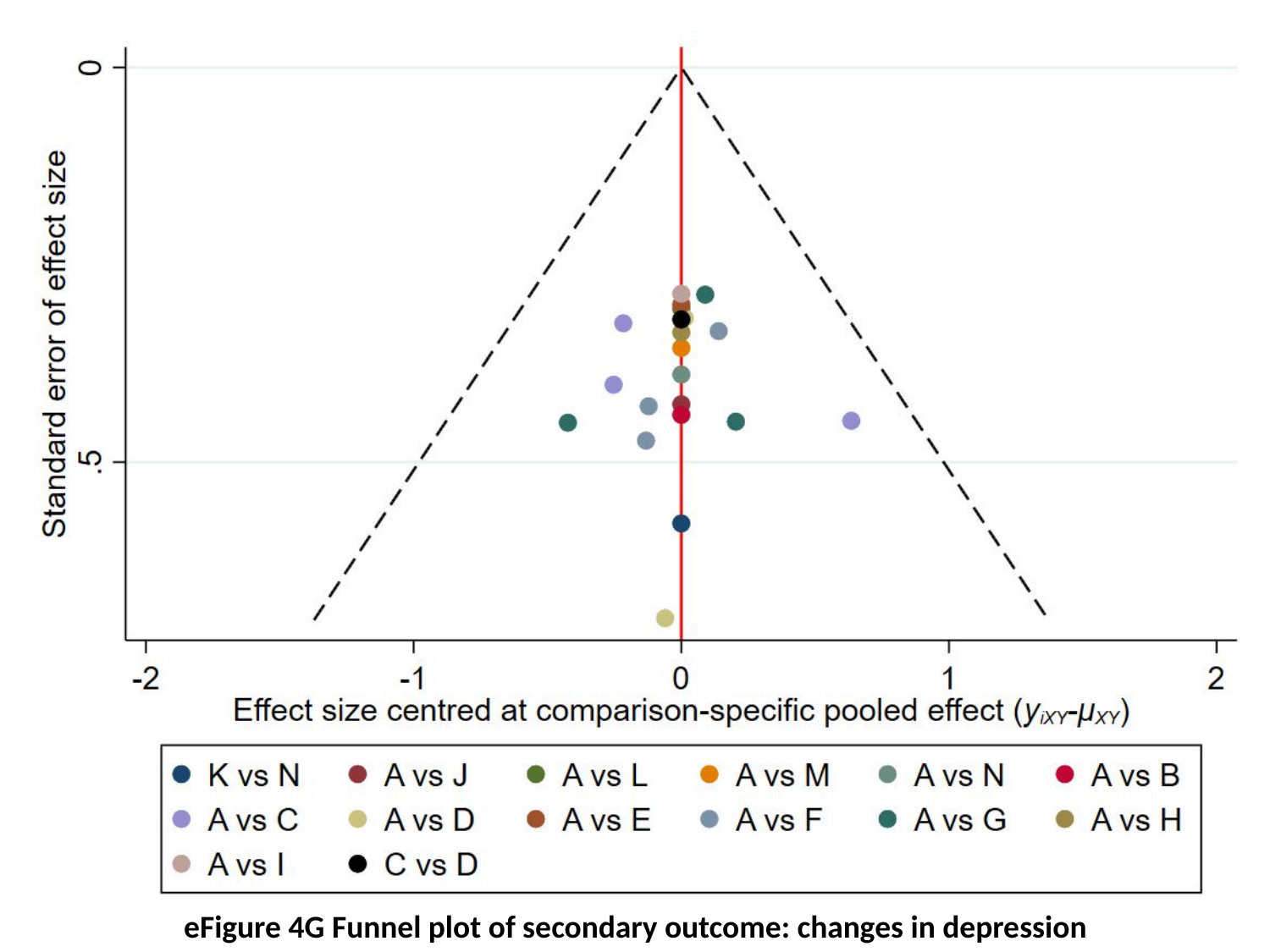

eFigure 4G Funnel plot of secondary outcome: changes in depression

## Slide 26
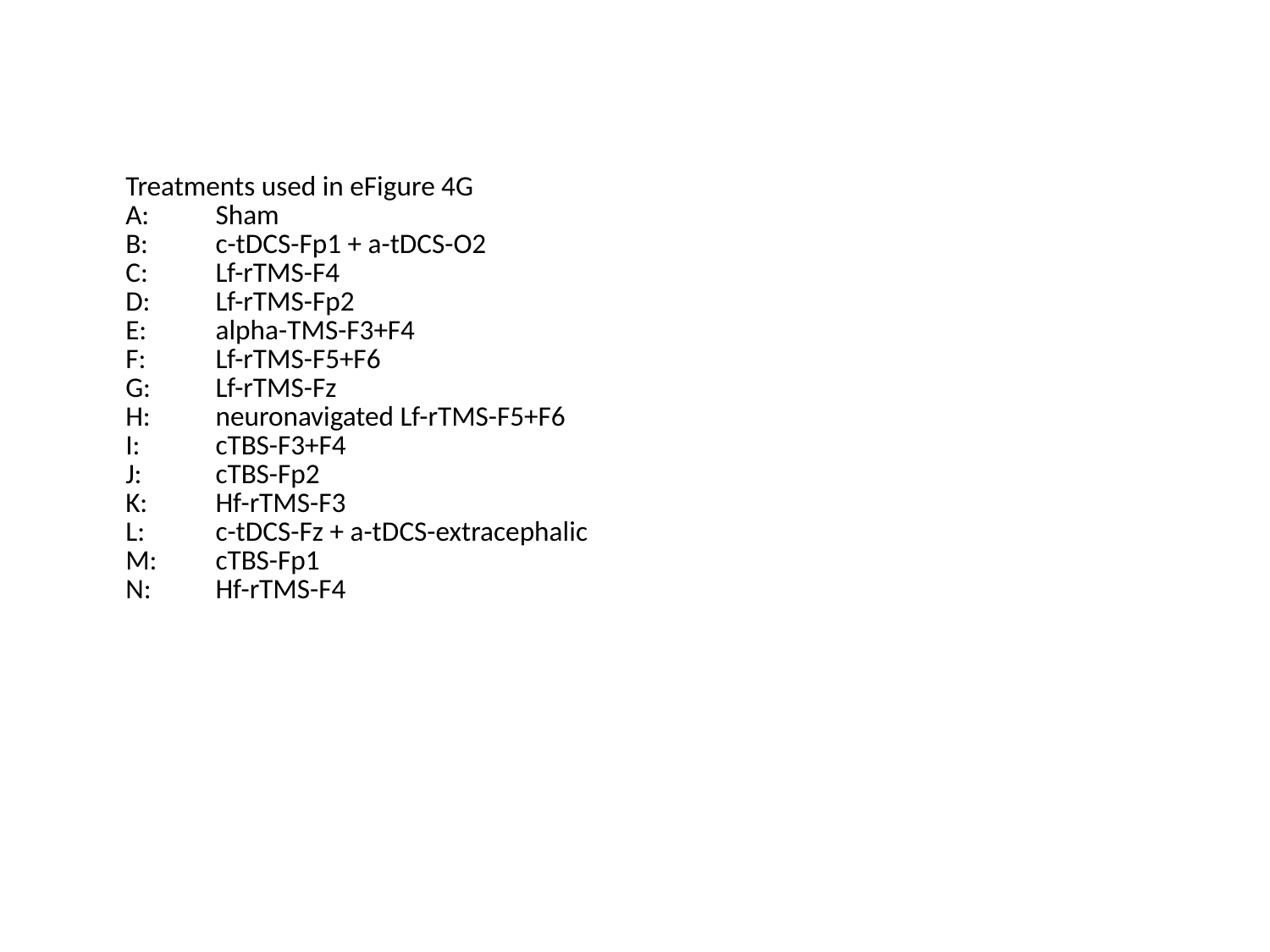

| Treatments used in eFigure 4G | |
| --- | --- |
| A: | Sham |
| B: | c-tDCS-Fp1 + a-tDCS-O2 |
| C: | Lf-rTMS-F4 |
| D: | Lf-rTMS-Fp2 |
| E: | alpha-TMS-F3+F4 |
| F: | Lf-rTMS-F5+F6 |
| G: | Lf-rTMS-Fz |
| H: | neuronavigated Lf-rTMS-F5+F6 |
| I: | cTBS-F3+F4 |
| J: | cTBS-Fp2 |
| K: | Hf-rTMS-F3 |
| L: | c-tDCS-Fz + a-tDCS-extracephalic |
| M: | cTBS-Fp1 |
| N: | Hf-rTMS-F4 |

## Slide 27
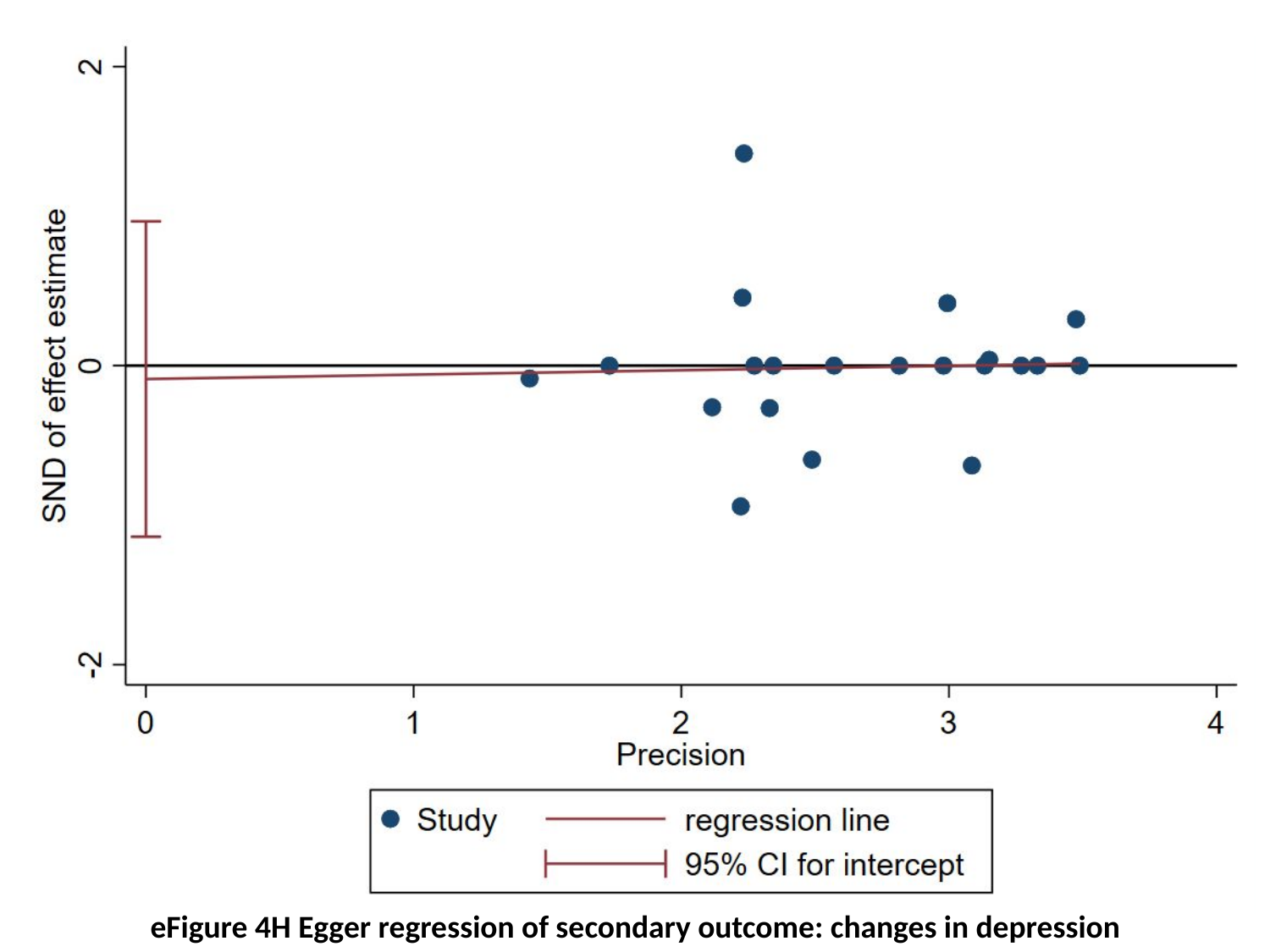

eFigure 4H Egger regression of secondary outcome: changes in depression

## Slide 28
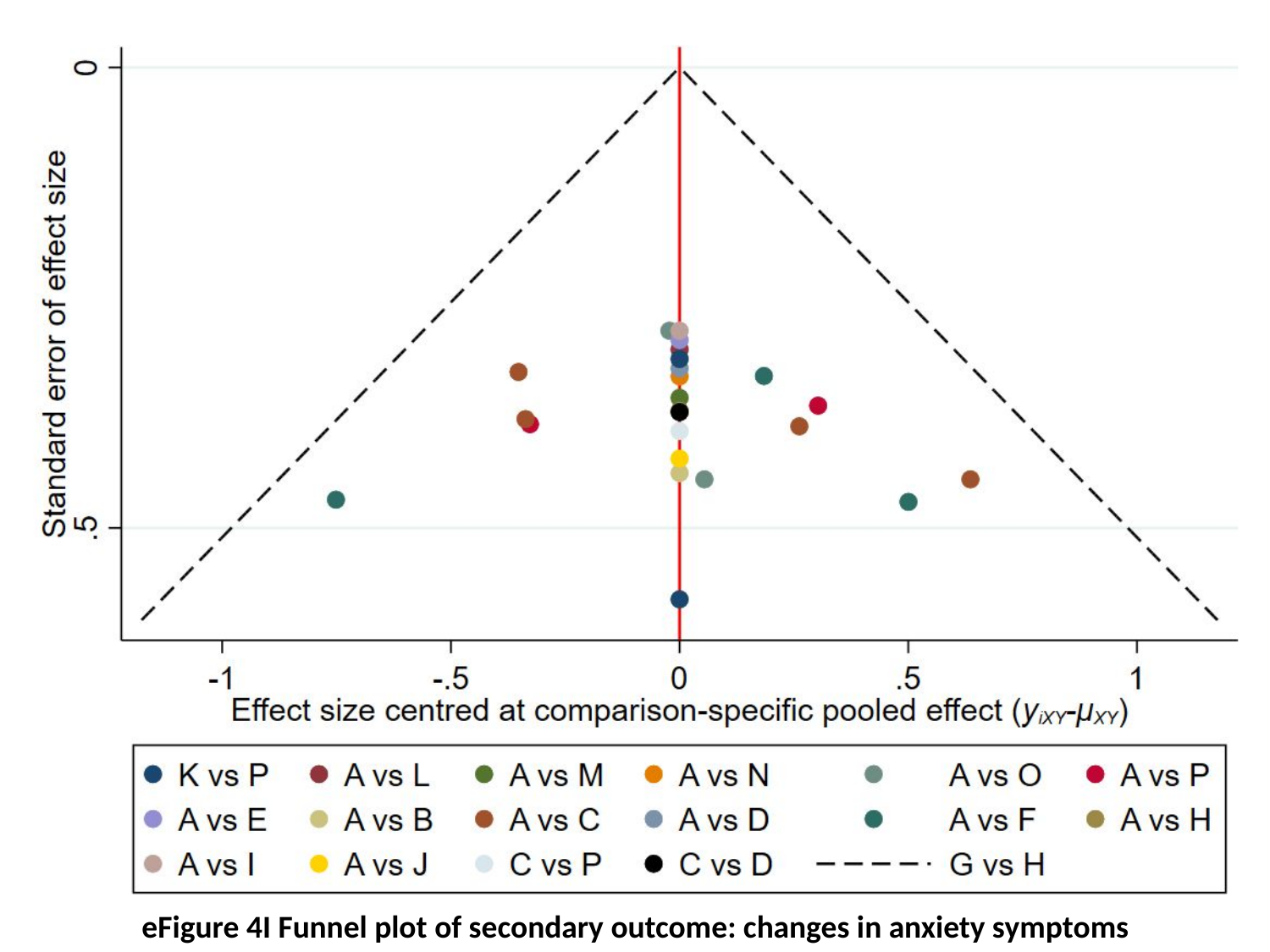

eFigure 4I Funnel plot of secondary outcome: changes in anxiety symptoms

## Slide 29
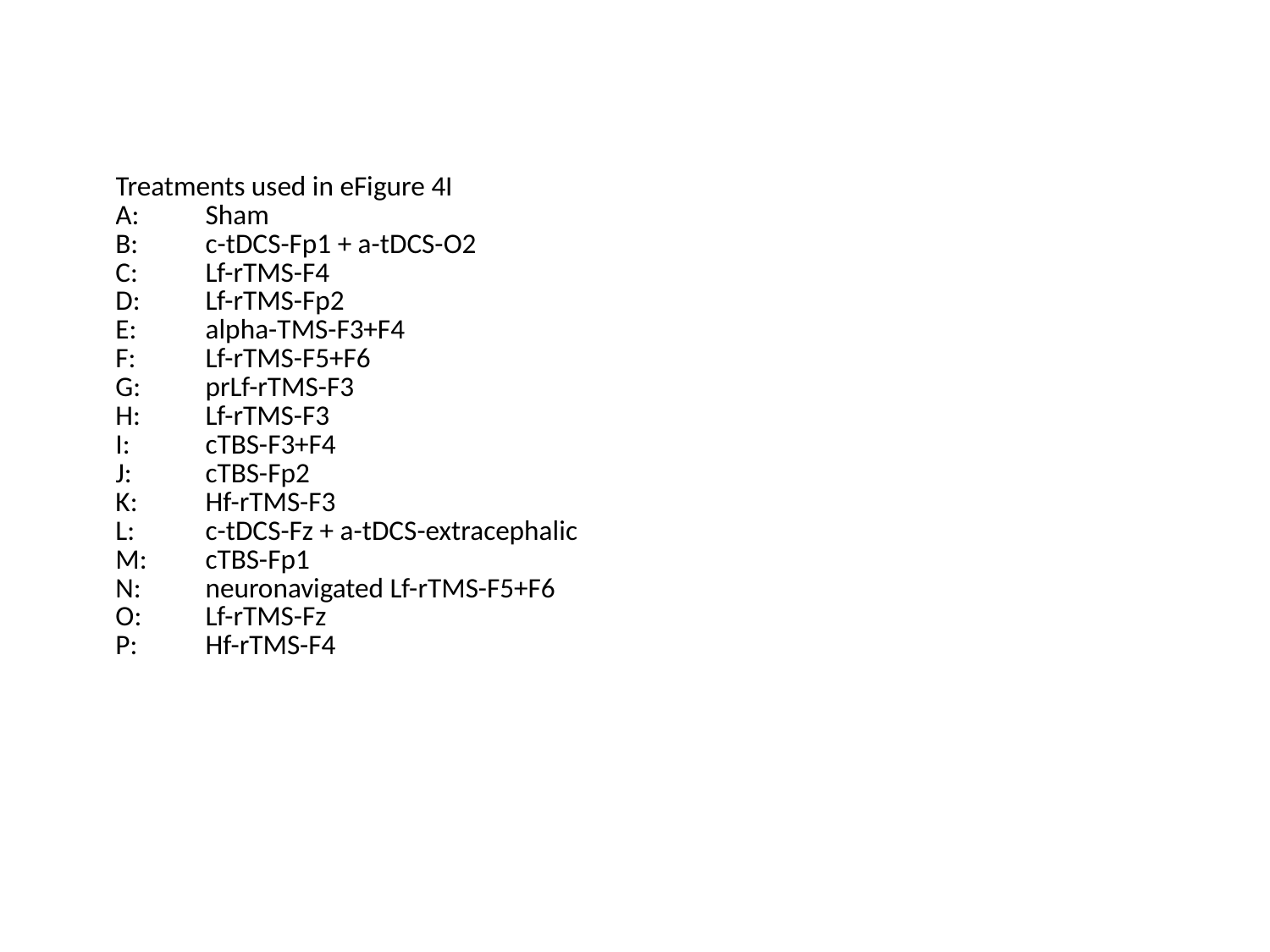

| Treatments used in eFigure 4I | |
| --- | --- |
| A: | Sham |
| B: | c-tDCS-Fp1 + a-tDCS-O2 |
| C: | Lf-rTMS-F4 |
| D: | Lf-rTMS-Fp2 |
| E: | alpha-TMS-F3+F4 |
| F: | Lf-rTMS-F5+F6 |
| G: | prLf-rTMS-F3 |
| H: | Lf-rTMS-F3 |
| I: | cTBS-F3+F4 |
| J: | cTBS-Fp2 |
| K: | Hf-rTMS-F3 |
| L: | c-tDCS-Fz + a-tDCS-extracephalic |
| M: | cTBS-Fp1 |
| N: | neuronavigated Lf-rTMS-F5+F6 |
| O: | Lf-rTMS-Fz |
| P: | Hf-rTMS-F4 |

## Slide 30
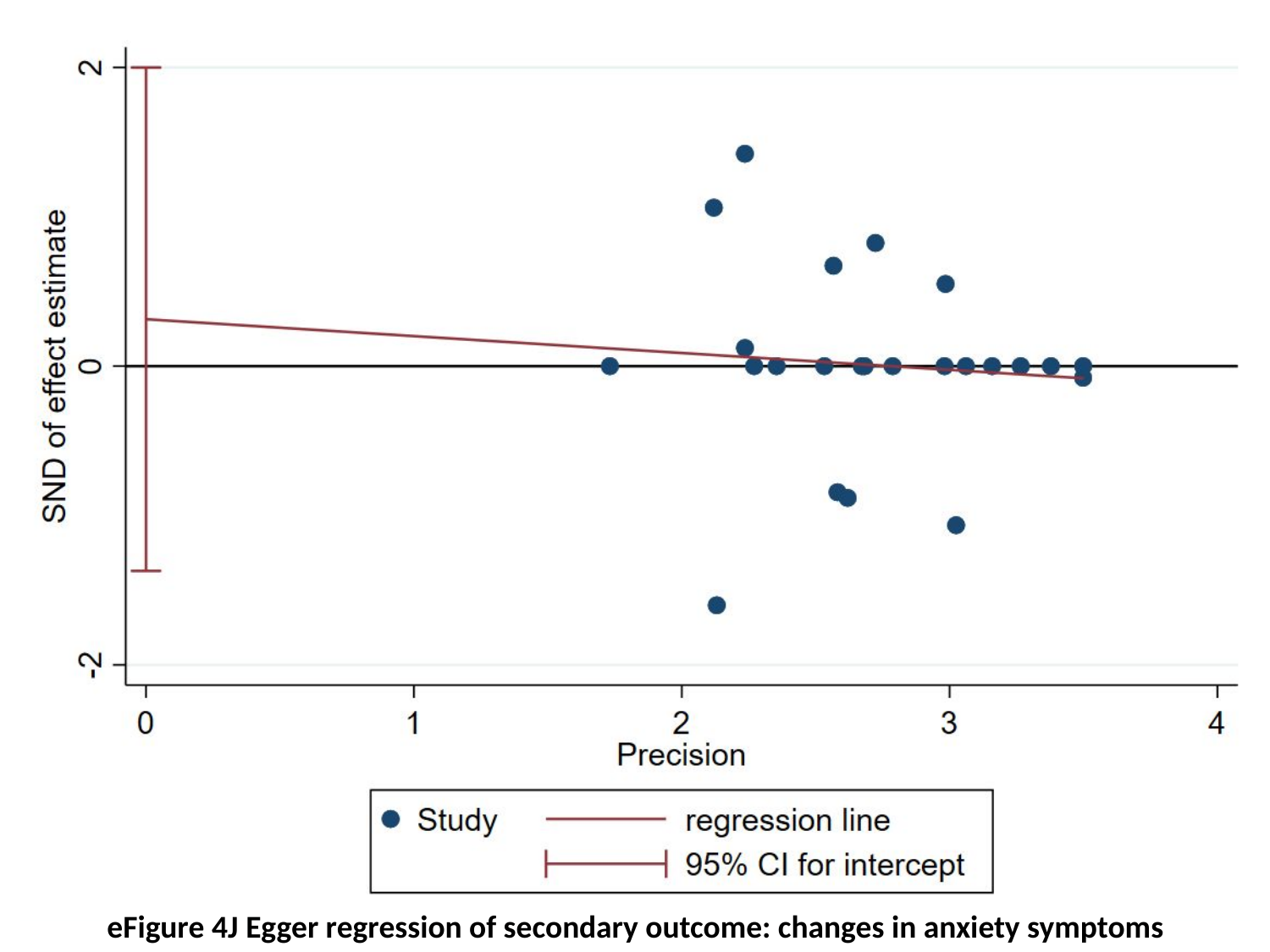

eFigure 4J Egger regression of secondary outcome: changes in anxiety symptoms

## Slide 31
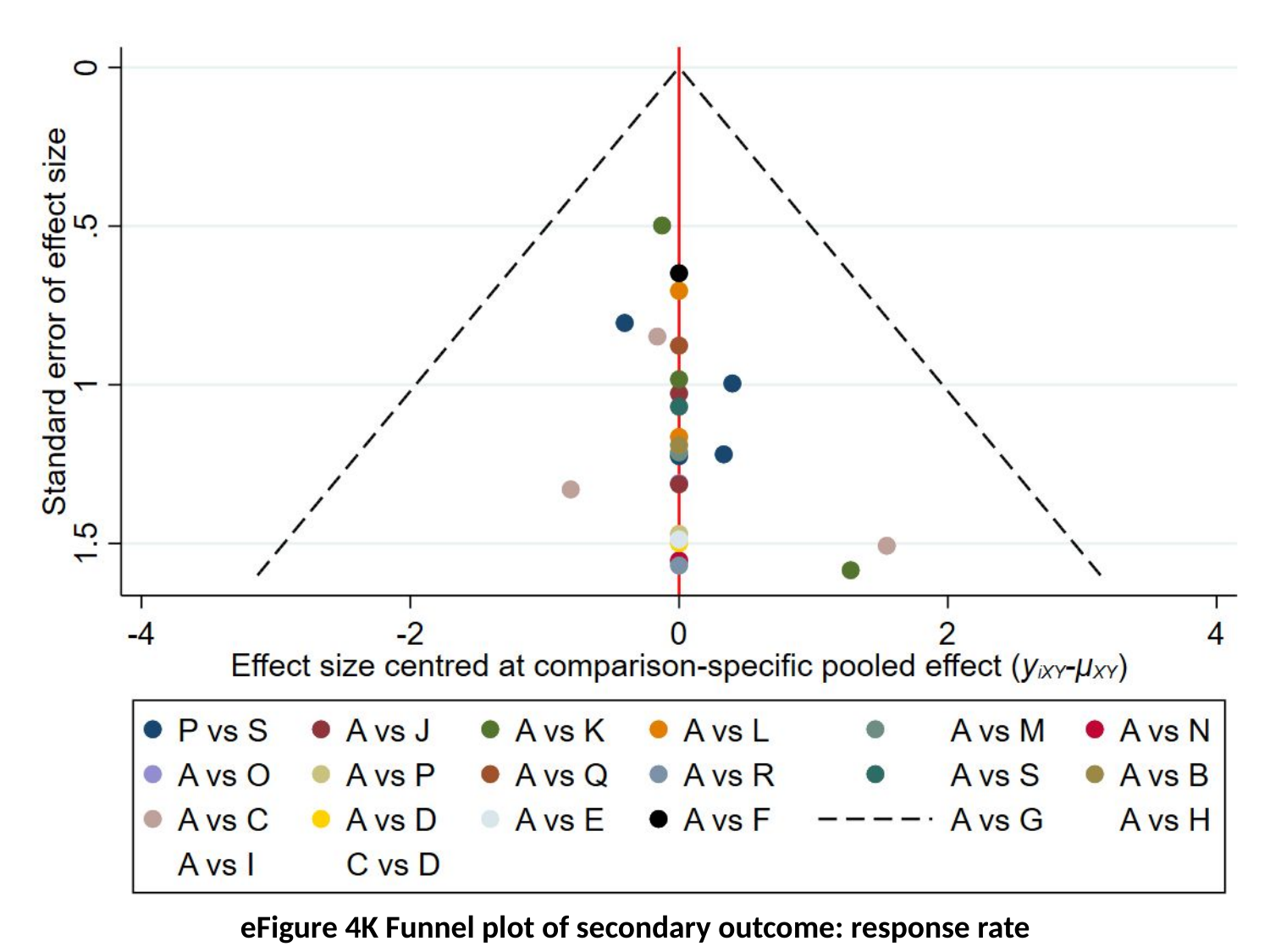

eFigure 4K Funnel plot of secondary outcome: response rate

## Slide 32
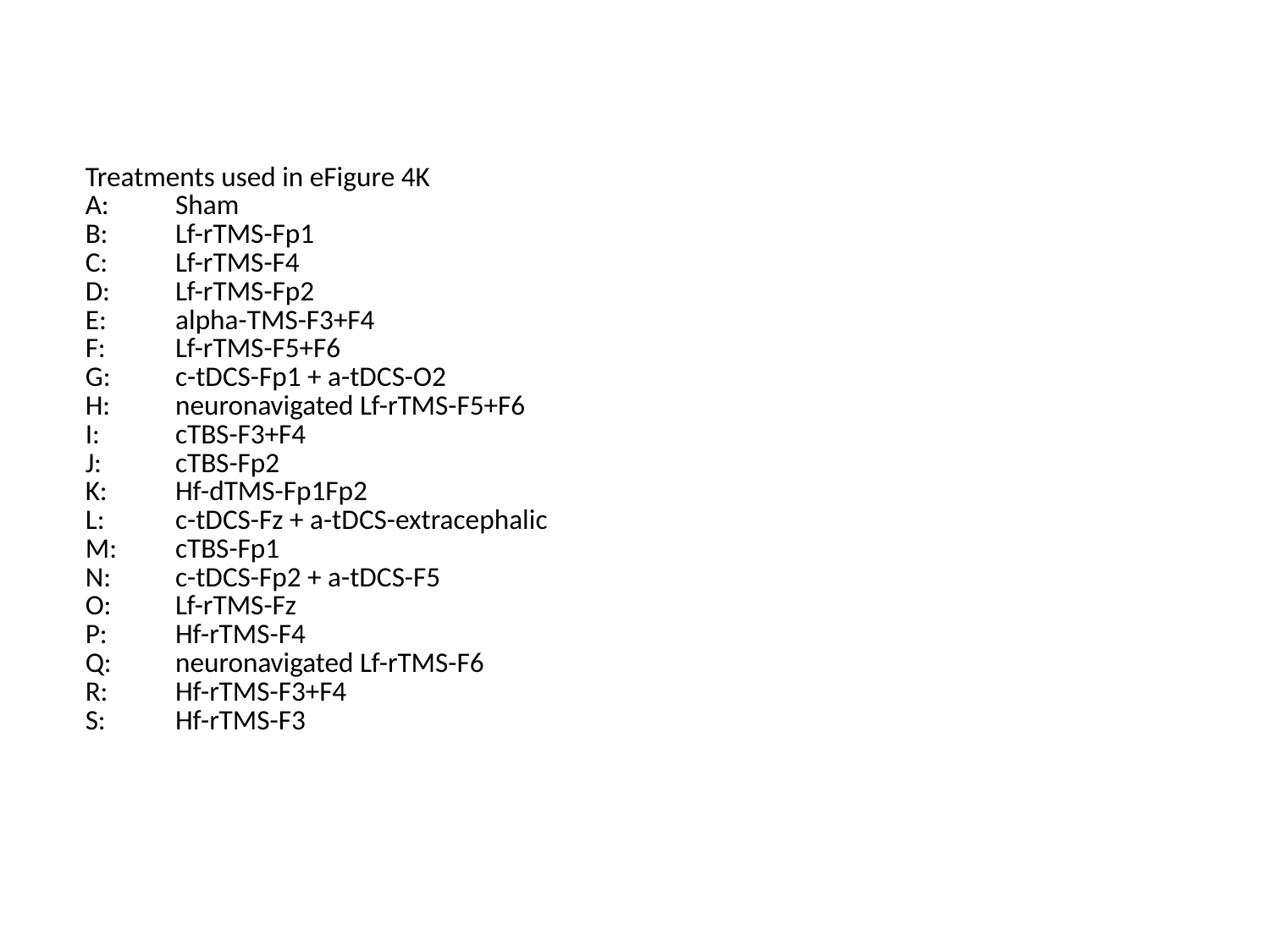

| Treatments used in eFigure 4K | |
| --- | --- |
| A: | Sham |
| B: | Lf-rTMS-Fp1 |
| C: | Lf-rTMS-F4 |
| D: | Lf-rTMS-Fp2 |
| E: | alpha-TMS-F3+F4 |
| F: | Lf-rTMS-F5+F6 |
| G: | c-tDCS-Fp1 + a-tDCS-O2 |
| H: | neuronavigated Lf-rTMS-F5+F6 |
| I: | cTBS-F3+F4 |
| J: | cTBS-Fp2 |
| K: | Hf-dTMS-Fp1Fp2 |
| L: | c-tDCS-Fz + a-tDCS-extracephalic |
| M: | cTBS-Fp1 |
| N: | c-tDCS-Fp2 + a-tDCS-F5 |
| O: | Lf-rTMS-Fz |
| P: | Hf-rTMS-F4 |
| Q: | neuronavigated Lf-rTMS-F6 |
| R: | Hf-rTMS-F3+F4 |
| S: | Hf-rTMS-F3 |

## Slide 33
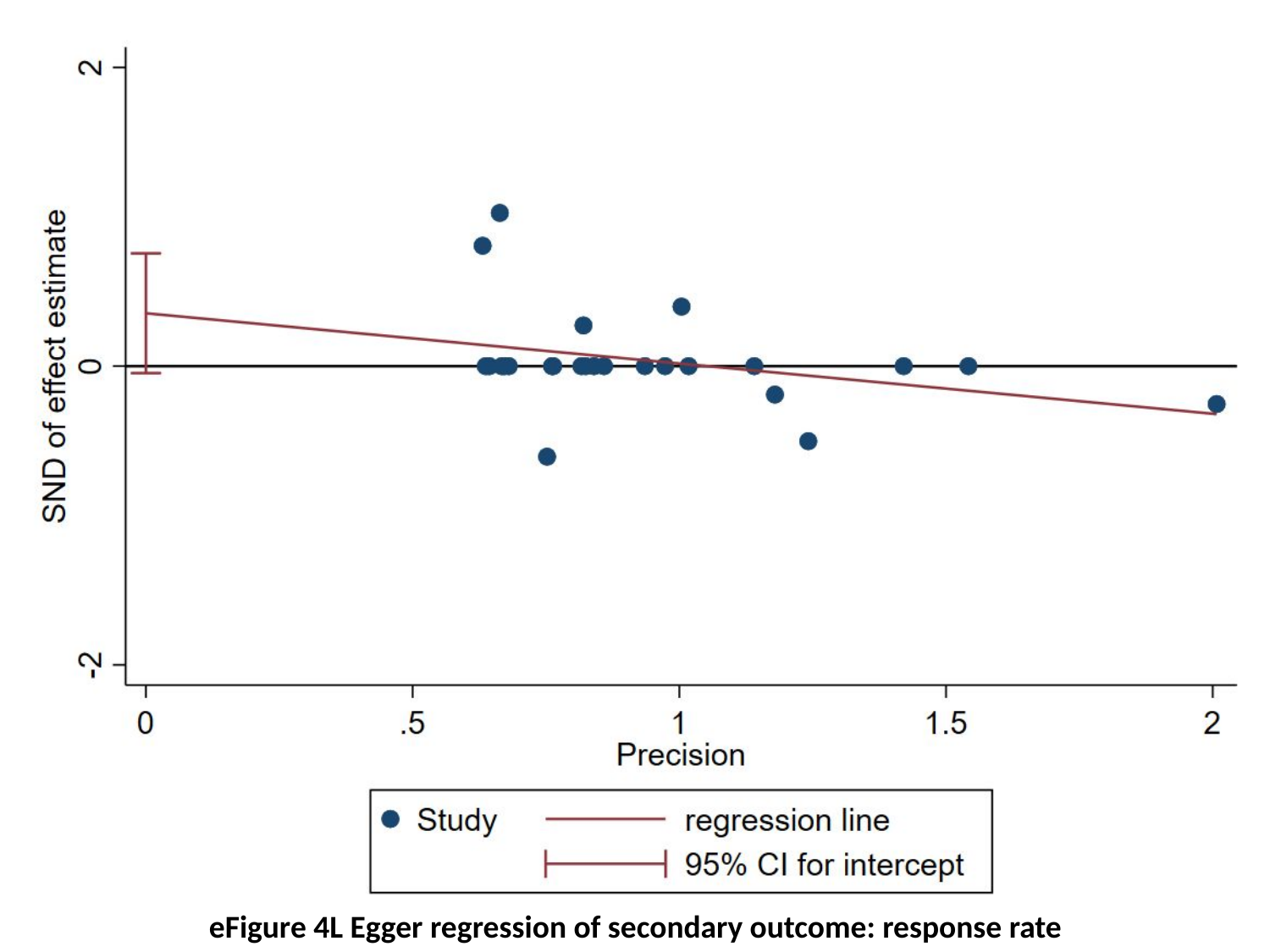

eFigure 4L Egger regression of secondary outcome: response rate

## Slide 34
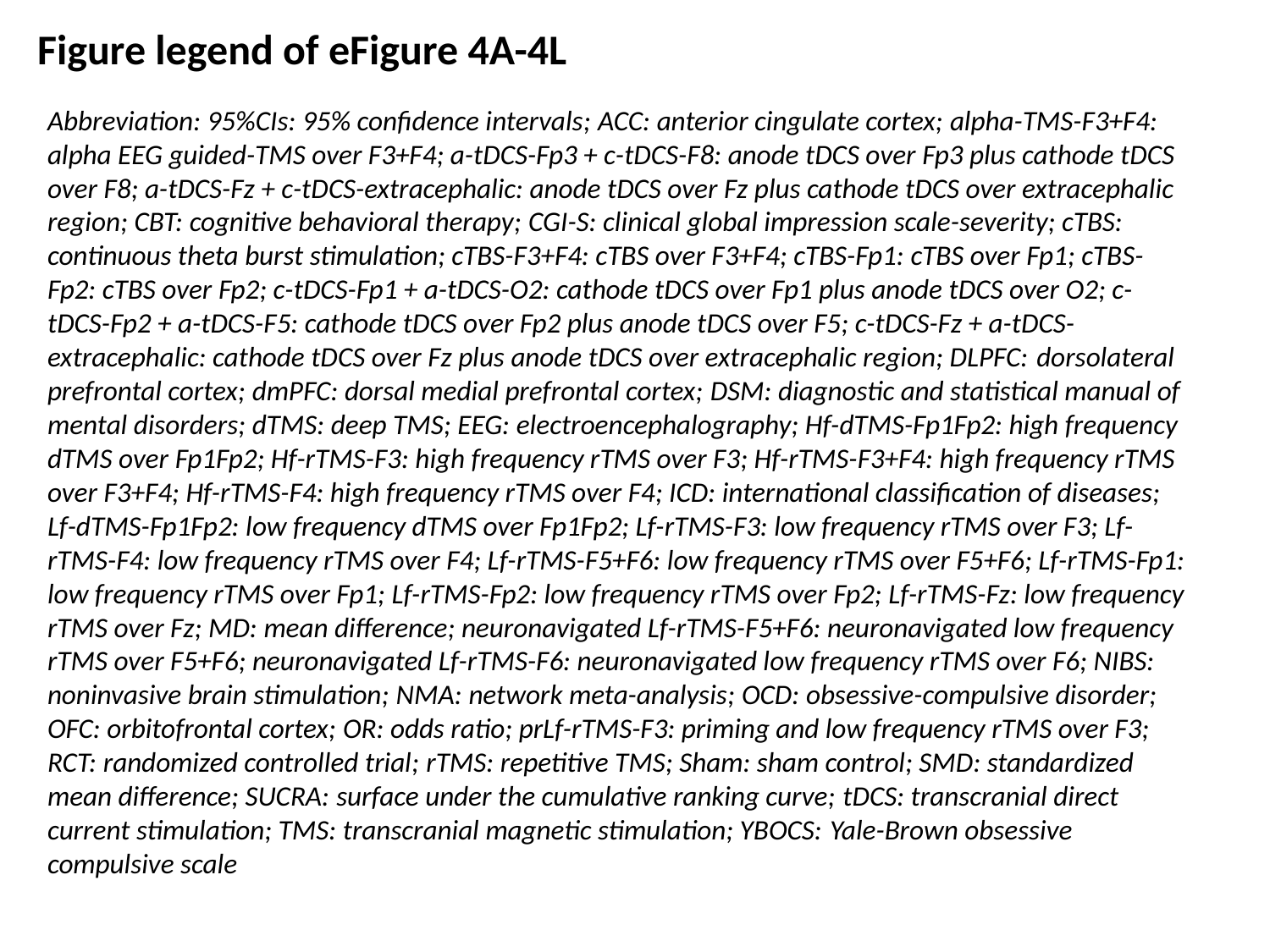

Figure legend of eFigure 4A-4L
Abbreviation: 95%CIs: 95% confidence intervals; ACC: anterior cingulate cortex; alpha-TMS-F3+F4: alpha EEG guided-TMS over F3+F4; a-tDCS-Fp3 + c-tDCS-F8: anode tDCS over Fp3 plus cathode tDCS over F8; a-tDCS-Fz + c-tDCS-extracephalic: anode tDCS over Fz plus cathode tDCS over extracephalic region; CBT: cognitive behavioral therapy; CGI-S: clinical global impression scale-severity; cTBS: continuous theta burst stimulation; cTBS-F3+F4: cTBS over F3+F4; cTBS-Fp1: cTBS over Fp1; cTBS-Fp2: cTBS over Fp2; c-tDCS-Fp1 + a-tDCS-O2: cathode tDCS over Fp1 plus anode tDCS over O2; c-tDCS-Fp2 + a-tDCS-F5: cathode tDCS over Fp2 plus anode tDCS over F5; c-tDCS-Fz + a-tDCS-extracephalic: cathode tDCS over Fz plus anode tDCS over extracephalic region; DLPFC: dorsolateral prefrontal cortex; dmPFC: dorsal medial prefrontal cortex; DSM: diagnostic and statistical manual of mental disorders; dTMS: deep TMS; EEG: electroencephalography; Hf-dTMS-Fp1Fp2: high frequency dTMS over Fp1Fp2; Hf-rTMS-F3: high frequency rTMS over F3; Hf-rTMS-F3+F4: high frequency rTMS over F3+F4; Hf-rTMS-F4: high frequency rTMS over F4; ICD: international classification of diseases; Lf-dTMS-Fp1Fp2: low frequency dTMS over Fp1Fp2; Lf-rTMS-F3: low frequency rTMS over F3; Lf-rTMS-F4: low frequency rTMS over F4; Lf-rTMS-F5+F6: low frequency rTMS over F5+F6; Lf-rTMS-Fp1: low frequency rTMS over Fp1; Lf-rTMS-Fp2: low frequency rTMS over Fp2; Lf-rTMS-Fz: low frequency rTMS over Fz; MD: mean difference; neuronavigated Lf-rTMS-F5+F6: neuronavigated low frequency rTMS over F5+F6; neuronavigated Lf-rTMS-F6: neuronavigated low frequency rTMS over F6; NIBS: noninvasive brain stimulation; NMA: network meta-analysis; OCD: obsessive-compulsive disorder; OFC: orbitofrontal cortex; OR: odds ratio; prLf-rTMS-F3: priming and low frequency rTMS over F3; RCT: randomized controlled trial; rTMS: repetitive TMS; Sham: sham control; SMD: standardized mean difference; SUCRA: surface under the cumulative ranking curve; tDCS: transcranial direct current stimulation; TMS: transcranial magnetic stimulation; YBOCS: Yale-Brown obsessive compulsive scale
