# Supplementary material for: The Efficacy and Acceptability of Non‐Invasive Brain Stimulation Interventions for Obsessive‐Compulsive Disorder Management: A Network Meta‐Analysis Based on 24 Stimulation Methods
Source: Acta Psychiatr Scand. 2025 Mar 31;152(2):112–24. doi: 10.1111/acps.13809 (PMC12213009; doi:10.1111/acps.13809)
Supplement: Supplementary file 2 — Tables S1–S10. [file ACPS-152-112-s001.docx]

**eTable 1: PRISMA 2020 checklist**

| **Section and Topic** | **Item #** | **Checklist item** | **Page where item is reported** |
| --- | --- | --- | --- |
| **TITLE** | | |  |
| Title | 1 | Identify the report as a systematic review. | 1 |
| **ABSTRACT** | | |  |
| Abstract | 2 | See the PRISMA 2020 for Abstracts checklist. | 3-4 |
| **INTRODUCTION** | | |  |
| Rationale | 3 | Describe the rationale for the review in the context of existing knowledge. | 5-6 |
| Objectives | 4 | Provide an explicit statement of the objective(s) or question(s) the review addresses. | 6-7 |
| **METHODS** | | |  |
| Eligibility criteria | 5 | Specify the inclusion and exclusion criteria for the review and how studies were grouped for the syntheses. | 8-9 |
| Information sources | 6 | Specify all databases, registers, websites, organisations, reference lists and other sources searched or consulted to identify studies. Specify the date when each source was last searched or consulted. | 8-9 |
| Search strategy | 7 | Present the full search strategies for all databases, registers and websites, including any filters and limits used. | 8-9 |
| Selection process | 8 | Specify the methods used to decide whether a study met the inclusion criteria of the review, including how many reviewers screened each record and each report retrieved, whether they worked independently, and if applicable, details of automation tools used in the process. | 8-9 |
| Data collection process | 9 | Specify the methods used to collect data from reports, including how many reviewers collected data from each report, whether they worked independently, any processes for obtaining or confirming data from study investigators, and if applicable, details of automation tools used in the process. | 8-9 |
| Data items | 10a | List and define all outcomes for which data were sought. Specify whether all results that were compatible with each outcome domain in each study were sought (e.g. for all measures, time points, analyses), and if not, the methods used to decide which results to collect. | 9-10 |
|  | 10b | List and define all other variables for which data were sought (e.g. participant and intervention characteristics, funding sources). Describe any assumptions made about any missing or unclear information. | 9-10 |
| Study risk of bias assessment | 11 | Specify the methods used to assess risk of bias in the included studies, including details of the tool(s) used, how many reviewers assessed each study and whether they worked independently, and if applicable, details of automation tools used in the process. | 9-10 |
| Effect measures | 12 | Specify for each outcome the effect measure(s) (e.g. risk ratio, mean difference) used in the synthesis or presentation of results. | 9-10 |
| Synthesis methods | 13a | Describe the processes used to decide which studies were eligible for each synthesis (e.g. tabulating the study intervention characteristics and comparing against the planned groups for each synthesis (item #5)). | 9-10 |
|  | 13b | Describe any methods required to prepare the data for presentation or synthesis, such as handling of missing summary statistics, or data conversions. | 9-10 |
|  | 13c | Describe any methods used to tabulate or visually display results of individual studies and syntheses. | 10-13 |
|  | 13d | Describe any methods used to synthesize results and provide a rationale for the choice(s). If meta-analysis was performed, describe the model(s), method(s) to identify the presence and extent of statistical heterogeneity, and software package(s) used. | 10-13 |
|  | 13e | Describe any methods used to explore possible causes of heterogeneity among study results (e.g. subgroup analysis, meta-regression). | 10-13 |
|  | 13f | Describe any sensitivity analyses conducted to assess robustness of the synthesized results. | 10-13 |
| Reporting bias assessment | 14 | Describe any methods used to assess risk of bias due to missing results in a synthesis (arising from reporting biases). | 10-13 |
| Certainty assessment | 15 | Describe any methods used to assess certainty (or confidence) in the body of evidence for an outcome. | 10-13 |
| **RESULTS** | | |  |
| Study selection | 16a | Describe the results of the search and selection process, from the number of records identified in the search to the number of studies included in the review, ideally using a flow diagram. | 14-15, Fig 1 |
|  | 16b | Cite studies that might appear to meet the inclusion criteria, but which were excluded, and explain why they were excluded. | 14-15, eTab 2 |
| Study characteristics | 17 | Cite each included study and present its characteristics. | 14-15, eTab 4 |
| Risk of bias in studies | 18 | Present assessments of risk of bias for each included study. | 14-15, eFig 2 |
| Results of individual studies | 19 | For all outcomes, present, for each study: (a) summary statistics for each group (where appropriate) and (b) an effect estimate and its precision (e.g. confidence/credible interval), ideally using structured tables or plots. | 14-15, eTab 4 |
| Results of syntheses | 20a | For each synthesis, briefly summarise the characteristics and risk of bias among contributing studies. | 14-15, eFig 3 |
|  | 20b | Present results of all statistical syntheses conducted. If meta-analysis was done, present for each the summary estimate and its precision (e.g. confidence/credible interval) and measures of statistical heterogeneity. If comparing groups, describe the direction of the effect. | 14-15, Fig 3, eFig 3 |
|  | 20c | Present results of all investigations of possible causes of heterogeneity among study results. | 14-15, eTab 8-10 |
|  | 20d | Present results of all sensitivity analyses conducted to assess the robustness of the synthesized results. | 15-17 |
| Reporting biases | 21 | Present assessments of risk of bias due to missing results (arising from reporting biases) for each synthesis assessed. | 15-17, eFig 3 |
| Certainty of evidence | 22 | Present assessments of certainty (or confidence) in the body of evidence for each outcome assessed. | 15-17, eTab 8-11 |
| **DISCUSSION** | | |  |
| Discussion | 23a | Provide a general interpretation of the results in the context of other evidence. | 18-20 |
|  | 23b | Discuss any limitations of the evidence included in the review. | 20-21 |
|  | 23c | Discuss any limitations of the review processes used. | 20-21 |
|  | 23d | Discuss implications of the results for practice, policy, and future research. | 22 |
| **OTHER INFORMATION** | | |  |
| Registration and protocol | 24a | Provide registration information for the review, including register name and registration number, or state that the review was not registered. | 8 |
|  | 24b | Indicate where the review protocol can be accessed, or state that a protocol was not prepared. | 8 |
|  | 24c | Describe and explain any amendments to information provided at registration or in the protocol. | 8 |
| Support | 25 | Describe sources of financial or non-financial support for the review, and the role of the funders or sponsors in the review. | 23 |
| Competing interests | 26 | Declare any competing interests of review authors. | 23 |
| Availability of data, code and other materials | 27 | Report which of the following are publicly available and where they can be found: template data collection forms; data extracted from included studies; data used for all analyses; analytic code; any other materials used in the review. | 23 |

The current checklist followed the latest PRISMA 2020 guideline.^1^

**eTable 2: the keyword used in each database and search result**

| Database | Keyword | Filter | Date | Result |
| --- | --- | --- | --- | --- |
| PubMed | (deep transcranial magnetic stimulation OR dTMS OR repetitive transcranial magnetic stimulation OR rTMS OR TMS OR non-invasive brain stimulation OR theta burst stimulation OR transcranial direct current stimulation OR TBS OR tDCS OR vagus nerve stimulation OR vagal nerve stimulation OR tVNS OR nVNS OR VNS OR static magnetic field stimulation OR colon electric stimulation) AND (OCD OR obsessive-compulsive disorder OR obsessive compulsive disorder) AND (random OR randomized OR randomised) | NA | 2023/1/27 | 120 |
| Embase | (deep transcranial magnetic stimulation OR dTMS OR repetitive transcranial magnetic stimulation OR rTMS OR TMS OR non-invasive brain stimulation OR theta burst stimulation OR transcranial direct current stimulation OR TBS OR tDCS OR vagus nerve stimulation OR vagal nerve stimulation OR tVNS OR nVNS OR VNS OR static magnetic field stimulation OR colon electric stimulation) AND (OCD OR obsessive-compulsive disorder OR obsessive compulsive disorder) AND (random OR randomized OR randomised) | NA | 2023/1/27 | 304 |
| ClinicalKey | (non-invasive brain stimulation) AND (OCD OR obsessive-compulsive disorder OR obsessive compulsive disorder) | NA | 2023/1/27 | 226 |
| Cochrane CENTRAL | (deep transcranial magnetic stimulation OR dTMS OR repetitive transcranial magnetic stimulation OR rTMS OR TMS OR non-invasive brain stimulation OR theta burst stimulation OR transcranial direct current stimulation OR TBS OR tDCS OR vagus nerve stimulation OR vagal nerve stimulation OR tVNS OR nVNS OR VNS OR static magnetic field stimulation OR colon electric stimulation) AND (OCD OR obsessive-compulsive disorder OR obsessive compulsive disorder) AND (random OR randomized OR randomised) | NA | 2023/1/27 | 211 |
| ProQuest | (non-invasive brain stimulation) AND (OCD OR obsessive-compulsive disorder OR obsessive compulsive disorder) | NA | 2023/1/27 | 1698 |
| ScienceDirect | (non-invasive brain stimulation) AND (OCD OR obsessive-compulsive disorder OR obsessive compulsive disorder) | NA | 2023/1/27 | 2172 |
| Web of Science | (non-invasive brain stimulation) AND (OCD OR obsessive-compulsive disorder OR obsessive compulsive disorder) | NA | 2023/1/27 | 98 |
| ClinicalTrials.gov | (non-invasive brain stimulation) AND (OCD OR obsessive-compulsive disorder OR obsessive compulsive disorder) | NA | 2023/1/27 | 3 |

Abbreviation: NA: not applied

**eTable 3: Excluded studies and reason**

| Reason | Numbers | References |
| --- | --- | --- |
| Clinical guideline | 1 | ^2^ |
| Duplicate sample source with another included study | 2 | ^3,4^ |
| Lack of information about the laterality of the stimulation site | 1 | ^5^ |
| Lack of sufficient data despite of request | 2 | ^6,7^ |
| Meta-analysis | 7 | ^8-14^ |
| Network meta-analysis | 3 | ^15-17^ |
| Not randomized controlled trial | 5 | ^18-22^ |
| Provide data after cross-over only and not apply sufficient wash-out period | 1 | ^23^ |
| Quasi randomized controlled trial but not a true randomized controlled trial | 2 | ^24,25^ |
| Review article | 5 | ^26-30^ |
| Study protocol but not result of a randomized controlled trial | 1 | ^31^ |
| Transient treatment but not a whole course treatment | 4 | ^32-35^ |

**eTable 4: Characteristics of the included studies**

| Study name | Diagnosis of OCD | Comparison | Numbers | Mean age | Female (%) | Treatment duration | Study duration^$^ | Country |
| --- | --- | --- | --- | --- | --- | --- | --- | --- |
| Guo, Q. (2022)^36^ | DSM-5 | cTBS over bilateral SMA Sham control | 26 24 | 35.0±9.5 30.3±7.8 | 30.8 37.5 | 4 weeks | 4 weeks + 4 weeks | China |
| Jahanbakhsh, G. (2022)^37^ | DSM-5 | 1 Hz rTMS over left DLPFC Sham control | 15 15 | 34.1±8.3 34.5±9.8 | 60.0 73.3 | 5 weeks | 5 weeks + 12 weeks | Iran |
| Joshi, M. (2022)^38^ | ICD-10 | 1 Hz rTMS over SMA (15% of the distance between nasion and inion anterior to the vertex) Sham control | 13 11 | 31.9±7.6 25.4±5.1 | 53.8 27.3 | 3 weeks | 3 weeks + 0 week | India |
| Khedr, E.M. (2022)^39^ | DSM-5 | 1 Hz rTMS over right DLPFC 1 Hz rTMS over right OFC Sham control | 20 20 20 | 36.9±11.5 34.1±10.2 35.4±9.4 | 55.0 55.0 50.0 | 2 weeks | 2 weeks + 12 weeks | Egypt |
| Vidya, K.L. (2022)^40^ | ICD-10 | 6 Hz rTMS over left SMA for priming + 1 Hz rTMS over left SMA Sham control for priming + 1 Hz rTMS over left SMA | 15 15 | 30.9±12.9 36.3±13.1 | 60.0 60.0 | 2 weeks | 2 weeks + 2 weeks | India |
| Dutta, P. (2021)^41^ | ICD-10 | cTBS over left OFC Sham control | 18 15 | 30.5±12.4 28.3±7.4 | 72.2 26.7 | 1 week | 1 week + 2 weeks | India |
| Ji, G.J. (2021)^42^ | DSM-5 | neuronavigated 1 Hz rTMS over right pre-SMA Sham control | 20 17 | 27.8±1.6 27.7±1.7 | 25.0 29.4 | 2 weeks | 2 weeks + 0 week | China |
| Liu, W. (2021)^43^ | DSM-5 | cTBS over right OFC Sham control | 12 11 | 28.2±9.8 31.0±7.5 | 58.3 54.5 | 2 weeks | 2 weeks + 4 weeks | China |
| Meek, B.P. (2021)^44^ | DSM-5 | 1 Hz rTMS over dorsal anterior cingulate cortex (dACC) Sham control | 10 10 | 45.0±16.7 38.3±11.5 | 60.0 40.0 | 2 weeks | 2 weeks + 12 weeks | Canada |
| Silva, R.M.F.D. (2021)^45^ | DSM-IV | 2 mA cathode tDCS over SMA (1.5 cm anteriorly to the measured location of Cz) + anode over left deltoid muscle Sham control | 22 21 | 38.4±11.0 36.9±12.2 | 59.1 61.9 | 4 weeks | 4 weeks + 8 weeks | Brazil |
| Yoosefee, S. (2020)^46^ | DSM-5 | 2 mA anode tDCS over left DLPFC + cathode over right lateral aspect of orbit Sham control | 30 30 | 38.6±12.6 35.9±11.5 | 86.7 76.7 | 8 weeks | 8 weeks + 4 weeks | Iran |
| Bation, R. (2019)^47^ | DSM-IV | 2 mA cathode tDCS over left OFC + anode over right cerebellum Sham control | 10 11 | 44.8±19.9 41.2±11.9 | 80.0 36.4 | 1 week | 1 week + 12 weeks | France |
| Carmi, L. (2019)^48^ | DSM-IV | 20 Hz dTMS over bilateral dorsal mPFC and ACC Sham control | 47 47 | 41.1±12.0 36.5±11.4 | 57.4 59.6 | 6 weeks | 6 weeks + 4 weeks | Multiple countries |
| Gowda, S.M. (2019)^49^ | DSM-IV | 2 mA anode tDCS over left pre-SMA + cathode over right supra-orbital area Sham control | 12 13 | 30.8±5.9 25.9±5.2 | 33.3 0.0 | 1 week | 1 week + 1 week | India |
| Zhang, K. (2019)^50^ | DSM-IV | 1 Hz rTMS over pre-SMA (15% of the distance between nasion and inion anterior to the vertex) Sham control | 25 24 | 32.2±13.3 39.4±17.0 | 40.0 41.7 | 4 weeks | 4 weeks + 0 week | China |
| Arumugham, S.S. (2018)^51^ | MINI (DSM/ICD) | 1 Hz rTMS over bilateral pre-SMA Sham control | 19 17 | 27.7±7.9 30.7±10.4 | 15.8 29.4 | 3 weeks | 3 weeks + 9 weeks | India |
| Carmi, L. (2018)^52^ | DSM-IV | 1 Hz dTMS over bilateral dorsal mPFC and ACC 20 Hz dTMS over bilateral dorsal mPFC and ACC Sham control | 8 16 14 | 28.0±3.1 36.0±2.1 35.0±3.5 | 50.0 43.8 50.0 | 5 weeks | 5 weeks + 4 weeks | Israel |
| D'Urso, G. (2016)^53^ | DSM-IV-TR | 2 mA cathode tDCS over pre-SMA (1.5 cm anteriorly to the measured location of Cz) + anode over right deltoid muscle 2 mA anode tDCS over pre-SMA (1.5 cm anteriorly to the measured location of Cz) + cathode over right deltoid muscle | 6 6 | 39.0±13.1 | 58.3 | 2 weeks | 2 weeks + 0 week | Italy |
| Elbeh, K.A.M. (2016)^54^ | DSM-IV-TR | 1 Hz rTMS over right DLPFC 10 Hz rTMS over right DLPFC Sham control | 15 15 15 | 26.8±5.2 28.9±3.9 25.5±4.0 | 26.7 40.0 33.3 | 2 weeks | 2 weeks + 12 weeks | Egypt |
| Hawken, E.R. (2016)^55^ | MINI (DSM/ICD) | 1 Hz rTMS over bilateral SMA (15% of the distance between inion and nasion, anterior to vertex (Cz)) Sham control | 10 12 | 33.0±10.0 34.0±14.0 | 50.0 | 6 weeks | 6 weeks + 0 week | Multiple countries |
| Pelissolo, A. (2016)^56^ | DSM-IV-TR | neuronavigated 1 Hz rTMS over bilateral pre-SMA Sham control | 20 16 | 39.1±10.4 42.3±10.6 | 65.0 56.0 | 4 weeks | 4 weeks + 8 weeks | France |
| Seo, H.J. (2016)^57^ | DSM-IV-TR | 1 Hz rTMS over right DLPFC Sham control | 14 13 | 34.6±9.8 36.3±12.5 | 42.9 53.8 | 3 weeks | 3 weeks + 0 week | Korea |
| Haghighi, M. (2015)^58,#^ | DSM-IV | 20 Hz rTMS over left DLPFC + right DLPFC Sham control | 10 11 | 34.9±5.9 36.6±4.0 | 30.0 54.5 | 2 weeks | 2 weeks + 0 week | Iran |
| Ma, X. (2014)^59^ | DSM-IV | alpha EEG guided-TMS over bilateral DLPFC Sham control | 25 21 | 27.1±9.0 29.9±9.4 | 32.0 38.1 | 2 weeks | 2 weeks + 1 week | China |
| Nauczyciel, C. (2014)^60,@^ | MINI (DSM/ICD) | 1 Hz rTMS over right OFC Sham control | 10 9 | 40.0 39.0 | 80.0 77.8 | 1 week | 1 week + 4 weeks | France |
| Gomes, P.V. (2012)^61^ | DSM-IV-TR | 1 Hz rTMS over bilateral pre-SMA Sham control | 12 10 | 35.5±7.5 37.5±16.0 | 66.7 50.0 | 2 weeks | 2 weeks + 12 weeks | Brazil |
| Mansur, C.G. (2011)^62^ | DSM-IV | 10 Hz rTMS over right DLPFC Sham control | 13 14 | 42.1±11.9 39.3±13.9 | 46.2 57.1 | 6 weeks | 6 weeks + 6 weeks | Brazil |
| Mantovani, A. (2010)^63^ | DSM-IV | 1 Hz rTMS over bilateral pre-SMA Sham control | 9 9 | 39.7±8.6 39.4±10.2 | 44.4 33.3 | 4 weeks | 4 weeks + 0 week | USA |
| Kang, J.I. (2009)^64^ | DSM-IV | 1 Hz rTMS over right DLPFC Sham control | 10 10 | 28.6±12.7 26.2±10.5 | 20.0 10.0 | 2 weeks | 2 weeks + 2 weeks | Korea |
| Ruffini, C. (2009)^65^ | DSM-IV-TR | 1 Hz rTMS over left OFC Sham control | 16 7 | NA | NA | 3 weeks | 3 weeks + 12 weeks | Italy |
| Sachdev, P.S. (2007)^66^ | DSM-IV | 10 Hz rTMS over left DLPFC Sham control | 10 8 | 29.5±9.9 35.8±8.2 | 70.0 37.5 | 2 weeks | 2 weeks + 24 weeks | Australia |
| Prasko, J. (2006)^67^ | DSM-IV | 1 Hz rTMS over left DLPFC Sham control | 18 12 | 28.9±7.7 33.4±8.7 | 27.8 58.3 | 2 weeks | 2 weeks + 2 weeks | Czech |
| Alonso, P. (2001)^68^ | DSM-IV | 1 Hz rTMS over right DLPFC Sham control | 10 8 | 39.2±13.0 30.3±9.5 | 80.0 50.0 | 6 weeks | 6 weeks + 4 weeks | Spain |
| Sachdev, P.S. (2001)^69^ | DSM-IV | 10 Hz rTMS over right DLPFC 10 Hz rTMS over left DLPFC | 6 6 | 40.5±13.4 | 25.0 | 2 weeks | 2 weeks + 4 weeks | Australia |

^$^: study duration = treatment duration + post-treatment follow-up duration

^#^: extract data before cross-over

^@^: cross-over study. It has 1 month wash-out period, which is sufficient to avoid carry-over effect

Abbreviation: cTBS: continuous TBS; ACC: anterior cingulate cortex; DLPFC: dorsolateral prefrontal cortex; DSM: diagnostic and statistical manual of mental disorders; dTMS: deep TMS; EEG: electroencephalography; ICD: international classification of diseases; mPFC: medial prefrontal cortex; NA: not available; OCD: obsessive-compulsive disorder; OFC: orbitofrontal cortex; rTMS: repetitive TMS; SMA: supplementary motor are; TBS: theta burst stimulation; tDCS: transcranial direct current stimulation; TMS: transcranial magnetic stimulation

**eTable 5A: SUCRA of the overall OCD severity (Y-BOCS)**

| Treatment | SUCRA |
| --- | --- |
| Hf-rTMS-F3+F4 | 10.3 |
| Hf-dTMS-Fp1Fp2 | 11.0 |
| c-tDCS-Fp2 + a-tDCS-F5 | 19.1 |
| Lf-rTMS-F4 | 30.8 |
| Lf-dTMS-Fp1Fp2 | 34.0 |
| Lf-rTMS-F5+F6 | 34.1 |
| Lf-rTMS-Fz | 42.0 |
| Lf-rTMS-Fp1 | 44.3 |
| neuronavigated Lf-rTMS-F6 | 46.2 |
| c-tDCS-Fp1 + a-tDCS-O2 | 48.2 |
| c-tDCS-Fz + a-tDCS-extracephalic | 49.4 |
| Lf-rTMS-Fp2 | 49.7 |
| alpha-TMS-F3+F4 | 52.1 |
| cTBS-F3+F4 | 53.9 |
| cTBS-Fp2 | 55.4 |
| cTBS-Fp1 | 60.1 |
| prLf-rTMS-F3 | 61.6 |
| Lf-rTMS-F3 | 61.7 |
| Hf-rTMS-F4 | 64.9 |
| Sham | 67.2 |
| a-tDCS-Fp3 + c-tDCS-F8 | 67.4 |
| neuronavigated Lf-rTMS-F5+F6 | 69.3 |
| Hf-rTMS-F3 | 82.0 |
| a-tDCS-Fz + c-tDCS-extracephalic | 85.3 |

Sorted by order of mean rank of improvement of overall OCD severity (the former, the better improvement of severity)

**eTable 5B: SUCRA of the overall OCD severity (Y-BOCS): subgroup of sham control**

| Treatment | SUCRA |
| --- | --- |
| Hf-rTMS-F3+F4 | 11.6 |
| Hf-dTMS-Fp1Fp2 | 12.3 |
| c-tDCS-Fp2 + a-tDCS-F5 | 21.6 |
| Lf-rTMS-F4 | 33.8 |
| Lf-dTMS-Fp1Fp2 | 35.6 |
| Lf-rTMS-F5+F6 | 37.2 |
| Lf-rTMS-Fz | 46.4 |
| Lf-rTMS-Fp1 | 47.7 |
| neuronavigated Lf-rTMS-F6 | 48.9 |
| c-tDCS-Fp1 + a-tDCS-O2 | 52.1 |
| c-tDCS-Fz + a-tDCS-extracephalic | 52.2 |
| Lf-rTMS-Fp2 | 54.3 |
| alpha-TMS-F3+F4 | 55.4 |
| cTBS-F3+F4 | 58.0 |
| cTBS-Fp2 | 60.3 |
| cTBS-Fp1 | 64.3 |
| Lf-rTMS-F3 | 68.1 |
| Hf-rTMS-F4 | 69.8 |
| Sham | 72.6 |
| a-tDCS-Fp3 + c-tDCS-F8 | 73.0 |
| neuronavigated Lf-rTMS-F5+F6 | 74.7 |

Sorted by order of mean rank of improvement of overall OCD severity (the former, the better improvement of severity)

**eTable 5C: SUCRA of the acceptability in aspect of drop-out rate**

| Treatment | SUCRA |
| --- | --- |
| c-tDCS-Fz + a-tDCS-extracephalic | 83.9 |
| cTBS-Fp2 | 68.2 |
| Lf-rTMS-F3 | 64.0 |
| Lf-rTMS-F5+F6 | 63.5 |
| a-tDCS-Fz + c-tDCS-extracephalic | 56.7 |
| neuronavigated Lf-rTMS-F5+F6 | 56.2 |
| Sham | 55.4 |
| Lf-rTMS-Fz | 54.1 |
| Hf-dTMS-Fp1Fp2 | 46.6 |
| Lf-dTMS-Fp1Fp2 | 45.7 |
| Hf-rTMS-F4 | 35.5 |
| cTBS-F3+F4 | 29.1 |
| a-tDCS-Fp3 + c-tDCS-F8 | 21.1 |
| Lf-rTMS-Fp2 | 19.8 |

Sorted by order of mean rank of drop-out rate (the former, the less drop-out rate)

**eTable 5D: SUCRA of the clinical impression (CGI-S)**

| Treatment | SUCRA |
| --- | --- |
| Hf-rTMS-F3+F4 | 0.7 |
| Lf-rTMS-F4 | 21.3 |
| alpha-TMS-F3+F4 | 25.6 |
| Lf-rTMS-Fz | 33.1 |
| Lf-rTMS-Fp2 | 40.7 |
| c-tDCS-Fp1 + a-tDCS-O2 | 42.9 |
| cTBS-Fp1 | 47.5 |
| Lf-rTMS-F5+F6 | 47.9 |
| prLf-rTMS-F3 | 65.0 |
| Hf-rTMS-F4 | 73.4 |
| neuronavigated Lf-rTMS-F5+F6 | 77.4 |
| Sham | 78.0 |
| Lf-rTMS-F3 | 96.6 |

Sorted by order of mean rank of improvement of clinical impression (the former, the better improvement of clinical impression)

**eTable 5E: SUCRA of the changes of depression**

| Treatment | SUCRA |
| --- | --- |
| Lf-rTMS-F4 | 24.3 |
| cTBS-Fp2 | 28.0 |
| alpha-TMS-F3+F4 | 31.8 |
| cTBS-F3+F4 | 34.3 |
| Lf-rTMS-Fz | 35.2 |
| Hf-rTMS-F3 | 41.2 |
| Hf-rTMS-F4 | 41.5 |
| c-tDCS-Fp1 + a-tDCS-O2 | 45.0 |
| Lf-rTMS-Fp2 | 52.6 |
| c-tDCS-Fz + a-tDCS-extracephalic | 55.4 |
| Lf-rTMS-F5+F6 | 66.7 |
| Sham | 74.4 |
| neuronavigated Lf-rTMS-F5+F6 | 76.5 |
| cTBS-Fp1 | 93.0 |

Sorted by order of mean rank of improvement of depression (the former, the better improvement of depression)

**eTable 5F: SUCRA of the changes of anxiety symptoms**

| Treatment | SUCRA |
| --- | --- |
| Lf-rTMS-F4 | 16.2 |
| Lf-rTMS-Fp2 | 19.8 |
| prLf-rTMS-F3 | 28.0 |
| cTBS-Fp2 | 34.0 |
| Lf-rTMS-F5+F6 | 35.6 |
| cTBS-F3+F4 | 37.2 |
| c-tDCS-Fp1 + a-tDCS-O2 | 42.8 |
| c-tDCS-Fz + a-tDCS-extracephalic | 47.1 |
| neuronavigated Lf-rTMS-F5+F6 | 56.7 |
| Lf-rTMS-F3 | 58.6 |
| Lf-rTMS-Fz | 61.0 |
| alpha-TMS-F3+F4 | 63.6 |
| Sham | 64.8 |
| Hf-rTMS-F3 | 72.0 |
| Hf-rTMS-F4 | 72.8 |
| cTBS-Fp1 | 89.7 |

Sorted by order of mean rank of improvement of anxiety symptoms (the former, the better improvement of anxiety symptoms)

**eTable 5G: SUCRA of the response rate**

| Treatment | SUCRA |
| --- | --- |
| Lf-rTMS-Fz | 10.0 |
| Hf-rTMS-F3+F4 | 15.6 |
| alpha-TMS-F3+F4 | 17.6 |
| c-tDCS-Fp2 + a-tDCS-F5 | 26.0 |
| Lf-rTMS-Fp2 | 34.0 |
| neuronavigated Lf-rTMS-F6 | 35.7 |
| Lf-rTMS-Fp1 | 40.8 |
| Hf-dTMS-Fp1Fp2 | 44.5 |
| c-tDCS-Fz + a-tDCS-extracephalic | 46.6 |
| Lf-rTMS-F4 | 48.5 |
| Lf-rTMS-F5+F6 | 52.5 |
| cTBS-Fp1 | 57.3 |
| c-tDCS-Fp1 + a-tDCS-O2 | 57.7 |
| cTBS-F3+F4 | 67.1 |
| cTBS-Fp2 | 71.1 |
| Hf-rTMS-F3 | 74.8 |
| Hf-rTMS-F4 | 76.5 |
| Sham | 83.4 |
| neuronavigated Lf-rTMS-F5+F6 | 90.4 |

Sorted by order of mean rank of response rate (the former, the better response rate)

Abbreviation: 95%CIs: 95% confidence intervals; ACC: anterior cingulate cortex; alpha-TMS-F3+F4: alpha EEG guided-TMS over F3+F4; a-tDCS-Fp3 + c-tDCS-F8: anode tDCS over Fp3 plus cathode tDCS over F8; a-tDCS-Fz + c-tDCS-extracephalic: anode tDCS over Fz plus cathode tDCS over extracephalic region; CBT: cognitive behavioral therapy; CGI-S: clinical global impression scale-severity; cTBS: continuous theta burst stimulation; cTBS-F3+F4: cTBS over F3+F4; cTBS-Fp1: cTBS over Fp1; cTBS-Fp2: cTBS over Fp2; c-tDCS-Fp1 + a-tDCS-O2: cathode tDCS over Fp1 plus anode tDCS over O2; c-tDCS-Fp2 + a-tDCS-F5: cathode tDCS over Fp2 plus anode tDCS over F5; c-tDCS-Fz + a-tDCS-extracephalic: cathode tDCS over Fz plus anode tDCS over extracephalic region; DLPFC: dorsolateral prefrontal cortex; dmPFC: dorsal medial prefrontal cortex; DSM: diagnostic and statistical manual of mental disorders; dTMS: deep TMS; EEG: electroencephalography; Hf-dTMS-Fp1Fp2: high frequency dTMS over Fp1Fp2; Hf-rTMS-F3: high frequency rTMS over F3; Hf-rTMS-F3+F4: high frequency rTMS over F3+F4; Hf-rTMS-F4: high frequency rTMS over F4; ICD: international classification of diseases; Lf-dTMS-Fp1Fp2: low frequency dTMS over Fp1Fp2; Lf-rTMS-F3: low frequency rTMS over F3; Lf-rTMS-F4: low frequency rTMS over F4; Lf-rTMS-F5+F6: low frequency rTMS over F5+F6; Lf-rTMS-Fp1: low frequency rTMS over Fp1; Lf-rTMS-Fp2: low frequency rTMS over Fp2; Lf-rTMS-Fz: low frequency rTMS over Fz; MD: mean difference; neuronavigated Lf-rTMS-F5+F6: neuronavigated low frequency rTMS over F5+F6; neuronavigated Lf-rTMS-F6: neuronavigated low frequency rTMS over F6; NIBS: noninvasive brain stimulation; NMA: network meta-analysis; OCD: obsessive-compulsive disorder; OFC: orbitofrontal cortex; OR: odds ratio; prLf-rTMS-F3: priming and low frequency rTMS over F3; RCT: randomized controlled trial; rTMS: repetitive TMS; Sham: sham control; SMD: standardized mean difference; SUCRA: surface under the cumulative ranking curve; tDCS: transcranial direct current stimulation; TMS: transcranial magnetic stimulation; YBOCS: Yale-Brown obsessive compulsive scale

**eTable 6A: League table of the overall OCD severity (Y-BOCS): subgroup of sham control**

| Hf-rTMS-F3+F4 |  |  |  |  |  |  |  |  |  |  |  |  |  |  |  |  |  | ***-10.81 (-17.46,-4.16)** |  |  |
| --- | --- | --- | --- | --- | --- | --- | --- | --- | --- | --- | --- | --- | --- | --- | --- | --- | --- | --- | --- | --- |
| -1.07 (-13.09,10.95) | Hf-dTMS-Fp1Fp2 |  |  | ***-11.78 (-20.60,-3.00)** |  |  |  |  |  |  |  |  |  |  |  |  |  | -11.96 (-31.26,7.35) |  |  |
| -0.79 (-19.67,18.09) | 0.28 (-17.08,17.64) | c-tDCS-Fp2 + a-tDCS-F5 |  |  |  |  |  |  |  |  |  |  |  |  |  |  |  | -10.02 (-24.20,4.16) |  |  |
| -6.11 (-16.92,4.71) | -5.04 (-12.91,2.84) | -5.32 (-21.86,11.23) | Lf-rTMS-F4 |  |  |  |  |  |  |  | -1.70 (-7.51,4.11) |  |  |  |  |  | ***-7.10 (-11.86,-2.34)** | ***-4.77 (-7.52,-2.03)** |  |  |
| -5.46 (-19.61,8.68) | -4.40 (-14.96,6.17) | -4.67 (-23.57,14.22) | 0.64 (-10.19,11.47) | Lf-dTMS-Fp1Fp2 |  |  |  |  |  |  |  |  |  |  |  |  |  | ***-10.33 (-17.80,-2.86)** |  |  |
| -6.44 (-17.70,4.81) | -5.37 (-13.85,3.10) | -5.65 (-22.49,11.19) | -0.34 (-6.96,6.29) | -0.98 (-12.25,10.29) | Lf-rTMS-F5+F6 |  |  |  |  |  |  |  |  |  |  |  |  | ***-4.78 (-7.35,-2.21)** |  |  |
| -7.65 (-18.49,3.20) | -6.58 (-14.39,1.24) | -6.86 (-23.42,9.71) | -1.54 (-7.45,4.37) | -2.18 (-13.04,8.68) | -1.21 (-7.88,5.47) | Lf-rTMS-Fz |  |  |  |  |  |  |  |  |  |  |  | ***-2.81 (-3.91,-1.71)** |  |  |
| -7.73 (-20.63,5.17) | -6.66 (-17.20,3.88) | -6.94 (-24.92,11.04) | -1.62 (-10.76,7.52) | -2.27 (-15.17,10.64) | -1.29 (-10.94,8.37) | -0.08 (-9.26,9.09) | Lf-rTMS-Fp1 |  |  |  |  |  |  |  |  |  |  | -3.08 (-6.37,0.21) |  |  |
| -8.03 (-21.01,4.95) | -6.96 (-17.60,3.68) | -7.24 (-25.28,10.80) | -1.92 (-11.18,7.33) | -2.57 (-15.56,10.43) | -1.59 (-11.35,8.18) | -0.38 (-9.67,8.91) | -0.30 (-11.92,11.32) | neuronavigated Lf-rTMS-F6 |  |  |  |  |  |  |  |  |  | -2.78 (-6.38,0.82) |  |  |
| -8.44 (-21.87,4.99) | -7.37 (-18.56,3.82) | -7.65 (-26.01,10.71) | -2.33 (-12.22,7.55) | -2.98 (-16.42,10.47) | -2.00 (-12.36,8.36) | -0.79 (-10.71,9.12) | -0.71 (-12.83,11.41) | -0.41 (-12.62,11.80) | c-tDCS-Fp1 + a-tDCS-O2 |  |  |  |  |  |  |  |  | -2.37 (-7.36,2.62) |  |  |
| -8.50 (-21.54,4.54) | -7.43 (-18.14,3.28) | -7.71 (-25.79,10.37) | -2.39 (-11.73,6.95) | -3.04 (-16.09,10.01) | -2.06 (-11.90,7.79) | -0.85 (-10.23,8.52) | -0.77 (-12.46,10.92) | -0.47 (-12.25,11.31) | -0.06 (-12.34,12.22) | c-tDCS-Fz + a-tDCS-extracephalic |  |  |  |  |  |  |  | -2.31 (-6.12,1.50) |  |  |
| -8.71 (-20.60,3.18) | -7.64 (-16.93,1.66) | -7.92 (-25.19,9.35) | -2.60 (-9.60,4.40) | -3.24 (-15.15,8.66) | -2.27 (-10.53,6.00) | -1.06 (-8.76,6.64) | -0.98 (-11.37,9.41) | -0.68 (-11.17,9.82) | -0.27 (-11.32,10.78) | -0.21 (-10.77,10.36) | Lf-rTMS-Fp2 |  |  |  |  |  |  | -2.13 (-6.32,2.06) |  |  |
| -8.91 (-21.96,4.14) | -7.84 (-18.57,2.89) | -8.12 (-26.21,9.97) | -2.80 (-12.16,6.55) | -3.45 (-16.51,9.62) | -2.47 (-12.33,7.39) | -1.26 (-10.65,8.13) | -1.18 (-12.88,10.52) | -0.88 (-12.67,10.91) | -0.47 (-12.76,11.82) | -0.41 (-12.27,11.45) | -0.20 (-10.78,10.38) | alpha-TMS-F3+F4 |  |  |  |  |  | -1.90 (-5.75,1.95) |  |  |
| -9.34 (-22.32,3.64) | -8.27 (-18.92,2.37) | -8.55 (-26.59,9.49) | -3.24 (-12.50,6.02) | -3.88 (-16.87,9.12) | -2.90 (-12.67,6.87) | -1.69 (-10.99,7.60) | -1.61 (-13.24,10.01) | -1.31 (-13.03,10.40) | -0.90 (-13.12,11.31) | -0.84 (-12.62,10.94) | -0.63 (-11.13,9.86) | -0.43 (-12.23,11.36) | cTBS-F3+F4 |  |  |  |  | -1.47 (-5.08,2.14) |  |  |
| -9.49 (-22.36,3.38) | -8.42 (-18.93,2.08) | -8.70 (-26.66,9.26) | -3.38 (-12.49,5.72) | -4.03 (-16.91,8.86) | -3.05 (-12.67,6.57) | -1.84 (-10.98,7.30) | -1.76 (-13.26,9.74) | -1.46 (-13.05,10.13) | -1.05 (-13.15,11.05) | -0.99 (-12.65,10.67) | -0.78 (-11.14,9.58) | -0.58 (-12.25,11.09) | -0.15 (-11.74,11.45) | cTBS-Fp2 |  |  |  | -1.32 (-4.51,1.87) |  |  |
| -10.30 (-22.87,2.26) | -9.24 (-19.36,0.89) | -9.51 (-27.25,8.22) | -4.20 (-12.86,4.46) | -4.84 (-17.42,7.73) | -3.86 (-13.07,5.34) | -2.66 (-11.36,6.04) | -2.58 (-13.73,8.58) | -2.28 (-13.52,8.97) | -1.87 (-13.63,9.90) | -1.80 (-13.12,9.51) | -1.60 (-11.57,8.38) | -1.39 (-12.73,9.94) | -0.96 (-12.21,10.29) | -0.81 (-11.94,10.31) | cTBS-Fp1 |  |  | -0.51 (-2.03,1.02) |  |  |
| -10.39 (-21.98,1.21) | ***-9.32 (-18.37,-0.26)** | -9.60 (-26.66,7.47) | -4.28 (-11.45,2.89) | -4.92 (-16.53,6.69) | -3.94 (-11.76,3.87) | -2.74 (-10.00,4.52) | -2.66 (-12.70,7.39) | -2.36 (-12.51,7.80) | -1.95 (-12.67,8.78) | -1.89 (-12.11,8.34) | -1.68 (-10.39,7.03) | -1.48 (-11.72,8.77) | -1.04 (-11.20,9.11) | -0.90 (-10.91,9.12) | -0.08 (-9.69,9.53) | Lf-rTMS-F3 |  | -0.05 (-8.03,7.92) |  |  |
| -10.97 (-22.67,0.74) | ***-9.90 (-18.92,-0.88)** | -10.18 (-27.32,6.96) | -4.86 (-11.43,1.71) | -5.50 (-17.22,6.21) | -4.52 (-12.52,3.47) | -3.32 (-10.72,4.08) | -3.24 (-13.41,6.94) | -2.94 (-13.22,7.34) | -2.53 (-13.37,8.32) | -2.47 (-12.82,7.89) | -2.26 (-10.95,6.43) | -2.06 (-12.43,8.32) | -1.62 (-11.91,8.66) | -1.48 (-11.62,8.67) | -0.66 (-10.41,9.09) | -0.58 (-9.05,7.89) | Hf-rTMS-F4 | -0.68 (-4.61,3.25) |  |  |
| ***-10.81 (-20.80,-0.82)** | ***-9.74 (-16.42,-3.06)** | -10.02 (-26.04,6.00) | ***-4.70 (-8.84,-0.57)** | -5.35 (-15.36,4.66) | -4.37 (-9.54,0.81) | -3.16 (-7.37,1.05) | -3.08 (-11.23,5.07) | -2.78 (-11.06,5.50) | -2.37 (-11.35,6.61) | -2.31 (-10.68,6.06) | -2.10 (-8.55,4.34) | -1.90 (-10.29,6.49) | -1.47 (-9.75,6.82) | -1.32 (-9.43,6.79) | -0.51 (-8.12,7.11) | -0.42 (-6.30,5.45) | 0.16 (-5.94,6.25) | Sham | -1.00 (-6.23,4.23) | -1.20 (-4.45,2.05) |
| -11.81 (-25.33,1.71) | -10.74 (-22.03,0.55) | -11.02 (-29.45,7.41) | -5.70 (-15.71,4.30) | -6.35 (-19.88,7.19) | -5.37 (-15.84,5.11) | -4.16 (-14.20,5.87) | -4.08 (-16.30,8.14) | -3.78 (-16.09,8.53) | -3.37 (-16.16,9.42) | -3.31 (-15.68,9.06) | -3.10 (-14.26,8.05) | -2.90 (-15.29,9.49) | -2.47 (-14.78,9.84) | -2.32 (-14.51,9.87) | -1.51 (-13.37,10.36) | -1.42 (-12.26,9.41) | -0.84 (-11.80,10.11) | -1.00 (-10.11,8.11) | a-tDCS-Fp3 + c-tDCS-F8 |  |
| -12.01 (-24.90,0.88) | *-10.94 (-21.47,-0.41) | -11.22 (-29.19,6.75) | -5.90 (-15.03,3.22) | -6.55 (-19.44,6.35) | -5.57 (-15.21,4.07) | -4.36 (-13.52,4.80) | -4.28 (-15.80,7.24) | -3.98 (-15.59,7.63) | -3.57 (-15.68,8.54) | -3.51 (-15.19,8.17) | -3.30 (-13.68,7.08) | -3.10 (-14.79,8.59) | -2.67 (-14.28,8.94) | -2.52 (-14.01,8.97) | -1.71 (-12.85,9.44) | -1.62 (-11.66,8.41) | -1.04 (-11.21,9.12) | -1.20 (-9.34,6.94) | -0.20 (-12.41,12.01) | neuronavigated Lf-rTMS-F5+F6 |

Pairwise (upper-right portion) and network (lower-left portion) meta-analysis results are presented as estimate effect sizes for the outcome of improvement of overall OCD severity. Interventions are reported in order of mean ranking of severity improvement, and outcomes are expressed as mean difference (MD) (95% confidence intervals). For the pairwise meta-analyses, MD of less than 0 indicate that the treatment specified in the row got more improvement than that specified in the column. For the network meta-analysis (NMA), MD of less than 0 indicate that the treatment specified in the column got more improvement than that specified in the row. Bold results marked with * indicate statistical significance.

**eTable 6B: League table of the clinical impression (CGI-S)**

| Hf-rTMS-F3+F4 |  |  |  |  |  |  |  |  |  |  | ***-2.49 (-3.61,-1.37)** |  |
| --- | --- | --- | --- | --- | --- | --- | --- | --- | --- | --- | --- | --- |
| ***-1.48 (-2.68,-0.28)** | Lf-rTMS-F4 |  |  | -0.20 (-1.16,0.76) |  |  |  |  | -0.90 (-1.84,0.04) |  | ***-1.02 (-1.46,-0.58)** |  |
| ***-1.58 (-2.75,-0.41)** | -0.10 (-0.66,0.46) | alpha-TMS-F3+F4 |  |  |  |  |  |  |  |  | ***-0.91 (-1.27,-0.55)** |  |
| -1.49 (-3.51,0.53) | -0.01 (-1.74,1.73) | 0.09 (-1.63,1.81) | Lf-rTMS-Fz |  |  |  |  |  |  |  | -1.00 (-2.68,0.68) |  |
| ***-1.82 (-3.18,-0.46)** | -0.34 (-1.15,0.47) | -0.24 (-1.10,0.61) | -0.33 (-2.18,1.52) | Lf-rTMS-Fp2 |  |  |  |  |  |  | -0.62 (-1.44,0.21) |  |
| ***-1.87 (-3.38,-0.36)** | -0.39 (-1.48,0.71) | -0.29 (-1.36,0.78) | -0.38 (-2.34,1.58) | -0.05 (-1.32,1.23) | c-tDCS-Fp1 + a-tDCS-O2 |  |  |  |  |  | -0.62 (-1.63,0.39) |  |
| ***-1.96 (-3.21,-0.71)** | -0.48 (-1.18,0.23) | -0.38 (-1.04,0.28) | -0.47 (-2.24,1.30) | -0.14 (-1.09,0.82) | -0.09 (-1.24,1.06) | cTBS-Fp1 |  |  |  |  | -0.53 (-1.08,0.02) |  |
| ***-1.96 (-3.20,-0.73)** | -0.48 (-1.17,0.21) | -0.38 (-1.02,0.26) | -0.47 (-2.24,1.29) | -0.14 (-1.08,0.80) | -0.09 (-1.24,1.05) | -0.00 (-0.77,0.76) | Lf-rTMS-F5+F6 |  |  |  | -0.53 (-1.06,0.01) |  |
| ***-2.36 (-3.97,-0.76)** | -0.88 (-2.11,0.35) | -0.78 (-1.99,0.42) | -0.87 (-2.91,1.16) | -0.54 (-1.93,0.85) | -0.49 (-2.03,1.04) | -0.40 (-1.68,0.87) | -0.40 (-1.67,0.87) | prLf-rTMS-F3 |  |  |  | ***-0.88 (-1.38,-0.38)** |
| ***-2.45 (-3.74,-1.16)** | ***-0.97 (-1.65,-0.28)** | ***-0.87 (-1.61,-0.14)** | -0.96 (-2.76,0.84) | -0.63 (-1.61,0.35) | -0.58 (-1.78,0.61) | -0.49 (-1.34,0.36) | -0.49 (-1.32,0.35) | -0.09 (-1.41,1.23) | Hf-rTMS-F4 |  | 0.00 (-0.73,0.73) |  |
| ***-2.49 (-3.68,-1.30)** | ***-1.01 (-1.59,-0.42)** | ***-0.91 (-1.44,-0.38)** | -1.00 (-2.72,0.72) | -0.67 (-1.54,0.20) | -0.62 (-1.70,0.46) | -0.53 (-1.21,0.15) | -0.53 (-1.19,0.14) | -0.13 (-1.34,1.09) | -0.04 (-0.79,0.72) | neuronavigated Lf-rTMS-F5+F6 | 0.00 (-0.39,0.39) |  |
| ***-2.49 (-3.61,-1.37)** | ***-1.01 (-1.44,-0.57)** | ***-0.91 (-1.27,-0.55)** | -1.00 (-2.68,0.68) | -0.67 (-1.44,0.11) | -0.62 (-1.63,0.39) | -0.53 (-1.08,0.02) | -0.53 (-1.06,0.01) | -0.13 (-1.28,1.03) | -0.04 (-0.68,0.61) | 0.00 (-0.39,0.39) | Sham | -0.76 (-1.79,0.28) |
| ***-3.24 (-4.77,-1.72)** | ***-1.76 (-2.88,-0.64)** | ***-1.66 (-2.76,-0.57)** | -1.75 (-3.73,0.22) | ***-1.42 (-2.72,-0.13)** | -1.37 (-2.82,0.07) | ***-1.28 (-2.46,-0.11)** | ***-1.28 (-2.44,-0.12)** | ***-0.88 (-1.38,-0.38)** | -0.79 (-2.01,0.43) | -0.75 (-1.86,0.35) | -0.75 (-1.79,0.28) | Lf-rTMS-F3 |

Pairwise (upper-right portion) and network (lower-left portion) meta-analysis results are presented as estimate effect sizes for the outcome of improvement of clinical impression. Interventions are reported in order of mean ranking of clinical impression improvement, and outcomes are expressed as mean difference (MD) (95% confidence intervals). For the pairwise meta-analyses, MD of less than 0 indicate that the treatment specified in the row got more improvement than that specified in the column. For the network meta-analysis (NMA), MD of less than 0 indicate that the treatment specified in the column got more improvement than that specified in the row. Bold results marked with * indicate statistical significance.

**eTable 6C: League table of the changes of depression**

| Lf-rTMS-F4 |  |  |  |  |  |  |  | -0.46 (-1.08,0.17) |  |  | ***-0.53 (-1.05,-0.01)** |  |  |
| --- | --- | --- | --- | --- | --- | --- | --- | --- | --- | --- | --- | --- | --- |
| -0.01 (-0.95,0.93) | cTBS-Fp2 |  |  |  |  |  |  |  |  |  | -0.55 (-1.38,0.29) |  |  |
| -0.09 (-0.82,0.63) | -0.08 (-1.11,0.94) | alpha-TMS-F3+F4 |  |  |  |  |  |  |  |  | -0.46 (-1.05,0.12) |  |  |
| -0.13 (-0.83,0.58) | -0.11 (-1.12,0.89) | -0.03 (-0.84,0.78) | cTBS-F3+F4 |  |  |  |  |  |  |  | -0.43 (-1.00,0.13) |  |  |
| -0.15 (-0.74,0.45) | -0.14 (-1.07,0.80) | -0.05 (-0.77,0.67) | -0.02 (-0.72,0.68) | Lf-rTMS-Fz |  |  |  |  |  |  | -0.41 (-0.83,0.01) |  |  |
| -0.13 (-1.56,1.30) | -0.12 (-1.72,1.48) | -0.03 (-1.52,1.45) | -0.00 (-1.48,1.47) | 0.02 (-1.41,1.44) | Hf-rTMS-F3 | -0.06 (-1.19,1.07) |  |  |  |  |  |  |  |
| -0.19 (-1.06,0.68) | -0.18 (-1.31,0.95) | -0.09 (-1.06,0.87) | -0.06 (-1.01,0.88) | -0.04 (-0.91,0.83) | -0.06 (-1.19,1.07) | Hf-rTMS-F4 |  |  |  |  | -0.37 (-1.13,0.39) |  |  |
| -0.26 (-1.22,0.71) | -0.24 (-1.45,0.96) | -0.16 (-1.20,0.88) | -0.13 (-1.16,0.90) | -0.11 (-1.07,0.85) | -0.13 (-1.74,1.49) | -0.07 (-1.22,1.08) | c-tDCS-Fp1 + a-tDCS-O2 |  |  |  | -0.30 (-1.17,0.56) |  |  |
| -0.34 (-0.90,0.22) | -0.33 (-1.32,0.66) | -0.25 (-1.04,0.55) | -0.22 (-0.99,0.56) | -0.19 (-0.87,0.48) | -0.21 (-1.68,1.25) | -0.15 (-1.08,0.78) | -0.09 (-1.10,0.93) | Lf-rTMS-Fp2 |  |  | -0.30 (-0.87,0.26) |  |  |
| -0.37 (-1.11,0.36) | -0.36 (-1.39,0.67) | -0.28 (-1.12,0.56) | -0.25 (-1.07,0.57) | -0.23 (-0.96,0.51) | -0.24 (-1.73,1.25) | -0.18 (-1.15,0.79) | -0.12 (-1.17,0.93) | -0.03 (-0.83,0.77) | c-tDCS-Fz + a-tDCS-extracephalic |  | -0.19 (-0.79,0.41) |  |  |
| -0.50 (-1.12,0.12) | -0.49 (-1.44,0.46) | -0.41 (-1.15,0.33) | -0.38 (-1.10,0.34) | -0.36 (-0.97,0.26) | -0.37 (-1.81,1.06) | -0.32 (-1.20,0.57) | -0.25 (-1.22,0.72) | -0.16 (-0.86,0.53) | -0.13 (-0.88,0.62) | Lf-rTMS-F5+F6 | -0.06 (-0.51,0.40) |  |  |
| ***-0.56 (-0.99,-0.13)** | -0.55 (-1.38,0.29) | -0.46 (-1.05,0.12) | -0.43 (-1.00,0.13) | -0.41 (-0.83,0.01) | -0.43 (-1.80,0.93) | -0.37 (-1.13,0.39) | -0.30 (-1.17,0.56) | -0.22 (-0.75,0.31) | -0.19 (-0.79,0.41) | -0.06 (-0.51,0.40) | Sham | -0.10 (-0.75,0.56) | -0.49 (-1.18,0.21) |
| -0.66 (-1.44,0.13) | -0.64 (-1.71,0.42) | -0.56 (-1.44,0.32) | -0.53 (-1.40,0.33) | -0.51 (-1.29,0.27) | -0.53 (-2.04,0.99) | -0.47 (-1.47,0.54) | -0.40 (-1.49,0.68) | -0.32 (-1.16,0.53) | -0.28 (-1.17,0.61) | -0.15 (-0.95,0.65) | -0.10 (-0.75,0.56) | neuronavigated Lf-rTMS-F5+F6 |  |
| ***-1.05 (-1.86,-0.23)** | -1.03 (-2.12,0.05) | ***-0.95 (-1.86,-0.04)** | ***-0.92 (-1.81,-0.03)** | ***-0.90 (-1.71,-0.09)** | -0.92 (-2.45,0.62) | -0.86 (-1.89,0.18) | -0.79 (-1.90,0.32) | -0.70 (-1.58,0.17) | -0.67 (-1.59,0.25) | -0.54 (-1.37,0.29) | -0.49 (-1.18,0.21) | -0.39 (-1.35,0.57) | cTBS-Fp1 |

Pairwise (upper-right portion) and network (lower-left portion) meta-analysis results are presented as estimate effect sizes for the outcome of improvement of depression. Interventions are reported in order of mean ranking of depression improvement, and outcomes are expressed as standardized mean difference (SMD) (95% confidence intervals). For the pairwise meta-analyses, SMD of less than 0 indicate that the treatment specified in the row got more improvement than that specified in the column. For the network meta-analysis (NMA), SMD of less than 0 indicate that the treatment specified in the column got more improvement than that specified in the row. Bold results marked with * indicate statistical significance.

**eTable 6D: League table of the changes of anxiety symptoms**

| Lf-rTMS-F4 | -0.15 (-0.77,0.47) |  |  |  |  |  |  |  |  |  |  | ***-0.66 (-1.12,-0.21)** |  | ***-1.35 (-2.12,-0.58)** |  |
| --- | --- | --- | --- | --- | --- | --- | --- | --- | --- | --- | --- | --- | --- | --- | --- |
| -0.01 (-0.73,0.72) | Lf-rTMS-Fp2 |  |  |  |  |  |  |  |  |  |  | ***-0.86 (-1.50,-0.22)** |  |  |  |
| -0.07 (-1.40,1.26) | -0.06 (-1.51,1.39) | prLf-rTMS-F3 |  |  |  |  |  |  | -0.58 (-1.32,0.15) |  |  |  |  |  |  |
| -0.22 (-1.29,0.84) | -0.22 (-1.43,1.00) | -0.16 (-1.74,1.43) | cTBS-Fp2 |  |  |  |  |  |  |  |  | -0.49 (-1.32,0.34) |  |  |  |
| -0.31 (-1.03,0.40) | -0.31 (-1.22,0.61) | -0.25 (-1.61,1.12) | -0.09 (-1.21,1.02) | Lf-rTMS-F5+F6 |  |  |  |  |  |  |  | -0.41 (-1.08,0.26) |  |  |  |
| -0.33 (-1.20,0.54) | -0.32 (-1.37,0.72) | -0.26 (-1.72,1.20) | -0.11 (-1.33,1.12) | -0.02 (-0.95,0.91) | cTBS-F3+F4 |  |  |  |  |  |  | -0.38 (-0.94,0.18) |  |  |  |
| -0.39 (-1.48,0.70) | -0.38 (-1.61,0.85) | -0.32 (-1.92,1.28) | -0.16 (-1.55,1.23) | -0.07 (-1.21,1.07) | -0.06 (-1.30,1.19) | c-tDCS-Fp1 + a-tDCS-O2 |  |  |  |  |  | -0.33 (-1.19,0.54) |  |  |  |
| -0.48 (-1.38,0.42) | -0.47 (-1.54,0.60) | -0.41 (-1.88,1.06) | -0.25 (-1.50,0.99) | -0.16 (-1.12,0.79) | -0.15 (-1.23,0.93) | -0.09 (-1.35,1.17) | c-tDCS-Fz + a-tDCS-extracephalic |  |  |  |  | -0.24 (-0.84,0.37) |  |  |  |
| -0.67 (-1.60,0.27) | -0.66 (-1.76,0.44) | -0.60 (-2.09,0.90) | -0.44 (-1.71,0.83) | -0.35 (-1.34,0.64) | -0.34 (-1.45,0.78) | -0.28 (-1.57,1.01) | -0.19 (-1.32,0.94) | neuronavigated Lf-rTMS-F5+F6 |  |  |  | -0.05 (-0.70,0.61) |  |  |  |
| -0.65 (-1.64,0.34) | -0.64 (-1.79,0.50) | -0.58 (-1.47,0.30) | -0.43 (-1.74,0.88) | -0.34 (-1.38,0.70) | -0.32 (-1.48,0.84) | -0.26 (-1.60,1.07) | -0.17 (-1.35,1.00) | 0.01 (-1.19,1.22) | Lf-rTMS-F3 |  |  | -0.06 (-0.79,0.67) |  |  |  |
| -0.67 (-1.42,0.07) | -0.67 (-1.61,0.28) | -0.60 (-1.99,0.78) | -0.45 (-1.59,0.69) | -0.36 (-1.18,0.46) | -0.34 (-1.30,0.62) | -0.29 (-1.45,0.88) | -0.20 (-1.18,0.79) | -0.01 (-1.03,1.01) | -0.02 (-1.09,1.05) | Lf-rTMS-Fz |  | -0.05 (-0.52,0.43) |  |  |  |
| -0.73 (-1.61,0.16) | -0.72 (-1.77,0.34) | -0.66 (-2.12,0.81) | -0.50 (-1.73,0.73) | -0.41 (-1.35,0.53) | -0.39 (-1.46,0.67) | -0.34 (-1.59,0.92) | -0.25 (-1.34,0.84) | -0.06 (-1.18,1.06) | -0.07 (-1.24,1.09) | -0.05 (-1.02,0.92) | alpha-TMS-F3+F4 | 0.01 (-0.57,0.59) |  |  |  |
| ***-0.71 (-1.16,-0.27)** | -0.70 (-1.43,0.02) | -0.64 (-1.89,0.61) | -0.49 (-1.46,0.48) | -0.40 (-0.95,0.15) | -0.38 (-1.13,0.37) | -0.33 (-1.32,0.67) | -0.24 (-1.01,0.54) | -0.05 (-0.87,0.78) | -0.06 (-0.94,0.82) | -0.04 (-0.64,0.56) | 0.01 (-0.75,0.78) | Sham |  | -0.05 (-0.66,0.57) | -0.62 (-1.32,0.09) |
| -0.92 (-2.33,0.49) | -0.91 (-2.45,0.63) | -0.85 (-2.71,1.01) | -0.69 (-2.37,0.99) | -0.60 (-2.08,0.88) | -0.59 (-2.15,0.98) | -0.53 (-2.23,1.17) | -0.44 (-2.02,1.14) | -0.25 (-1.85,1.35) | -0.27 (-1.90,1.37) | -0.24 (-1.74,1.26) | -0.19 (-1.76,1.38) | -0.20 (-1.58,1.17) | Hf-rTMS-F3 | 0.05 (-1.09,1.18) |  |
| ***-0.87 (-1.55,-0.20)** | -0.86 (-1.78,0.05) | -0.80 (-2.19,0.59) | -0.65 (-1.79,0.49) | -0.56 (-1.37,0.26) | -0.54 (-1.50,0.42) | -0.48 (-1.65,0.68) | -0.39 (-1.38,0.59) | -0.20 (-1.22,0.81) | -0.22 (-1.29,0.85) | -0.20 (-1.05,0.65) | -0.14 (-1.12,0.83) | -0.16 (-0.76,0.44) | 0.05 (-1.19,1.28) | Hf-rTMS-F4 |  |
| ***-1.33 (-2.30,-0.36)** | ***-1.32 (-2.45,-0.19)** | -1.26 (-2.78,0.26) | -1.10 (-2.40,0.19) | -1.01 (-2.04,0.01) | -1.00 (-2.14,0.14) | -0.94 (-2.26,0.37) | -0.85 (-2.01,0.31) | -0.66 (-1.85,0.53) | -0.68 (-1.91,0.56) | -0.66 (-1.70,0.39) | -0.60 (-1.75,0.55) | -0.62 (-1.48,0.24) | -0.41 (-2.03,1.21) | -0.46 (-1.51,0.59) | cTBS-Fp1 |

Pairwise (upper-right portion) and network (lower-left portion) meta-analysis results are presented as estimate effect sizes for the outcome of improvement of anxiety symptoms. Interventions are reported in order of mean ranking of anxiety symptoms improvement, and outcomes are expressed as standardized mean difference (SMD) (95% confidence intervals). For the pairwise meta-analyses, SMD of less than 0 indicate that the treatment specified in the row got more improvement than that specified in the column. For the network meta-analysis (NMA), SMD of less than 0 indicate that the treatment specified in the column got more improvement than that specified in the row. Bold results marked with * indicate statistical significance.

**eTable 6E: League table of the response rate**

| Lf-rTMS-Fz |  |  |  |  |  |  |  |  |  |  |  |  |  |  |  |  | ***44.00 (3.38,573.41)** |  |
| --- | --- | --- | --- | --- | --- | --- | --- | --- | --- | --- | --- | --- | --- | --- | --- | --- | --- | --- |
| 1.32 (0.02,72.79) | Hf-rTMS-F3+F4 |  |  |  |  |  |  |  |  |  |  |  |  |  |  |  | ***33.22 (1.53,719.96)** |  |
| 1.78 (0.04,86.46) | 1.34 (0.02,92.95) | alpha-TMS-F3+F4 |  |  |  |  |  |  |  |  |  |  |  |  |  |  | ***24.76 (1.34,456.87)** |  |
| 3.08 (0.06,165.24) | 2.32 (0.03,176.22) | 1.73 (0.03,117.33) | c-tDCS-Fp2 + a-tDCS-F5 |  |  |  |  |  |  |  |  |  |  |  |  |  | 14.29 (0.68,300.37) |  |
| 6.36 (0.30,137.10) | 4.80 (0.14,160.15) | 3.58 (0.12,103.74) | 2.07 (0.06,67.07) | Lf-rTMS-Fp2 |  |  |  |  | 1.52 (0.43,5.41) |  |  |  |  |  |  |  | ***27.88 (1.48,526.12)** |  |
| 7.17 (0.33,157.49) | 5.41 (0.16,183.54) | 4.03 (0.14,118.98) | 2.33 (0.07,76.87) | 1.13 (0.10,12.50) | neuronavigated Lf-rTMS-F6 |  |  |  |  |  |  |  |  |  |  |  | ***6.14 (1.10,34.21)** |  |
| 7.33 (0.23,235.42) | 5.54 (0.12,262.96) | 4.13 (0.10,172.62) | 2.38 (0.05,110.40) | 1.15 (0.06,20.47) | 1.02 (0.06,18.54) | Lf-rTMS-Fp1 |  |  |  |  |  |  |  |  |  |  | 6.00 (0.58,61.84) |  |
| 10.15 (0.66,155.81) | 7.66 (0.31,190.64) | 5.71 (0.27,121.86) | 3.30 (0.14,79.64) | 1.60 (0.23,10.93) | 1.42 (0.20,10.00) | 1.38 (0.11,17.06) | Hf-dTMS-Fp1Fp2 |  |  |  |  |  |  |  |  |  | ***4.34 (1.71,11.00)** |  |
| 9.90 (0.32,307.20) | 7.47 (0.16,344.32) | 5.57 (0.14,225.80) | 3.22 (0.07,144.53) | 1.56 (0.09,26.52) | 1.38 (0.08,24.03) | 1.35 (0.05,35.28) | 0.98 (0.08,11.47) | c-tDCS-Fz + a-tDCS-extracephalic |  |  |  |  |  |  |  |  | 4.44 (0.45,43.54) |  |
| 11.23 (0.64,196.63) | 8.48 (0.30,236.06) | 6.32 (0.26,151.71) | 3.65 (0.13,98.71) | 1.77 (0.51,6.12) | 1.57 (0.19,13.24) | 1.53 (0.11,21.77) | 1.11 (0.23,5.33) | 1.13 (0.08,15.42) | Lf-rTMS-F4 |  |  |  |  |  |  |  | ***3.92 (1.11,13.90)** |  |
| 13.63 (0.84,221.92) | 10.29 (0.39,269.19) | 7.67 (0.34,172.49) | 4.43 (0.17,112.51) | 2.14 (0.29,15.94) | 1.90 (0.25,14.56) | 1.86 (0.14,24.43) | 1.34 (0.32,5.64) | 1.38 (0.11,17.28) | 1.21 (0.23,6.46) | Lf-rTMS-F5+F6 |  |  |  |  |  |  | ***3.23 (1.08,9.62)** |  |
| 15.71 (0.47,519.94) | 11.87 (0.24,578.99) | 8.84 (0.21,380.44) | 5.11 (0.11,243.13) | 2.47 (0.13,45.49) | 2.19 (0.12,41.19) | 2.14 (0.08,59.92) | 1.55 (0.12,19.90) | 1.59 (0.06,42.84) | 1.40 (0.09,20.69) | 1.15 (0.08,15.77) | cTBS-Fp1 |  |  |  |  |  | 2.80 (0.26,30.18) |  |
| 17.60 (0.46,667.52) | 13.29 (0.24,733.57) | 9.90 (0.20,483.94) | 5.72 (0.11,308.27) | 2.77 (0.13,59.95) | 2.45 (0.11,54.22) | 2.40 (0.07,77.44) | 1.73 (0.11,26.79) | 1.78 (0.06,55.45) | 1.57 (0.09,27.60) | 1.29 (0.08,21.15) | 1.12 (0.03,37.24) | c-tDCS-Fp1 + a-tDCS-O2 |  |  |  |  | 2.50 (0.19,32.80) |  |
| ***23.89 (1.30,440.49)** | 18.03 (0.62,525.06) | 13.44 (0.53,338.14) | 7.76 (0.27,219.66) | 3.75 (0.43,33.10) | 3.33 (0.37,30.18) | 3.26 (0.22,48.96) | 2.35 (0.45,12.43) | 2.41 (0.17,34.72) | 2.13 (0.33,13.83) | 1.75 (0.30,10.18) | 1.52 (0.10,23.75) | 1.36 (0.07,25.18) | cTBS-F3+F4 |  |  |  | 1.84 (0.46,7.32) |  |
| ***29.33 (1.12,766.33)** | 22.15 (0.56,875.01) | 16.51 (0.48,570.65) | 9.53 (0.25,366.91) | 4.61 (0.33,63.64) | 4.09 (0.29,57.74) | 4.00 (0.18,87.18) | 2.89 (0.31,26.57) | 2.96 (0.14,62.15) | 2.61 (0.24,28.18) | 2.15 (0.22,21.26) | 1.87 (0.08,42.09) | 1.67 (0.06,43.78) | 1.23 (0.11,14.10) | cTBS-Fp2 |  |  | 1.50 (0.20,11.24) |  |
| ***35.65 (1.52,833.77)** | 26.91 (0.75,964.27) | 20.06 (0.64,626.49) | 11.58 (0.33,404.06) | 5.60 (0.47,67.31) | 4.97 (0.40,61.15) | 4.86 (0.25,94.22) | 3.51 (0.45,27.35) | 3.60 (0.19,67.06) | 3.17 (0.34,29.35) | 2.61 (0.31,22.00) | 2.27 (0.11,45.55) | 2.03 (0.09,47.64) | 1.49 (0.15,14.75) | 1.22 (0.08,18.45) | Hf-rTMS-F3 | 1.00 (0.09,11.03) | 1.29 (0.16,10.45) |  |
| ***37.60 (1.33,1061.50)** | 28.39 (0.67,1201.72) | 21.16 (0.57,785.72) | 12.22 (0.30,504.16) | 5.91 (0.39,89.77) | 5.24 (0.34,81.39) | 5.13 (0.22,121.30) | 3.70 (0.36,38.12) | 3.80 (0.17,86.56) | 3.35 (0.28,40.13) | 2.76 (0.25,30.39) | 2.39 (0.10,58.51) | 2.14 (0.08,60.63) | 1.57 (0.12,20.03) | 1.28 (0.07,24.16) | 1.05 (0.14,7.72) | Hf-rTMS-F4 | 1.08 (0.06,19.31) |  |
| ***44.00 (3.38,573.41)** | ***33.22 (1.53,719.96)** | ***24.76 (1.34,456.87)** | 14.29 (0.68,300.37) | ***6.92 (1.28,37.25)** | ***6.14 (1.10,34.21)** | 6.00 (0.58,61.84) | ***4.33 (1.71,11.00)** | 4.44 (0.45,43.54) | ***3.92 (1.10,13.90)** | ***3.23 (1.08,9.62)** | 2.80 (0.26,30.18) | 2.50 (0.19,32.80) | 1.84 (0.46,7.32) | 1.50 (0.20,11.24) | 1.23 (0.20,7.69) | 1.17 (0.14,9.91) | Sham | 2.08 (0.30,14.29) |
| ***91.38 (3.69,2263.59)** | ***69.00 (1.83,2600.33)** | ***51.42 (1.56,1692.81)** | 29.69 (0.81,1090.02) | ***14.36 (1.11,185.50)** | 12.74 (0.96,168.41) | 12.46 (0.60,256.70) | ***9.00 (1.06,76.49)** | 9.23 (0.47,182.87) | 8.14 (0.81,81.56) | 6.70 (0.73,61.35) | 5.82 (0.27,124.01) | 5.19 (0.21,129.31) | 3.83 (0.36,40.90) | 3.12 (0.19,50.55) | 2.56 (0.18,36.51) | 2.43 (0.14,43.16) | 2.08 (0.30,14.25) | neuronavigated Lf-rTMS-F5+F6 |

Pairwise (upper-right portion) and network (lower-left portion) meta-analysis results are presented as estimate effect sizes for the outcome of response rate. Interventions are reported in order of mean ranking of treatment response, and outcomes are expressed as response odds ratio (OR) (95% confidence intervals). For the pairwise meta-analyses, OR of more than 1 indicate that the treatment specified in the row got better response than that specified in the column. For the network meta-analysis (NMA), OR of more than 1 indicate that the treatment specified in the column got better response than that specified in the row. Bold results marked with * indicate statistical significance.

Abbreviation: 95%CIs: 95% confidence intervals; ACC: anterior cingulate cortex; alpha-TMS-F3+F4: alpha EEG guided-TMS over F3+F4; a-tDCS-Fp3 + c-tDCS-F8: anode tDCS over Fp3 plus cathode tDCS over F8; a-tDCS-Fz + c-tDCS-extracephalic: anode tDCS over Fz plus cathode tDCS over extracephalic region; CBT: cognitive behavioral therapy; CGI-S: clinical global impression scale-severity; cTBS: continuous theta burst stimulation; cTBS-F3+F4: cTBS over F3+F4; cTBS-Fp1: cTBS over Fp1; cTBS-Fp2: cTBS over Fp2; c-tDCS-Fp1 + a-tDCS-O2: cathode tDCS over Fp1 plus anode tDCS over O2; c-tDCS-Fp2 + a-tDCS-F5: cathode tDCS over Fp2 plus anode tDCS over F5; c-tDCS-Fz + a-tDCS-extracephalic: cathode tDCS over Fz plus anode tDCS over extracephalic region; DLPFC: dorsolateral prefrontal cortex; dmPFC: dorsal medial prefrontal cortex; DSM: diagnostic and statistical manual of mental disorders; dTMS: deep TMS; EEG: electroencephalography; Hf-dTMS-Fp1Fp2: high frequency dTMS over Fp1Fp2; Hf-rTMS-F3: high frequency rTMS over F3; Hf-rTMS-F3+F4: high frequency rTMS over F3+F4; Hf-rTMS-F4: high frequency rTMS over F4; ICD: international classification of diseases; Lf-dTMS-Fp1Fp2: low frequency dTMS over Fp1Fp2; Lf-rTMS-F3: low frequency rTMS over F3; Lf-rTMS-F4: low frequency rTMS over F4; Lf-rTMS-F5+F6: low frequency rTMS over F5+F6; Lf-rTMS-Fp1: low frequency rTMS over Fp1; Lf-rTMS-Fp2: low frequency rTMS over Fp2; Lf-rTMS-Fz: low frequency rTMS over Fz; MD: mean difference; neuronavigated Lf-rTMS-F5+F6: neuronavigated low frequency rTMS over F5+F6; neuronavigated Lf-rTMS-F6: neuronavigated low frequency rTMS over F6; NIBS: noninvasive brain stimulation; NMA: network meta-analysis; OCD: obsessive-compulsive disorder; OFC: orbitofrontal cortex; OR: odds ratio; prLf-rTMS-F3: priming and low frequency rTMS over F3; RCT: randomized controlled trial; rTMS: repetitive TMS; Sham: sham control; SMD: standardized mean difference; SUCRA: surface under the cumulative ranking curve; tDCS: transcranial direct current stimulation; TMS: transcranial magnetic stimulation; YBOCS: Yale-Brown obsessive compulsive scale

**eTable 7: Inconsistency of different intervention:**

Part of design-by-treatment and loop inconsistency

| Inconsistency | chi2 | Prob>chi2 |
| --- | --- | --- |
| Changes in OCD overall severity | | |
| design-by-treatment | 16.22 | 0.0063 |
| loop | 0.71 | 0.3999 |
| Changes in OCD overall severity: subgroup of sham control | | |
| design-by-treatment | 16.22 | 0.0063 |
| loop | 0.05 | 0.8296 |
| Acceptability in aspect of drop-out rate | | |
| design-by-treatment | 0.12 | 0.7281 |
| loop | 0.53 | 0.4651 |
| Changes in clinical impression | | |
| design-by-treatment | 0.43 | 0.9797 |
| loop | 11.77 | 0.0006 |
| Changes in depression | | |
| design-by-treatment | 0.72 | 0.6989 |
| loop | 0.01 | 0.9176 |
| Changes in anxiety symptoms | | |
| design-by-treatment | 6.18 | 0.1033 |
| loop | 0.01 | 0.9407 |
| Response rate | | |
| design-by-treatment | 1.29 | 0.5235 |
| loop | 0.01 | 0.9377 |

Part of side-splitting inconsistency

Changes in OCD overall severity

| Side | symmetric | | nosymmetric | | Treatments used | |
| --- | --- | --- | --- | --- | --- | --- |
|  | P>z | tau | P>z | tau |  |  |
| A B * | 0 | 1.50534 | . | . | A: | Sham |
| A C * | 0.924 | 4.03243 | . | . | B: | Lf-dTMS-Fp1Fp2 |
| A D * | 0.907 | 4.0464 | . | . | C: | Lf-rTMS-F4 |
| A E | . | . | . | . | D: | Lf-rTMS-Fp2 |
| A F | . | . | . | . | E: | alpha-TMS-F3+F4 |
| A H * | 1 | 3.80529 | 1 | 3.80529 | F: | Lf-rTMS-F5+F6 |
| A I | . | . | . | . | G: | prLf-rTMS-F3 |
| A J | . | . | . | . | H: | Lf-rTMS-F3 |
| A K | . | . | . | . | I: | cTBS-F3+F4 |
| A L * | 0.997 | 3.80534 | 0.997 | 3.80534 | J: | cTBS-Fp2 |
| A M | . | . | . | . | K: | Hf-dTMS-Fp1Fp2 |
| A N | . | . | . | . | L: | c-tDCS-Fz + a-tDCS-extracephalic |
| A O | . | . | . | . | M: | cTBS-Fp1 |
| A P * | 0.487 | 3.92816 | 0.999 | 3.80528 | N: | c-tDCS-Fp2 + a-tDCS-F5 |
| A Q | . | . | . | . | O: | Lf-rTMS-Fz |
| A R | . | . | . | . | P: | Hf-rTMS-F4 |
| A T | . | . | . | . | Q: | neuronavigated Lf-rTMS-F6 |
| A U | . | . | . | . | R: | Hf-rTMS-F3+F4 |
| A V | . | . | . | . | S: | Hf-rTMS-F3 |
| A X | . | . | . | . | T: | neuronavigated Lf-rTMS-F5+F6 |
| B K * | 0 | 1.50534 | 0 | 1.50534 | U: | Lf-rTMS-Fp1 |
| C D | 0.793 | 4.01465 | 0.737 | 3.99598 | V: | a-tDCS-Fp3 + c-tDCS-F8 |
| C P | 0.474 | 3.92811 | 0.652 | 3.99558 | W: | a-tDCS-Fz + c-tDCS-extracephalic |
| G H * | 1 | 3.80529 | 1 | 3.80529 | X: | c-tDCS-Fp1 + a-tDCS-O2 |
| L W * | 0.997 | 3.80534 | . | . |  |  |
| P S * | 0.999 | 3.80528 | . | . |  |  |

Changes in OCD overall severity: subgroup of sham control

| Side | symmetric | | nosymmetric | | Treatments used | |
| --- | --- | --- | --- | --- | --- | --- |
|  | P>z | tau | P>z | tau |  |  |
| A B * | 0 | 1.505366 | . | . | A: | Sham |
| A C * | 0.924 | 4.03252 | . | . | B: | Lf-dTMS-Fp1Fp2 |
| A D * | 0.907 | 4.046486 | . | . | C: | Lf-rTMS-F4 |
| A E | . | . | . | . | D: | Lf-rTMS-Fp2 |
| A F | . | . | . | . | E: | alpha-TMS-F3+F4 |
| A G | . | . | . | . | F: | Lf-rTMS-F5+F6 |
| A H | . | . | . | . | G: | Lf-rTMS-Fp1 |
| A I | . | . | . | . | H: | Lf-rTMS-F3 |
| A J | . | . | . | . | I: | cTBS-F3+F4 |
| A K | . | . | . | . | J: | cTBS-Fp2 |
| A L | . | . | . | . | K: | Hf-dTMS-Fp1Fp2 |
| A M | . | . | . | . | L: | c-tDCS-Fz + a-tDCS-extracephalic |
| A N | . | . | . | . | M: | cTBS-Fp1 |
| A O | . | . | . | . | N: | c-tDCS-Fp2 + a-tDCS-F5 |
| A P * | 0.487 | 3.92825 | . | . | O: | Lf-rTMS-Fz |
| A Q | . | . | . | . | P: | Hf-rTMS-F4 |
| A R | . | . | . | . | Q: | neuronavigated Lf-rTMS-F6 |
| A S | . | . | . | . | R: | Hf-rTMS-F3+F4 |
| A T | . | . | . | . | S: | c-tDCS-Fp1 + a-tDCS-O2 |
| A U | . | . | . | . | T: | neuronavigated Lf-rTMS-F5+F6 |
| B K * | 0 | 1.505366 | 0 | 1.505366 | U: | a-tDCS-Fp3 + c-tDCS-F8 |
| C D | 0.793 | 4.014735 | 0.737 | 3.996068 |  |  |
| C P | 0.474 | 3.928196 | 0.652 | 3.995663 |  |  |

Acceptability in aspect of drop-out rate

| Side | symmetric | | nosymmetric | | Treatments used | |
| --- | --- | --- | --- | --- | --- | --- |
|  | P>z | tau | P>z | tau |  |  |
| A B * | 0.728 | 0.169392 | . | . | A: | Sham |
| A D | . | . | . | . | B: | Lf-dTMS-Fp1Fp2 |
| A E | . | . | . | . | C: | a-tDCS-Fz + c-tDCS-extracephalic |
| A F | . | . | . | . | D: | Lf-rTMS-Fp2 |
| A G | . | . | . | . | E: | a-tDCS-Fp3 + c-tDCS-F8 |
| A H | . | . | . | . | F: | Lf-rTMS-F5+F6 |
| A I | . | . | . | . | G: | Lf-rTMS-Fz |
| A J | . | . | . | . | H: | Lf-rTMS-F3 |
| A K | . | . | . | . | I: | cTBS-F3+F4 |
| A L * | 0.999 | 5.36E-08 | 0.999 | 5.36E-08 | J: | cTBS-Fp2 |
| A M | . | . | . | . | K: | Hf-dTMS-Fp1Fp2 |
| A N | . | . | . | . | L: | c-tDCS-Fz + a-tDCS-extracephalic |
| B K * | 0.728 | 0.169416 | 0.728 | 0.169348 | M: | neuronavigated Lf-rTMS-F5+F6 |
| C L * | 0.999 | 3.25E-08 | 0.999 | 2.62E-09 | N: | Hf-rTMS-F4 |

Changes in clinical impression

| Side | symmetric | | nosymmetric | | Treatments used | |
| --- | --- | --- | --- | --- | --- | --- |
|  | P>z | tau | P>z | tau |  |  |
| A B | . | . | . | . | A: | Sham |
| A C * | 0.736 | 1.10E-07 | . | . | B: | c-tDCS-Fp1 + a-tDCS-O2 |
| A D * | 0.713 | 3.39E-08 | . | . | C: | Lf-rTMS-F4 |
| A E | . | . | . | . | D: | Lf-rTMS-Fp2 |
| A F | . | . | . | . | E: | alpha-TMS-F3+F4 |
| A H * | 0.999 | 7.39E-10 | 0.999 | 7.39E-10 | F: | Lf-rTMS-F5+F6 |
| A I | . | . | . | . | G: | prLf-rTMS-F3 |
| A J | . | . | . | . | H: | Lf-rTMS-F3 |
| A K | . | . | . | . | I: | neuronavigated Lf-rTMS-F5+F6 |
| A L * | 0.826 | 2.77E-12 | . | . | J: | Hf-rTMS-F3+F4 |
| A M | . | . | . | . | K: | Lf-rTMS-Fz |
| C D | 0.611 | 5.68E-09 | 0.628 | 2.18E-08 | L: | Hf-rTMS-F4 |
| C L | 0.849 | 3.89E-08 | 0.922 | 4.79E-10 | M: | cTBS-Fp1 |
| G H * | 0.999 | 3.44E-08 | 0.999 | 4.16E-13 |  |  |

Changes in depression

| Side | symmetric | | nosymmetric | | Treatments used | |
| --- | --- | --- | --- | --- | --- | --- |
|  | P>z | tau | P>z | tau |  |  |
| A B | . | . | . | . | A: | Sham |
| A C * | 0.824 | 7.04E-12 | . | . | B: | c-tDCS-Fp1 + a-tDCS-O2 |
| A D * | 0.4 | 3.43E-09 | . | . | C: | Lf-rTMS-F4 |
| A E | . | . | . | . | D: | Lf-rTMS-Fp2 |
| A F | . | . | . | . | E: | alpha-TMS-F3+F4 |
| A G | . | . | . | . | F: | Lf-rTMS-F5+F6 |
| A H | . | . | . | . | G: | Lf-rTMS-Fz |
| A I | . | . | . | . | H: | neuronavigated Lf-rTMS-F5+F6 |
| A J | . | . | . | . | I: | cTBS-F3+F4 |
| A L | . | . | . | . | J: | cTBS-Fp2 |
| A M | . | . | . | . | K: | Hf-rTMS-F3 |
| A N * | 0.999 | 2.02E-08 | 0.999 | 2.02E-08 | L: | c-tDCS-Fz + a-tDCS-extracephalic |
| C D | 0.43 | 7.06E-09 | 0.824 | 8.28E-09 | M: | cTBS-Fp1 |
| K N * | 0.999 | 4.14E-09 | 0.999 | 2.40E-10 | N: | Hf-rTMS-F4 |

Changes in anxiety symptoms

| Side | symmetric | | nosymmetric | | Treatments used | |
| --- | --- | --- | --- | --- | --- | --- |
|  | P>z | tau | P>z | tau |  |  |
| A B | . | . | . | . | A: | Sham |
| A C * | 0.186 | 0.208386 | . | . | B: | c-tDCS-Fp1 + a-tDCS-O2 |
| A D * | 0.406 | 0.285425 | . | . | C: | Lf-rTMS-F4 |
| A E | . | . | . | . | D: | Lf-rTMS-Fp2 |
| A F | . | . | . | . | E: | alpha-TMS-F3+F4 |
| A H * | 0.999 | 0.252894 | 0.999 | 0.252894 | F: | Lf-rTMS-F5+F6 |
| A I | . | . | . | . | G: | prLf-rTMS-F3 |
| A J | . | . | . | . | H: | Lf-rTMS-F3 |
| A L | . | . | . | . | I: | cTBS-F3+F4 |
| A M | . | . | . | . | J: | cTBS-Fp2 |
| A N | . | . | . | . | K: | Hf-rTMS-F3 |
| A O | . | . | . | . | L: | c-tDCS-Fz + a-tDCS-extracephalic |
| A P * | 0.247 | 0.255266 | 1 | 0.252894 | M: | cTBS-Fp1 |
| C D * | 0.406 | 0.285426 | . | . | N: | neuronavigated Lf-rTMS-F5+F6 |
| C P | 0.104 | 0.161624 | 0.186 | 0.208386 | O: | Lf-rTMS-Fz |
| G H * | 0.999 | 0.252894 | 0.999 | 0.252894 | P: | Hf-rTMS-F4 |
| K P * | 1 | 0.252892 | 1 | 0.252894 |  |  |

Response rate

| Side | symmetric | | nosymmetric | | Treatments used | |
| --- | --- | --- | --- | --- | --- | --- |
|  | P>z | tau | P>z | tau |  |  |
| A B | . | . | . | . | A: | Sham |
| A C | . | . | . | . | B: | Lf-rTMS-Fp1 |
| A D * | 0.256 | 1.51E-08 | . | . | C: | Lf-rTMS-F4 |
| A E | . | . | . | . | D: | Lf-rTMS-Fp2 |
| A F | . | . | . | . | E: | alpha-TMS-F3+F4 |
| A G | . | . | . | . | F: | Lf-rTMS-F5+F6 |
| A H | . | . | . | . | G: | c-tDCS-Fp1 + a-tDCS-O2 |
| A I | . | . | . | . | H: | neuronavigated Lf-rTMS-F5+F6 |
| A J | . | . | . | . | I: | cTBS-F3+F4 |
| A K | . | . | . | . | J: | cTBS-Fp2 |
| A L | . | . | . | . | K: | Hf-dTMS-Fp1Fp2 |
| A M | . | . | . | . | L: | c-tDCS-Fz + a-tDCS-extracephalic |
| A N | . | . | . | . | M: | cTBS-Fp1 |
| A O | . | . | . | . | N: | c-tDCS-Fp2 + a-tDCS-F5 |
| A P | 0.938 | 8.16E-09 | 0.938 | 8.16E-09 | O: | Lf-rTMS-Fz |
| A Q | . | . | . | . | P: | Hf-rTMS-F4 |
| A R | . | . | . | . | Q: | neuronavigated Lf-rTMS-F6 |
| A S | 0.938 | 1.08E-08 | 0.938 | 1.08E-08 | R: | Hf-rTMS-F3+F4 |
| C D * | 0.256 | 8.42E-09 | . | . | S: | Hf-rTMS-F3 |
| P S | 0.938 | 2.12E-08 | 0.938 | 2.79E-09 |  |  |

Abbreviation: 95%CIs: 95% confidence intervals; ACC: anterior cingulate cortex; alpha-TMS-F3+F4: alpha EEG guided-TMS over F3+F4; a-tDCS-Fp3 + c-tDCS-F8: anode tDCS over Fp3 plus cathode tDCS over F8; a-tDCS-Fz + c-tDCS-extracephalic: anode tDCS over Fz plus cathode tDCS over extracephalic region; CBT: cognitive behavioral therapy; CGI-S: clinical global impression scale-severity; cTBS: continuous theta burst stimulation; cTBS-F3+F4: cTBS over F3+F4; cTBS-Fp1: cTBS over Fp1; cTBS-Fp2: cTBS over Fp2; c-tDCS-Fp1 + a-tDCS-O2: cathode tDCS over Fp1 plus anode tDCS over O2; c-tDCS-Fp2 + a-tDCS-F5: cathode tDCS over Fp2 plus anode tDCS over F5; c-tDCS-Fz + a-tDCS-extracephalic: cathode tDCS over Fz plus anode tDCS over extracephalic region; DLPFC: dorsolateral prefrontal cortex; dmPFC: dorsal medial prefrontal cortex; DSM: diagnostic and statistical manual of mental disorders; dTMS: deep TMS; EEG: electroencephalography; Hf-dTMS-Fp1Fp2: high frequency dTMS over Fp1Fp2; Hf-rTMS-F3: high frequency rTMS over F3; Hf-rTMS-F3+F4: high frequency rTMS over F3+F4; Hf-rTMS-F4: high frequency rTMS over F4; ICD: international classification of diseases; Lf-dTMS-Fp1Fp2: low frequency dTMS over Fp1Fp2; Lf-rTMS-F3: low frequency rTMS over F3; Lf-rTMS-F4: low frequency rTMS over F4; Lf-rTMS-F5+F6: low frequency rTMS over F5+F6; Lf-rTMS-Fp1: low frequency rTMS over Fp1; Lf-rTMS-Fp2: low frequency rTMS over Fp2; Lf-rTMS-Fz: low frequency rTMS over Fz; MD: mean difference; neuronavigated Lf-rTMS-F5+F6: neuronavigated low frequency rTMS over F5+F6; neuronavigated Lf-rTMS-F6: neuronavigated low frequency rTMS over F6; NIBS: noninvasive brain stimulation; NMA: network meta-analysis; OCD: obsessive-compulsive disorder; OFC: orbitofrontal cortex; OR: odds ratio; prLf-rTMS-F3: priming and low frequency rTMS over F3; RCT: randomized controlled trial; rTMS: repetitive TMS; Sham: sham control; SMD: standardized mean difference; SUCRA: surface under the cumulative ranking curve; tDCS: transcranial direct current stimulation; TMS: transcranial magnetic stimulation; YBOCS: Yale-Brown obsessive compulsive scale

**eTable 8: Estimated between-studies standard deviations of different outcome**

| Outcome | Estimated between-studies standard deviation |
| --- | --- |
| Changes in OCD overall severity | 3.8052813 |
| Changes in OCD overall severity: subgroup of sham control | 3.8053576 |
| Acceptability in aspect of drop-out rate | 3.789e-08 |
| Changes in clinical impression | 6.279e-09 |
| Changes in depression | 5.873e-09 |
| Changes in anxiety symptoms | 0.25289341 |
| Response rate | 3.479e-08 |

**eTable 9: Heterogeneity among the experiment arms in primary outcome: changes in OCD overall severity**

|  |  |  | Heterogeneity statistic | degrees of freedom | *p* | *I* squared | Tau-squared | Treatments used | |
| --- | --- | --- | --- | --- | --- | --- | --- | --- | --- |
| C | - | A | 4.63 | 4 | 0.328 | 13.60% | 1.346 | A: | Sham |
| D | - | A | 0.27 | 1 | 0.606 | 0.00% | 0 | B: | Lf-dTMS-Fp1Fp2 |
| D | - | C | 0 | 0 | . | .% | 0 | C: | Lf-rTMS-F4 |
| E | - | A | 0 | 0 | . | .% | 0 | D: | Lf-rTMS-Fp2 |
| F | - | A | 1.46 | 2 | 0.483 | 0.00% | 0 | E: | alpha-TMS-F3+F4 |
| H | - | G | 0 | 0 | . | .% | 0 | F: | Lf-rTMS-F5+F6 |
| I | - | A | 0 | 0 | . | .% | 0 | G: | prLf-rTMS-F3 |
| J | - | A | 0 | 0 | . | .% | 0 | H: | Lf-rTMS-F3 |
| H | - | A | 8.6 | 1 | 0.003 | 88.40% | 29.6376 | I: | cTBS-F3+F4 |
| K | - | A | 23.29 | 1 | 0 | 95.70% | 185.8672 | J: | cTBS-Fp2 |
| L | - | A | 0 | 0 | . | .% | 0 | K: | Hf-dTMS-Fp1Fp2 |
| M | - | A | 0 | 0 | . | .% | 0 | L: | c-tDCS-Fz + a-tDCS-extracephalic |
| N | - | A | 0 | 0 | . | .% | 0 | M: | cTBS-Fp1 |
| O | - | A | 1.92 | 3 | 0.588 | 0.00% | 0 | N: | c-tDCS-Fp2 + a-tDCS-F5 |
| P | - | A | 0.16 | 1 | 0.693 | 0.00% | 0 | O: | Lf-rTMS-Fz |
| Q | - | A | 0 | 0 | . | .% | 0 | P: | Hf-rTMS-F4 |
| R | - | A | 0 | 0 | . | .% | 0 | Q: | neuronavigated Lf-rTMS-F6 |
| P | - | C | 0 | 0 | . | .% | 0 | R: | Hf-rTMS-F3+F4 |
| T | - | A | 0 | 0 | . | .% | 0 | S: | Hf-rTMS-F3 |
| S | - | P | 0 | 0 | . | .% | 0 | T: | neuronavigated Lf-rTMS-F5+F6 |
| V | - | A | 0 | 0 | . | .% | 0 | U: | Lf-rTMS-Fp1 |
| W | - | L | 0 | 0 | . | .% | 0 | V: | a-tDCS-Fp3 + c-tDCS-F8 |
| X | - | A | 0 | 0 | . | .% | 0 | W: | a-tDCS-Fz + c-tDCS-extracephalic |
| U | - | A | 0 | 0 | . | .% | 0 | X: | c-tDCS-Fp1 + a-tDCS-O2 |
| B | - | A | 0 | 0 | . | .% | 0 |  |  |
| K | - | B | 0 | 0 | . | .% | 0 |  |  |

Abbreviation: 95%CIs: 95% confidence intervals; ACC: anterior cingulate cortex; alpha-TMS-F3+F4: alpha EEG guided-TMS over F3+F4; a-tDCS-Fp3 + c-tDCS-F8: anode tDCS over Fp3 plus cathode tDCS over F8; a-tDCS-Fz + c-tDCS-extracephalic: anode tDCS over Fz plus cathode tDCS over extracephalic region; CBT: cognitive behavioral therapy; CGI-S: clinical global impression scale-severity; cTBS: continuous theta burst stimulation; cTBS-F3+F4: cTBS over F3+F4; cTBS-Fp1: cTBS over Fp1; cTBS-Fp2: cTBS over Fp2; c-tDCS-Fp1 + a-tDCS-O2: cathode tDCS over Fp1 plus anode tDCS over O2; c-tDCS-Fp2 + a-tDCS-F5: cathode tDCS over Fp2 plus anode tDCS over F5; c-tDCS-Fz + a-tDCS-extracephalic: cathode tDCS over Fz plus anode tDCS over extracephalic region; DLPFC: dorsolateral prefrontal cortex; dmPFC: dorsal medial prefrontal cortex; DSM: diagnostic and statistical manual of mental disorders; dTMS: deep TMS; EEG: electroencephalography; Hf-dTMS-Fp1Fp2: high frequency dTMS over Fp1Fp2; Hf-rTMS-F3: high frequency rTMS over F3; Hf-rTMS-F3+F4: high frequency rTMS over F3+F4; Hf-rTMS-F4: high frequency rTMS over F4; ICD: international classification of diseases; Lf-dTMS-Fp1Fp2: low frequency dTMS over Fp1Fp2; Lf-rTMS-F3: low frequency rTMS over F3; Lf-rTMS-F4: low frequency rTMS over F4; Lf-rTMS-F5+F6: low frequency rTMS over F5+F6; Lf-rTMS-Fp1: low frequency rTMS over Fp1; Lf-rTMS-Fp2: low frequency rTMS over Fp2; Lf-rTMS-Fz: low frequency rTMS over Fz; MD: mean difference; neuronavigated Lf-rTMS-F5+F6: neuronavigated low frequency rTMS over F5+F6; neuronavigated Lf-rTMS-F6: neuronavigated low frequency rTMS over F6; NIBS: noninvasive brain stimulation; NMA: network meta-analysis; OCD: obsessive-compulsive disorder; OFC: orbitofrontal cortex; OR: odds ratio; prLf-rTMS-F3: priming and low frequency rTMS over F3; RCT: randomized controlled trial; rTMS: repetitive TMS; Sham: sham control; SMD: standardized mean difference; SUCRA: surface under the cumulative ranking curve; tDCS: transcranial direct current stimulation; TMS: transcranial magnetic stimulation; YBOCS: Yale-Brown obsessive compulsive scale

**eTable 10: GRADE ratings for primary outcome: changes in OCD overall severity**

| Comparisons | GRADE | | | | | |
| --- | --- | --- | --- | --- | --- | --- |
|  | Direct | | Indirect | | Network meta-analysis | |
|  | MD (95%CIs) | The final rating of direct evidence | Co-efficiency (Standard error) | The final rating of indirect evidence | MD (95%CIs) | Overall quality of evidence |
| Hf-rTMS-F3+F4 vs Hf-dTMS-Fp1Fp2 |  |  |  |  | -1.07 (-13.09,10.95) | ⨁◯◯◯ Very low |
| Hf-rTMS-F3+F4 vs c-tDCS-Fp2 + a-tDCS-F5 |  |  |  |  | -0.79 (-19.67,18.09) | ⨁◯◯◯ Very low |
| Hf-rTMS-F3+F4 vs Lf-rTMS-F4 |  |  |  |  | -6.11 (-16.92,4.71) | ⨁◯◯◯ Very low |
| Hf-rTMS-F3+F4 vs Lf-dTMS-Fp1Fp2 |  |  |  |  | -5.46 (-19.61,8.68) | ⨁◯◯◯ Very low |
| Hf-rTMS-F3+F4 vs Lf-rTMS-F5+F6 |  |  |  |  | -6.44 (-17.70,4.81) | ⨁◯◯◯ Very low |
| Hf-rTMS-F3+F4 vs Lf-rTMS-Fz |  |  |  |  | -7.65 (-18.49,3.20) | ⨁◯◯◯ Very low |
| Hf-rTMS-F3+F4 vs Lf-rTMS-Fp1 |  |  |  |  | -7.73 (-20.63,5.17) | ⨁◯◯◯ Very low |
| Hf-rTMS-F3+F4 vs neuronavigated Lf-rTMS-F6 |  |  |  |  | -8.03 (-21.01,4.95) | ⨁◯◯◯ Very low |
| Hf-rTMS-F3+F4 vs c-tDCS-Fp1 + a-tDCS-O2 |  |  |  |  | -8.44 (-21.87,4.99) | ⨁◯◯◯ Very low |
| Hf-rTMS-F3+F4 vs c-tDCS-Fz + a-tDCS-extracephalic |  |  |  |  | -8.50 (-21.54,4.54) | ⨁◯◯◯ Very low |
| Hf-rTMS-F3+F4 vs Lf-rTMS-Fp2 |  |  |  |  | -8.71 (-20.60,3.18) | ⨁◯◯◯ Very low |
| Hf-rTMS-F3+F4 vs alpha-TMS-F3+F4 |  |  |  |  | -8.91 (-21.96,4.14) | ⨁◯◯◯ Very low |
| Hf-rTMS-F3+F4 vs cTBS-F3+F4 |  |  |  |  | -9.34 (-22.32,3.64) | ⨁◯◯◯ Very low |
| Hf-rTMS-F3+F4 vs cTBS-Fp2 |  |  |  |  | -9.49 (-22.36,3.38) | ⨁◯◯◯ Very low |
| Hf-rTMS-F3+F4 vs cTBS-Fp1 |  |  |  |  | -10.30 (-22.87,2.26) | ⨁⨁◯◯ Low |
| Hf-rTMS-F3+F4 vs prLf-rTMS-F3 |  |  |  |  | -10.72 (-24.62,3.19) | ⨁⨁◯◯ Low |
| Hf-rTMS-F3+F4 vs Lf-rTMS-F3 |  |  |  |  | -10.39 (-21.98,1.21) | ⨁⨁◯◯ Low |
| Hf-rTMS-F3+F4 vs Hf-rTMS-F4 |  |  |  |  | -10.97 (-22.67,0.74) | ⨁⨁◯◯ Low |
| Hf-rTMS-F3+F4 vs Sham | ***-10.81 (-17.46,-4.16)** | ⨁⨁⨁◯ Medium |  |  | ***-10.81 (-20.80,-0.82)** | ⨁⨁⨁◯ Medium |
| Hf-rTMS-F3+F4 vs a-tDCS-Fp3 + c-tDCS-F8 |  |  |  |  | -11.81 (-25.33,1.71) | ⨁⨁◯◯ Low |
| Hf-rTMS-F3+F4 vs neuronavigated Lf-rTMS-F5+F6 |  |  |  |  | -12.01 (-24.90,0.88) | ⨁⨁◯◯ Low |
| Hf-rTMS-F3+F4 vs Hf-rTMS-F3 |  |  |  |  | -15.47 (-31.18,0.24) | ⨁⨁◯◯ Low |
| Hf-rTMS-F3+F4 vs a-tDCS-Fz + c-tDCS-extracephalic |  |  |  |  | ***-15.95 (-31.05,-0.85)** | ⨁⨁⨁◯ Medium |
| Hf-dTMS-Fp1Fp2 vs c-tDCS-Fp2 + a-tDCS-F5 |  |  |  |  | 0.28 (-17.08,17.64) | ⨁◯◯◯ Very low |
| Hf-dTMS-Fp1Fp2 vs Lf-rTMS-F4 |  |  |  |  | -5.04 (-12.91,2.84) | ⨁◯◯◯ Very low |
| Hf-dTMS-Fp1Fp2 vs Lf-dTMS-Fp1Fp2 | ***-11.78 (-20.56,-3.00)** | ⨁⨁⨁◯ Medium | 27.64 (7.95) | ⨁⨁◯◯ Low | -4.40 (-14.96,6.17) | ⨁⨁⨁◯ Medium |
| Hf-dTMS-Fp1Fp2 vs Lf-rTMS-F5+F6 |  |  |  |  | -5.37 (-13.85,3.10) | ⨁◯◯◯ Very low |
| Hf-dTMS-Fp1Fp2 vs Lf-rTMS-Fz |  |  |  |  | -6.58 (-14.39,1.24) | ⨁◯◯◯ Very low |
| Hf-dTMS-Fp1Fp2 vs Lf-rTMS-Fp1 |  |  |  |  | -6.66 (-17.20,3.88) | ⨁◯◯◯ Very low |
| Hf-dTMS-Fp1Fp2 vs neuronavigated Lf-rTMS-F6 |  |  |  |  | -6.96 (-17.60,3.68) | ⨁◯◯◯ Very low |
| Hf-dTMS-Fp1Fp2 vs c-tDCS-Fp1 + a-tDCS-O2 |  |  |  |  | -7.37 (-18.56,3.82) | ⨁◯◯◯ Very low |
| Hf-dTMS-Fp1Fp2 vs c-tDCS-Fz + a-tDCS-extracephalic |  |  |  |  | -7.43 (-18.14,3.28) | ⨁◯◯◯ Very low |
| Hf-dTMS-Fp1Fp2 vs Lf-rTMS-Fp2 |  |  |  |  | -7.64 (-16.93,1.66) | ⨁◯◯◯ Very low |
| Hf-dTMS-Fp1Fp2 vs alpha-TMS-F3+F4 |  |  |  |  | -7.84 (-18.57,2.89) | ⨁◯◯◯ Very low |
| Hf-dTMS-Fp1Fp2 vs cTBS-F3+F4 |  |  |  |  | -8.27 (-18.92,2.37) | ⨁◯◯◯ Very low |
| Hf-dTMS-Fp1Fp2 vs cTBS-Fp2 |  |  |  |  | -8.42 (-18.93,2.08) | ⨁◯◯◯ Very low |
| Hf-dTMS-Fp1Fp2 vs cTBS-Fp1 |  |  |  |  | -9.24 (-19.36,0.89) | ⨁◯◯◯ Very low |
| Hf-dTMS-Fp1Fp2 vs prLf-rTMS-F3 |  |  |  |  | -9.65 (-21.52,2.23) | ⨁◯◯◯ Very low |
| Hf-dTMS-Fp1Fp2 vs Lf-rTMS-F3 |  |  |  |  | ***-9.32 (-18.37,-0.26)** | ⨁⨁⨁◯ Medium |
| Hf-dTMS-Fp1Fp2 vs Hf-rTMS-F4 |  |  |  |  | ***-9.90 (-18.92,-0.88)** | ⨁⨁⨁◯ Medium |
| Hf-dTMS-Fp1Fp2 vs Sham | -11.96 (-31.26,7.35) | ⨁◯◯◯ Very low |  |  | ***-9.74 (-16.42,-3.06)** | ⨁⨁⨁◯ Medium |
| Hf-dTMS-Fp1Fp2 vs a-tDCS-Fp3 + c-tDCS-F8 |  |  |  |  | -10.74 (-22.03,0.55) | ⨁⨁◯◯ Low |
| Hf-dTMS-Fp1Fp2 vs neuronavigated Lf-rTMS-F5+F6 |  |  |  |  | ***-10.94 (-21.47,-0.42)** | ⨁⨁⨁◯ Medium |
| Hf-dTMS-Fp1Fp2 vs Hf-rTMS-F3 |  |  |  |  | ***-14.40 (-28.22,-0.57)** | ⨁⨁⨁◯ Medium |
| Hf-dTMS-Fp1Fp2 vs a-tDCS-Fz + c-tDCS-extracephalic |  |  |  |  | ***-14.88 (-28.03,-1.74)** | ⨁⨁⨁◯ Medium |
| c-tDCS-Fp2 + a-tDCS-F5 vs Lf-rTMS-F4 |  |  |  |  | -5.32 (-21.86,11.23) | ⨁◯◯◯ Very low |
| c-tDCS-Fp2 + a-tDCS-F5 vs Lf-dTMS-Fp1Fp2 |  |  |  |  | -4.67 (-23.57,14.22) | ⨁◯◯◯ Very low |
| c-tDCS-Fp2 + a-tDCS-F5 vs Lf-rTMS-F5+F6 |  |  |  |  | -5.65 (-22.49,11.19) | ⨁◯◯◯ Very low |
| c-tDCS-Fp2 + a-tDCS-F5 vs Lf-rTMS-Fz |  |  |  |  | -6.86 (-23.42,9.71) | ⨁◯◯◯ Very low |
| c-tDCS-Fp2 + a-tDCS-F5 vs Lf-rTMS-Fp1 |  |  |  |  | -6.94 (-24.92,11.04) | ⨁◯◯◯ Very low |
| c-tDCS-Fp2 + a-tDCS-F5 vs neuronavigated Lf-rTMS-F6 |  |  |  |  | -7.24 (-25.28,10.80) | ⨁◯◯◯ Very low |
| c-tDCS-Fp2 + a-tDCS-F5 vs c-tDCS-Fp1 + a-tDCS-O2 |  |  |  |  | -7.65 (-26.01,10.71) | ⨁◯◯◯ Very low |
| c-tDCS-Fp2 + a-tDCS-F5 vs c-tDCS-Fz + a-tDCS-extracephalic |  |  |  |  | -7.71 (-25.79,10.37) | ⨁◯◯◯ Very low |
| c-tDCS-Fp2 + a-tDCS-F5 vs Lf-rTMS-Fp2 |  |  |  |  | -7.92 (-25.19,9.35) | ⨁◯◯◯ Very low |
| c-tDCS-Fp2 + a-tDCS-F5 vs alpha-TMS-F3+F4 |  |  |  |  | -8.12 (-26.21,9.97) | ⨁◯◯◯ Very low |
| c-tDCS-Fp2 + a-tDCS-F5 vs cTBS-F3+F4 |  |  |  |  | -8.55 (-26.59,9.49) | ⨁◯◯◯ Very low |
| c-tDCS-Fp2 + a-tDCS-F5 vs cTBS-Fp2 |  |  |  |  | -8.70 (-26.66,9.26) | ⨁◯◯◯ Very low |
| c-tDCS-Fp2 + a-tDCS-F5 vs cTBS-Fp1 |  |  |  |  | -9.51 (-27.25,8.22) | ⨁◯◯◯ Very low |
| c-tDCS-Fp2 + a-tDCS-F5 vs prLf-rTMS-F3 |  |  |  |  | -9.93 (-28.64,8.79) | ⨁◯◯◯ Very low |
| c-tDCS-Fp2 + a-tDCS-F5 vs Lf-rTMS-F3 |  |  |  |  | -9.60 (-26.66,7.47) | ⨁◯◯◯ Very low |
| c-tDCS-Fp2 + a-tDCS-F5 vs Hf-rTMS-F4 |  |  |  |  | -10.18 (-27.32,6.96) | ⨁⨁◯◯ Low |
| c-tDCS-Fp2 + a-tDCS-F5 vs Sham | -10.02 (-24.20,4.16) | ⨁◯◯◯ Very low |  |  | -10.02 (-26.04,6.00) | ⨁⨁⨁◯ Medium |
| c-tDCS-Fp2 + a-tDCS-F5 vs a-tDCS-Fp3 + c-tDCS-F8 |  |  |  |  | -11.02 (-29.45,7.41) | ⨁⨁◯◯ Low |
| c-tDCS-Fp2 + a-tDCS-F5 vs neuronavigated Lf-rTMS-F5+F6 |  |  |  |  | -11.22 (-29.19,6.75) | ⨁⨁◯◯ Low |
| c-tDCS-Fp2 + a-tDCS-F5 vs Hf-rTMS-F3 |  |  |  |  | -14.68 (-34.77,5.41) | ⨁⨁◯◯ Low |
| c-tDCS-Fp2 + a-tDCS-F5 vs a-tDCS-Fz + c-tDCS-extracephalic |  |  |  |  | -15.16 (-34.78,4.45) | ⨁⨁◯◯ Low |
| Lf-rTMS-F4 vs Lf-dTMS-Fp1Fp2 |  |  |  |  | 0.64 (-10.19,11.47) | ⨁◯◯◯ Very low |
| Lf-rTMS-F4 vs Lf-rTMS-F5+F6 |  |  |  |  | -0.34 (-6.96,6.29) | ⨁◯◯◯ Very low |
| Lf-rTMS-F4 vs Lf-rTMS-Fz |  |  |  |  | -1.54 (-7.45,4.37) | ⨁◯◯◯ Very low |
| Lf-rTMS-F4 vs Lf-rTMS-Fp1 |  |  |  |  | -1.62 (-10.76,7.52) | ⨁◯◯◯ Very low |
| Lf-rTMS-F4 vs neuronavigated Lf-rTMS-F6 |  |  |  |  | -1.92 (-11.18,7.33) | ⨁◯◯◯ Very low |
| Lf-rTMS-F4 vs c-tDCS-Fp1 + a-tDCS-O2 |  |  |  |  | -2.33 (-12.22,7.55) | ⨁◯◯◯ Very low |
| Lf-rTMS-F4 vs c-tDCS-Fz + a-tDCS-extracephalic |  |  |  |  | -2.39 (-11.73,6.94) | ⨁◯◯◯ Very low |
| Lf-rTMS-F4 vs Lf-rTMS-Fp2 | -1.70 (-7.51,4.11) | ⨁◯◯◯ Very low | 3.65 (5.43) | ⨁⨁◯◯ Low | -2.60 (-9.60,4.40) | ⨁⨁⨁◯ Medium |
| Lf-rTMS-F4 vs alpha-TMS-F3+F4 |  |  |  |  | -2.80 (-12.16,6.55) | ⨁◯◯◯ Very low |
| Lf-rTMS-F4 vs cTBS-F3+F4 |  |  |  |  | -3.24 (-12.50,6.02) | ⨁◯◯◯ Very low |
| Lf-rTMS-F4 vs cTBS-Fp2 |  |  |  |  | -3.38 (-12.49,5.72) | ⨁◯◯◯ Very low |
| Lf-rTMS-F4 vs cTBS-Fp1 |  |  |  |  | -4.20 (-12.86,4.46) | ⨁◯◯◯ Very low |
| Lf-rTMS-F4 vs prLf-rTMS-F3 |  |  |  |  | -4.61 (-15.12,5.90) | ⨁◯◯◯ Very low |
| Lf-rTMS-F4 vs Lf-rTMS-F3 |  |  |  |  | -4.28 (-11.45,2.89) | ⨁◯◯◯ Very low |
| Lf-rTMS-F4 vs Hf-rTMS-F4 | ***-7.10 (-11.86,-2.34)** | ⨁⨁◯◯ Low | 2.02 (5.23) | ⨁⨁◯◯ Low | -4.86 (-11.43,1.71) | ⨁⨁⨁◯ Medium |
| Lf-rTMS-F4 vs Sham | ***-4.77 (-7.52,-2.03)** | ⨁⨁◯◯ Low | -5.56 (9.41) | ⨁⨁◯◯ Low | ***-4.70 (-8.84,-0.57)** | ⨁⨁⨁⨁ High |
| Lf-rTMS-F4 vs a-tDCS-Fp3 + c-tDCS-F8 |  |  |  |  | -5.70 (-15.71,4.30) | ⨁◯◯◯ Very low |
| Lf-rTMS-F4 vs neuronavigated Lf-rTMS-F5+F6 |  |  |  |  | -5.90 (-15.03,3.22) | ⨁◯◯◯ Very low |
| Lf-rTMS-F4 vs Hf-rTMS-F3 |  |  |  |  | -9.36 (-21.73,3.01) | ⨁◯◯◯ Very low |
| Lf-rTMS-F4 vs a-tDCS-Fz + c-tDCS-extracephalic |  |  |  |  | -9.85 (-21.90,2.20) | ⨁◯◯◯ Very low |
| Lf-dTMS-Fp1Fp2 vs Lf-rTMS-F5+F6 |  |  |  |  | -0.98 (-12.25,10.29) | ⨁◯◯◯ Very low |
| Lf-dTMS-Fp1Fp2 vs Lf-rTMS-Fz |  |  |  |  | -2.18 (-13.04,8.67) | ⨁◯◯◯ Very low |
| Lf-dTMS-Fp1Fp2 vs Lf-rTMS-Fp1 |  |  |  |  | -2.27 (-15.17,10.64) | ⨁◯◯◯ Very low |
| Lf-dTMS-Fp1Fp2 vs neuronavigated Lf-rTMS-F6 |  |  |  |  | -2.57 (-15.56,10.42) | ⨁◯◯◯ Very low |
| Lf-dTMS-Fp1Fp2 vs c-tDCS-Fp1 + a-tDCS-O2 |  |  |  |  | -2.98 (-16.42,10.47) | ⨁◯◯◯ Very low |
| Lf-dTMS-Fp1Fp2 vs c-tDCS-Fz + a-tDCS-extracephalic |  |  |  |  | -3.04 (-16.09,10.01) | ⨁◯◯◯ Very low |
| Lf-dTMS-Fp1Fp2 vs Lf-rTMS-Fp2 |  |  |  |  | -3.24 (-15.15,8.66) | ⨁◯◯◯ Very low |
| Lf-dTMS-Fp1Fp2 vs alpha-TMS-F3+F4 |  |  |  |  | -3.45 (-16.51,9.62) | ⨁◯◯◯ Very low |
| Lf-dTMS-Fp1Fp2 vs cTBS-F3+F4 |  |  |  |  | -3.88 (-16.87,9.12) | ⨁◯◯◯ Very low |
| Lf-dTMS-Fp1Fp2 vs cTBS-Fp2 |  |  |  |  | -4.03 (-16.91,8.86) | ⨁◯◯◯ Very low |
| Lf-dTMS-Fp1Fp2 vs cTBS-Fp1 |  |  |  |  | -4.84 (-17.42,7.73) | ⨁◯◯◯ Very low |
| Lf-dTMS-Fp1Fp2 vs prLf-rTMS-F3 |  |  |  |  | -5.25 (-19.17,8.67) | ⨁◯◯◯ Very low |
| Lf-dTMS-Fp1Fp2 vs Lf-rTMS-F3 |  |  |  |  | -4.92 (-16.53,6.69) | ⨁◯◯◯ Very low |
| Lf-dTMS-Fp1Fp2 vs Hf-rTMS-F4 |  |  |  |  | -5.50 (-17.22,6.21) | ⨁◯◯◯ Very low |
| Lf-dTMS-Fp1Fp2 vs Sham | ***-10.33 (-17.80,-2.86)** | ⨁⨁⨁◯ Medium | 29.09 (8.94) | ⨁⨁◯◯ Low | -5.35 (-15.36,4.66) | ⨁⨁⨁◯ Medium |
| Lf-dTMS-Fp1Fp2 vs a-tDCS-Fp3 + c-tDCS-F8 |  |  |  |  | -6.35 (-19.88,7.19) | ⨁◯◯◯ Very low |
| Lf-dTMS-Fp1Fp2 vs neuronavigated Lf-rTMS-F5+F6 |  |  |  |  | -6.55 (-19.44,6.35) | ⨁◯◯◯ Very low |
| Lf-dTMS-Fp1Fp2 vs Hf-rTMS-F3 |  |  |  |  | -10.00 (-25.72,5.72) | ⨁⨁◯◯ Low |
| Lf-dTMS-Fp1Fp2 vs a-tDCS-Fz + c-tDCS-extracephalic |  |  |  |  | -10.49 (-25.60,4.62) | ⨁⨁◯◯ Low |
| Lf-rTMS-F5+F6 vs Lf-rTMS-Fz |  |  |  |  | -1.21 (-7.88,5.47) | ⨁◯◯◯ Very low |
| Lf-rTMS-F5+F6 vs Lf-rTMS-Fp1 |  |  |  |  | -1.29 (-10.94,8.37) | ⨁◯◯◯ Very low |
| Lf-rTMS-F5+F6 vs neuronavigated Lf-rTMS-F6 |  |  |  |  | -1.59 (-11.35,8.18) | ⨁◯◯◯ Very low |
| Lf-rTMS-F5+F6 vs c-tDCS-Fp1 + a-tDCS-O2 |  |  |  |  | -2.00 (-12.36,8.36) | ⨁◯◯◯ Very low |
| Lf-rTMS-F5+F6 vs c-tDCS-Fz + a-tDCS-extracephalic |  |  |  |  | -2.06 (-11.90,7.79) | ⨁◯◯◯ Very low |
| Lf-rTMS-F5+F6 vs Lf-rTMS-Fp2 |  |  |  |  | -2.27 (-10.53,6.00) | ⨁◯◯◯ Very low |
| Lf-rTMS-F5+F6 vs alpha-TMS-F3+F4 |  |  |  |  | -2.47 (-12.33,7.39) | ⨁◯◯◯ Very low |
| Lf-rTMS-F5+F6 vs cTBS-F3+F4 |  |  |  |  | -2.90 (-12.67,6.87) | ⨁◯◯◯ Very low |
| Lf-rTMS-F5+F6 vs cTBS-Fp2 |  |  |  |  | -3.05 (-12.67,6.57) | ⨁◯◯◯ Very low |
| Lf-rTMS-F5+F6 vs cTBS-Fp1 |  |  |  |  | -3.86 (-13.07,5.34) | ⨁◯◯◯ Very low |
| Lf-rTMS-F5+F6 vs prLf-rTMS-F3 |  |  |  |  | -4.27 (-15.23,6.68) | ⨁◯◯◯ Very low |
| Lf-rTMS-F5+F6 vs Lf-rTMS-F3 |  |  |  |  | -3.94 (-11.76,3.87) | ⨁◯◯◯ Very low |
| Lf-rTMS-F5+F6 vs Hf-rTMS-F4 |  |  |  |  | -4.52 (-12.52,3.47) | ⨁◯◯◯ Very low |
| Lf-rTMS-F5+F6 vs Sham | ***-4.78 (-7.35,-2.21)** | ⨁⨁◯◯ Low |  |  | -4.37 (-9.54,0.81) | ⨁⨁◯◯ Low |
| Lf-rTMS-F5+F6 vs a-tDCS-Fp3 + c-tDCS-F8 |  |  |  |  | -5.37 (-15.84,5.11) | ⨁◯◯◯ Very low |
| Lf-rTMS-F5+F6 vs neuronavigated Lf-rTMS-F5+F6 |  |  |  |  | -5.57 (-15.21,4.07) | ⨁◯◯◯ Very low |
| Lf-rTMS-F5+F6 vs Hf-rTMS-F3 |  |  |  |  | -9.02 (-22.20,4.15) | ⨁◯◯◯ Very low |
| Lf-rTMS-F5+F6 vs a-tDCS-Fz + c-tDCS-extracephalic |  |  |  |  | -9.51 (-21.96,2.94) | ⨁◯◯◯ Very low |
| Lf-rTMS-Fz vs Lf-rTMS-Fp1 |  |  |  |  | -0.08 (-9.26,9.09) | ⨁◯◯◯ Very low |
| Lf-rTMS-Fz vs neuronavigated Lf-rTMS-F6 |  |  |  |  | -0.38 (-9.67,8.91) | ⨁◯◯◯ Very low |
| Lf-rTMS-Fz vs c-tDCS-Fp1 + a-tDCS-O2 |  |  |  |  | -0.79 (-10.71,9.12) | ⨁◯◯◯ Very low |
| Lf-rTMS-Fz vs c-tDCS-Fz + a-tDCS-extracephalic |  |  |  |  | -0.85 (-10.23,8.52) | ⨁◯◯◯ Very low |
| Lf-rTMS-Fz vs Lf-rTMS-Fp2 |  |  |  |  | -1.06 (-8.76,6.64) | ⨁◯◯◯ Very low |
| Lf-rTMS-Fz vs alpha-TMS-F3+F4 |  |  |  |  | -1.26 (-10.65,8.13) | ⨁◯◯◯ Very low |
| Lf-rTMS-Fz vs cTBS-F3+F4 |  |  |  |  | -1.69 (-10.99,7.60) | ⨁◯◯◯ Very low |
| Lf-rTMS-Fz vs cTBS-Fp2 |  |  |  |  | -1.84 (-10.98,7.30) | ⨁◯◯◯ Very low |
| Lf-rTMS-Fz vs cTBS-Fp1 |  |  |  |  | -2.66 (-11.36,6.04) | ⨁◯◯◯ Very low |
| Lf-rTMS-Fz vs prLf-rTMS-F3 |  |  |  |  | -3.07 (-13.64,7.50) | ⨁◯◯◯ Very low |
| Lf-rTMS-Fz vs Lf-rTMS-F3 |  |  |  |  | -2.74 (-10.00,4.52) | ⨁◯◯◯ Very low |
| Lf-rTMS-Fz vs Hf-rTMS-F4 |  |  |  |  | -3.32 (-10.72,4.08) | ⨁◯◯◯ Very low |
| Lf-rTMS-Fz vs Sham | ***-2.81 (-3.91,-1.71)** | ⨁⨁◯◯ Low |  |  | -3.16 (-7.37,1.05) | ⨁⨁◯◯ Low |
| Lf-rTMS-Fz vs a-tDCS-Fp3 + c-tDCS-F8 |  |  |  |  | -4.16 (-14.20,5.87) | ⨁◯◯◯ Very low |
| Lf-rTMS-Fz vs neuronavigated Lf-rTMS-F5+F6 |  |  |  |  | -4.36 (-13.52,4.80) | ⨁◯◯◯ Very low |
| Lf-rTMS-Fz vs Hf-rTMS-F3 |  |  |  |  | -7.82 (-20.65,5.01) | ⨁◯◯◯ Very low |
| Lf-rTMS-Fz vs a-tDCS-Fz + c-tDCS-extracephalic |  |  |  |  | -8.31 (-20.38,3.77) | ⨁◯◯◯ Very low |
| Lf-rTMS-Fp1 vs neuronavigated Lf-rTMS-F6 |  |  |  |  | -0.30 (-11.92,11.32) | ⨁◯◯◯ Very low |
| Lf-rTMS-Fp1 vs c-tDCS-Fp1 + a-tDCS-O2 |  |  |  |  | -0.71 (-12.83,11.41) | ⨁◯◯◯ Very low |
| Lf-rTMS-Fp1 vs c-tDCS-Fz + a-tDCS-extracephalic |  |  |  |  | -0.77 (-12.46,10.92) | ⨁◯◯◯ Very low |
| Lf-rTMS-Fp1 vs Lf-rTMS-Fp2 |  |  |  |  | -0.98 (-11.37,9.41) | ⨁◯◯◯ Very low |
| Lf-rTMS-Fp1 vs alpha-TMS-F3+F4 |  |  |  |  | -1.18 (-12.88,10.52) | ⨁◯◯◯ Very low |
| Lf-rTMS-Fp1 vs cTBS-F3+F4 |  |  |  |  | -1.61 (-13.23,10.01) | ⨁◯◯◯ Very low |
| Lf-rTMS-Fp1 vs cTBS-Fp2 |  |  |  |  | -1.76 (-13.26,9.74) | ⨁◯◯◯ Very low |
| Lf-rTMS-Fp1 vs cTBS-Fp1 |  |  |  |  | -2.58 (-13.73,8.58) | ⨁◯◯◯ Very low |
| Lf-rTMS-Fp1 vs prLf-rTMS-F3 |  |  |  |  | -2.99 (-15.63,9.66) | ⨁◯◯◯ Very low |
| Lf-rTMS-Fp1 vs Lf-rTMS-F3 |  |  |  |  | -2.66 (-12.70,7.39) | ⨁◯◯◯ Very low |
| Lf-rTMS-Fp1 vs Hf-rTMS-F4 |  |  |  |  | -3.24 (-13.41,6.94) | ⨁◯◯◯ Very low |
| Lf-rTMS-Fp1 vs Sham | -3.08 (-6.37,0.21) | ⨁◯◯◯ Very low |  |  | -3.08 (-11.23,5.07) | ⨁⨁◯◯ Low |
| Lf-rTMS-Fp1 vs a-tDCS-Fp3 + c-tDCS-F8 |  |  |  |  | -4.08 (-16.30,8.14) | ⨁◯◯◯ Very low |
| Lf-rTMS-Fp1 vs neuronavigated Lf-rTMS-F5+F6 |  |  |  |  | -4.28 (-15.80,7.24) | ⨁◯◯◯ Very low |
| Lf-rTMS-Fp1 vs Hf-rTMS-F3 |  |  |  |  | -7.74 (-22.34,6.87) | ⨁◯◯◯ Very low |
| Lf-rTMS-Fp1 vs a-tDCS-Fz + c-tDCS-extracephalic |  |  |  |  | -8.22 (-22.17,5.73) | ⨁◯◯◯ Very low |
| neuronavigated Lf-rTMS-F6 vs c-tDCS-Fp1 + a-tDCS-O2 |  |  |  |  | -0.41 (-12.62,11.80) | ⨁◯◯◯ Very low |
| neuronavigated Lf-rTMS-F6 vs c-tDCS-Fz + a-tDCS-extracephalic |  |  |  |  | -0.47 (-12.25,11.31) | ⨁◯◯◯ Very low |
| neuronavigated Lf-rTMS-F6 vs Lf-rTMS-Fp2 |  |  |  |  | -0.68 (-11.17,9.82) | ⨁◯◯◯ Very low |
| neuronavigated Lf-rTMS-F6 vs alpha-TMS-F3+F4 |  |  |  |  | -0.88 (-12.67,10.91) | ⨁◯◯◯ Very low |
| neuronavigated Lf-rTMS-F6 vs cTBS-F3+F4 |  |  |  |  | -1.31 (-13.03,10.40) | ⨁◯◯◯ Very low |
| neuronavigated Lf-rTMS-F6 vs cTBS-Fp2 |  |  |  |  | -1.46 (-13.05,10.13) | ⨁◯◯◯ Very low |
| neuronavigated Lf-rTMS-F6 vs cTBS-Fp1 |  |  |  |  | -2.28 (-13.52,8.97) | ⨁◯◯◯ Very low |
| neuronavigated Lf-rTMS-F6 vs prLf-rTMS-F3 |  |  |  |  | -2.69 (-15.42,10.04) | ⨁◯◯◯ Very low |
| neuronavigated Lf-rTMS-F6 vs Lf-rTMS-F3 |  |  |  |  | -2.36 (-12.51,7.80) | ⨁◯◯◯ Very low |
| neuronavigated Lf-rTMS-F6 vs Hf-rTMS-F4 |  |  |  |  | -2.94 (-13.22,7.34) | ⨁◯◯◯ Very low |
| neuronavigated Lf-rTMS-F6 vs Sham | -2.78 (-6.38,0.82) | ⨁◯◯◯ Very low |  |  | -2.78 (-11.06,5.50) | ⨁⨁◯◯ Low |
| neuronavigated Lf-rTMS-F6 vs a-tDCS-Fp3 + c-tDCS-F8 |  |  |  |  | -3.78 (-16.09,8.53) | ⨁◯◯◯ Very low |
| neuronavigated Lf-rTMS-F6 vs neuronavigated Lf-rTMS-F5+F6 |  |  |  |  | -3.98 (-15.59,7.63) | ⨁◯◯◯ Very low |
| neuronavigated Lf-rTMS-F6 vs Hf-rTMS-F3 |  |  |  |  | -7.44 (-22.12,7.24) | ⨁◯◯◯ Very low |
| neuronavigated Lf-rTMS-F6 vs a-tDCS-Fz + c-tDCS-extracephalic |  |  |  |  | -7.92 (-21.95,6.10) | ⨁◯◯◯ Very low |
| c-tDCS-Fp1 + a-tDCS-O2 vs c-tDCS-Fz + a-tDCS-extracephalic |  |  |  |  | -0.06 (-12.33,12.22) | ⨁◯◯◯ Very low |
| c-tDCS-Fp1 + a-tDCS-O2 vs Lf-rTMS-Fp2 |  |  |  |  | -0.27 (-11.32,10.78) | ⨁◯◯◯ Very low |
| c-tDCS-Fp1 + a-tDCS-O2 vs alpha-TMS-F3+F4 |  |  |  |  | -0.47 (-12.76,11.82) | ⨁◯◯◯ Very low |
| c-tDCS-Fp1 + a-tDCS-O2 vs cTBS-F3+F4 |  |  |  |  | -0.90 (-13.12,11.31) | ⨁◯◯◯ Very low |
| c-tDCS-Fp1 + a-tDCS-O2 vs cTBS-Fp2 |  |  |  |  | -1.05 (-13.15,11.05) | ⨁◯◯◯ Very low |
| c-tDCS-Fp1 + a-tDCS-O2 vs cTBS-Fp1 |  |  |  |  | -1.87 (-13.63,9.90) | ⨁◯◯◯ Very low |
| c-tDCS-Fp1 + a-tDCS-O2 vs prLf-rTMS-F3 |  |  |  |  | -2.28 (-15.47,10.92) | ⨁◯◯◯ Very low |
| c-tDCS-Fp1 + a-tDCS-O2 vs Lf-rTMS-F3 |  |  |  |  | -1.95 (-12.67,8.78) | ⨁◯◯◯ Very low |
| c-tDCS-Fp1 + a-tDCS-O2 vs Hf-rTMS-F4 |  |  |  |  | -2.53 (-13.37,8.32) | ⨁◯◯◯ Very low |
| c-tDCS-Fp1 + a-tDCS-O2 vs Sham | -2.37 (-7.36,2.62) | ⨁◯◯◯ Very low |  |  | -2.37 (-11.35,6.61) | ⨁⨁◯◯ Low |
| c-tDCS-Fp1 + a-tDCS-O2 vs a-tDCS-Fp3 + c-tDCS-F8 |  |  |  |  | -3.37 (-16.16,9.42) | ⨁◯◯◯ Very low |
| c-tDCS-Fp1 + a-tDCS-O2 vs neuronavigated Lf-rTMS-F5+F6 |  |  |  |  | -3.57 (-15.68,8.54) | ⨁◯◯◯ Very low |
| c-tDCS-Fp1 + a-tDCS-O2 vs Hf-rTMS-F3 |  |  |  |  | -7.03 (-22.11,8.05) | ⨁◯◯◯ Very low |
| c-tDCS-Fp1 + a-tDCS-O2 vs a-tDCS-Fz + c-tDCS-extracephalic |  |  |  |  | -7.51 (-21.96,6.93) | ⨁◯◯◯ Very low |
| c-tDCS-Fz + a-tDCS-extracephalic vs Lf-rTMS-Fp2 |  |  |  |  | -0.21 (-10.77,10.36) | ⨁◯◯◯ Very low |
| c-tDCS-Fz + a-tDCS-extracephalic vs alpha-TMS-F3+F4 |  |  |  |  | -0.41 (-12.27,11.45) | ⨁◯◯◯ Very low |
| c-tDCS-Fz + a-tDCS-extracephalic vs cTBS-F3+F4 |  |  |  |  | -0.84 (-12.62,10.94) | ⨁◯◯◯ Very low |
| c-tDCS-Fz + a-tDCS-extracephalic vs cTBS-Fp2 |  |  |  |  | -0.99 (-12.65,10.67) | ⨁◯◯◯ Very low |
| c-tDCS-Fz + a-tDCS-extracephalic vs cTBS-Fp1 |  |  |  |  | -1.81 (-13.12,9.51) | ⨁◯◯◯ Very low |
| c-tDCS-Fz + a-tDCS-extracephalic vs prLf-rTMS-F3 |  |  |  |  | -2.22 (-15.01,10.57) | ⨁◯◯◯ Very low |
| c-tDCS-Fz + a-tDCS-extracephalic vs Lf-rTMS-F3 |  |  |  |  | -1.89 (-12.11,8.34) | ⨁◯◯◯ Very low |
| c-tDCS-Fz + a-tDCS-extracephalic vs Hf-rTMS-F4 |  |  |  |  | -2.47 (-12.82,7.89) | ⨁◯◯◯ Very low |
| c-tDCS-Fz + a-tDCS-extracephalic vs Sham | -2.31 (-6.12,1.50) | ⨁◯◯◯ Very low | -3.73 (404.23) | ⨁◯◯◯ Very low | -2.31 (-10.68,6.06) | ⨁⨁⨁◯ Medium |
| c-tDCS-Fz + a-tDCS-extracephalic vs a-tDCS-Fp3 + c-tDCS-F8 |  |  |  |  | -3.31 (-15.68,9.06) | ⨁◯◯◯ Very low |
| c-tDCS-Fz + a-tDCS-extracephalic vs neuronavigated Lf-rTMS-F5+F6 |  |  |  |  | -3.51 (-15.19,8.17) | ⨁◯◯◯ Very low |
| c-tDCS-Fz + a-tDCS-extracephalic vs Hf-rTMS-F3 |  |  |  |  | -6.97 (-21.70,7.76) | ⨁◯◯◯ Very low |
| c-tDCS-Fz + a-tDCS-extracephalic vs a-tDCS-Fz + c-tDCS-extracephalic | ***-7.45 (-9.01,-5.90)** | ⨁⨁◯◯ Low | 4.62 (808.81) | ⨁◯◯◯ Very low | -7.45 (-15.07,0.17) | ⨁⨁⨁◯ Medium |
| Lf-rTMS-Fp2 vs alpha-TMS-F3+F4 |  |  |  |  | -0.20 (-10.78,10.38) | ⨁◯◯◯ Very low |
| Lf-rTMS-Fp2 vs cTBS-F3+F4 |  |  |  |  | -0.63 (-11.13,9.86) | ⨁◯◯◯ Very low |
| Lf-rTMS-Fp2 vs cTBS-Fp2 |  |  |  |  | -0.78 (-11.14,9.58) | ⨁◯◯◯ Very low |
| Lf-rTMS-Fp2 vs cTBS-Fp1 |  |  |  |  | -1.60 (-11.57,8.38) | ⨁◯◯◯ Very low |
| Lf-rTMS-Fp2 vs prLf-rTMS-F3 |  |  |  |  | -2.01 (-13.62,9.60) | ⨁◯◯◯ Very low |
| Lf-rTMS-Fp2 vs Lf-rTMS-F3 |  |  |  |  | -1.68 (-10.39,7.03) | ⨁◯◯◯ Very low |
| Lf-rTMS-Fp2 vs Hf-rTMS-F4 |  |  |  |  | -2.26 (-10.95,6.43) | ⨁◯◯◯ Very low |
| Lf-rTMS-Fp2 vs Sham | -2.13 (-6.32,2.06) | ⨁◯◯◯ Very low | -3.17 (9.85) | ⨁⨁◯◯ Low | -2.10 (-8.55,4.34) | ⨁⨁⨁◯ Medium |
| Lf-rTMS-Fp2 vs a-tDCS-Fp3 + c-tDCS-F8 |  |  |  |  | -3.10 (-14.26,8.05) | ⨁◯◯◯ Very low |
| Lf-rTMS-Fp2 vs neuronavigated Lf-rTMS-F5+F6 |  |  |  |  | -3.30 (-13.68,7.08) | ⨁◯◯◯ Very low |
| Lf-rTMS-Fp2 vs Hf-rTMS-F3 |  |  |  |  | -6.76 (-20.37,6.85) | ⨁◯◯◯ Very low |
| Lf-rTMS-Fp2 vs a-tDCS-Fz + c-tDCS-extracephalic |  |  |  |  | -7.25 (-20.27,5.78) | ⨁◯◯◯ Very low |
| alpha-TMS-F3+F4 vs cTBS-F3+F4 |  |  |  |  | -0.43 (-12.23,11.36) | ⨁◯◯◯ Very low |
| alpha-TMS-F3+F4 vs cTBS-Fp2 |  |  |  |  | -0.58 (-12.25,11.09) | ⨁◯◯◯ Very low |
| alpha-TMS-F3+F4 vs cTBS-Fp1 |  |  |  |  | -1.39 (-12.73,9.94) | ⨁◯◯◯ Very low |
| alpha-TMS-F3+F4 vs prLf-rTMS-F3 |  |  |  |  | -1.81 (-14.61,11.00) | ⨁◯◯◯ Very low |
| alpha-TMS-F3+F4 vs Lf-rTMS-F3 |  |  |  |  | -1.48 (-11.72,8.77) | ⨁◯◯◯ Very low |
| alpha-TMS-F3+F4 vs Hf-rTMS-F4 |  |  |  |  | -2.06 (-12.43,8.31) | ⨁◯◯◯ Very low |
| alpha-TMS-F3+F4 vs Sham | -1.90 (-5.75,1.95) | ⨁◯◯◯ Very low |  |  | -1.90 (-10.29,6.49) | ⨁⨁◯◯ Low |
| alpha-TMS-F3+F4 vs a-tDCS-Fp3 + c-tDCS-F8 |  |  |  |  | -2.90 (-15.28,9.48) | ⨁◯◯◯ Very low |
| alpha-TMS-F3+F4 vs neuronavigated Lf-rTMS-F5+F6 |  |  |  |  | -3.10 (-14.79,8.59) | ⨁◯◯◯ Very low |
| alpha-TMS-F3+F4 vs Hf-rTMS-F3 |  |  |  |  | -6.56 (-21.30,8.19) | ⨁◯◯◯ Very low |
| alpha-TMS-F3+F4 vs a-tDCS-Fz + c-tDCS-extracephalic |  |  |  |  | -7.04 (-21.14,7.05) | ⨁◯◯◯ Very low |
| cTBS-F3+F4 vs cTBS-Fp2 |  |  |  |  | -0.15 (-11.74,11.45) | ⨁◯◯◯ Very low |
| cTBS-F3+F4 vs cTBS-Fp1 |  |  |  |  | -0.96 (-12.21,10.29) | ⨁◯◯◯ Very low |
| cTBS-F3+F4 vs prLf-rTMS-F3 |  |  |  |  | -1.37 (-14.11,11.36) | ⨁◯◯◯ Very low |
| cTBS-F3+F4 vs Lf-rTMS-F3 |  |  |  |  | -1.04 (-11.20,9.11) | ⨁◯◯◯ Very low |
| cTBS-F3+F4 vs Hf-rTMS-F4 |  |  |  |  | -1.62 (-11.91,8.66) | ⨁◯◯◯ Very low |
| cTBS-F3+F4 vs Sham | -1.47 (-5.08,2.14) | ⨁◯◯◯ Very low |  |  | -1.47 (-9.75,6.82) | ⨁⨁◯◯ Low |
| cTBS-F3+F4 vs a-tDCS-Fp3 + c-tDCS-F8 |  |  |  |  | -2.47 (-14.78,9.84) | ⨁◯◯◯ Very low |
| cTBS-F3+F4 vs neuronavigated Lf-rTMS-F5+F6 |  |  |  |  | -2.67 (-14.28,8.94) | ⨁◯◯◯ Very low |
| cTBS-F3+F4 vs Hf-rTMS-F3 |  |  |  |  | -6.12 (-20.81,8.56) | ⨁◯◯◯ Very low |
| cTBS-F3+F4 vs a-tDCS-Fz + c-tDCS-extracephalic |  |  |  |  | -6.61 (-20.64,7.42) | ⨁◯◯◯ Very low |
| cTBS-Fp2 vs cTBS-Fp1 |  |  |  |  | -0.81 (-11.94,10.31) | ⨁◯◯◯ Very low |
| cTBS-Fp2 vs prLf-rTMS-F3 |  |  |  |  | -1.23 (-13.84,11.39) | ⨁◯◯◯ Very low |
| cTBS-Fp2 vs Lf-rTMS-F3 |  |  |  |  | -0.90 (-10.91,9.12) | ⨁◯◯◯ Very low |
| cTBS-Fp2 vs Hf-rTMS-F4 |  |  |  |  | -1.48 (-11.62,8.67) | ⨁◯◯◯ Very low |
| cTBS-Fp2 vs Sham | -1.32 (-4.51,1.87) | ⨁◯◯◯ Very low |  |  | -1.32 (-9.43,6.79) | ⨁⨁◯◯ Low |
| cTBS-Fp2 vs a-tDCS-Fp3 + c-tDCS-F8 |  |  |  |  | -2.32 (-14.51,9.87) | ⨁◯◯◯ Very low |
| cTBS-Fp2 vs neuronavigated Lf-rTMS-F5+F6 |  |  |  |  | -2.52 (-14.01,8.97) | ⨁◯◯◯ Very low |
| cTBS-Fp2 vs Hf-rTMS-F3 |  |  |  |  | -5.98 (-20.56,8.61) | ⨁◯◯◯ Very low |
| cTBS-Fp2 vs a-tDCS-Fz + c-tDCS-extracephalic |  |  |  |  | -6.46 (-20.39,7.46) | ⨁◯◯◯ Very low |
| cTBS-Fp1 vs prLf-rTMS-F3 |  |  |  |  | -0.41 (-12.72,11.89) | ⨁◯◯◯ Very low |
| cTBS-Fp1 vs Lf-rTMS-F3 |  |  |  |  | -0.08 (-9.69,9.53) | ⨁◯◯◯ Very low |
| cTBS-Fp1 vs Hf-rTMS-F4 |  |  |  |  | -0.66 (-10.41,9.09) | ⨁◯◯◯ Very low |
| cTBS-Fp1 vs Sham | -0.51 (-2.03,1.02) | ⨁◯◯◯ Very low |  |  | -0.51 (-8.12,7.11) | ⨁⨁◯◯ Low |
| cTBS-Fp1 vs a-tDCS-Fp3 + c-tDCS-F8 |  |  |  |  | -1.51 (-13.37,10.36) | ⨁◯◯◯ Very low |
| cTBS-Fp1 vs neuronavigated Lf-rTMS-F5+F6 |  |  |  |  | -1.71 (-12.85,9.44) | ⨁◯◯◯ Very low |
| cTBS-Fp1 vs Hf-rTMS-F3 |  |  |  |  | -5.16 (-19.47,9.15) | ⨁◯◯◯ Very low |
| cTBS-Fp1 vs a-tDCS-Fz + c-tDCS-extracephalic |  |  |  |  | -5.65 (-19.29,7.99) | ⨁◯◯◯ Very low |
| prLf-rTMS-F3 vs Lf-rTMS-F3 | 0.33 (-1.50,2.16) | ⨁◯◯◯ Very low | -0.85 (1608.52) | ⨁◯◯◯ Very low | 0.33 (-7.35,8.01) | ⨁⨁⨁◯ Medium |
| prLf-rTMS-F3 vs Hf-rTMS-F4 |  |  |  |  | -0.25 (-11.68,11.18) | ⨁◯◯◯ Very low |
| prLf-rTMS-F3 vs Sham |  |  |  |  | -0.09 (-9.76,9.57) | ⨁◯◯◯ Very low |
| prLf-rTMS-F3 vs a-tDCS-Fp3 + c-tDCS-F8 |  |  |  |  | -1.09 (-14.38,12.19) | ⨁◯◯◯ Very low |
| prLf-rTMS-F3 vs neuronavigated Lf-rTMS-F5+F6 |  |  |  |  | -1.29 (-13.93,11.34) | ⨁◯◯◯ Very low |
| prLf-rTMS-F3 vs Hf-rTMS-F3 |  |  |  |  | -4.75 (-20.26,10.76) | ⨁◯◯◯ Very low |
| prLf-rTMS-F3 vs a-tDCS-Fz + c-tDCS-extracephalic |  |  |  |  | -5.24 (-20.12,9.65) | ⨁◯◯◯ Very low |
| Lf-rTMS-F3 vs Hf-rTMS-F4 |  |  |  |  | -0.58 (-9.05,7.89) | ⨁◯◯◯ Very low |
| Lf-rTMS-F3 vs Sham | -0.05 (-8.03,7.92) | ⨁◯◯◯ Very low | -0.17 (805.64) | ⨁◯◯◯ Very low | -0.42 (-6.30,5.45) | ⨁⨁⨁◯ Medium |
| Lf-rTMS-F3 vs a-tDCS-Fp3 + c-tDCS-F8 |  |  |  |  | -1.42 (-12.26,9.41) | ⨁◯◯◯ Very low |
| Lf-rTMS-F3 vs neuronavigated Lf-rTMS-F5+F6 |  |  |  |  | -1.62 (-11.66,8.41) | ⨁◯◯◯ Very low |
| Lf-rTMS-F3 vs Hf-rTMS-F3 |  |  |  |  | -5.08 (-18.55,8.39) | ⨁◯◯◯ Very low |
| Lf-rTMS-F3 vs a-tDCS-Fz + c-tDCS-extracephalic |  |  |  |  | -5.57 (-18.32,7.19) | ⨁◯◯◯ Very low |
| Hf-rTMS-F4 vs Sham | -0.68 (-4.61,3.25) | ⨁◯◯◯ Very low | 6.50 (9.66) | ⨁⨁◯◯ Low | 0.16 (-5.94,6.25) | ⨁⨁⨁◯ Medium |
| Hf-rTMS-F4 vs a-tDCS-Fp3 + c-tDCS-F8 |  |  |  |  | -0.84 (-11.80,10.11) | ⨁◯◯◯ Very low |
| Hf-rTMS-F4 vs neuronavigated Lf-rTMS-F5+F6 |  |  |  |  | -1.04 (-11.21,9.12) | ⨁◯◯◯ Very low |
| Hf-rTMS-F4 vs Hf-rTMS-F3 | -4.50 (-11.86,2.86) | ⨁◯◯◯ Very low | -0.31 (3855.40) | ⨁◯◯◯ Very low | -4.50 (-14.98,5.98) | ⨁⨁⨁◯ Medium |
| Hf-rTMS-F4 vs a-tDCS-Fz + c-tDCS-extracephalic |  |  |  |  | -4.99 (-17.84,7.87) | ⨁◯◯◯ Very low |
| Sham vs a-tDCS-Fp3 + c-tDCS-F8 | -1.00 (-6.23,4.23) | ⨁◯◯◯ Very low |  |  | -1.00 (-10.11,8.11) | ⨁⨁◯◯ Low |
| Sham vs neuronavigated Lf-rTMS-F5+F6 | -1.20 (-4.45,2.05) | ⨁◯◯◯ Very low |  |  | -1.20 (-9.34,6.94) | ⨁⨁◯◯ Low |
| Sham vs Hf-rTMS-F3 |  |  |  |  | -4.66 (-16.78,7.46) | ⨁◯◯◯ Very low |
| Sham vs a-tDCS-Fz + c-tDCS-extracephalic |  |  |  |  | -5.14 (-16.46,6.18) | ⨁◯◯◯ Very low |
| a-tDCS-Fp3 + c-tDCS-F8 vs neuronavigated Lf-rTMS-F5+F6 |  |  |  |  | -0.20 (-12.41,12.01) | ⨁◯◯◯ Very low |
| a-tDCS-Fp3 + c-tDCS-F8 vs Hf-rTMS-F3 |  |  |  |  | -3.66 (-18.82,11.50) | ⨁◯◯◯ Very low |
| a-tDCS-Fp3 + c-tDCS-F8 vs a-tDCS-Fz + c-tDCS-extracephalic |  |  |  |  | -4.14 (-18.67,10.39) | ⨁◯◯◯ Very low |
| neuronavigated Lf-rTMS-F5+F6 vs Hf-rTMS-F3 |  |  |  |  | -3.46 (-18.05,11.14) | ⨁◯◯◯ Very low |
| neuronavigated Lf-rTMS-F5+F6 vs a-tDCS-Fz + c-tDCS-extracephalic |  |  |  |  | -3.94 (-17.88,10.00) | ⨁◯◯◯ Very low |
| Hf-rTMS-F3 vs a-tDCS-Fz + c-tDCS-extracephalic |  |  |  |  | -0.49 (-17.07,16.10) | ⨁◯◯◯ Very low |

Abbreviation: 95%CIs: 95% confidence intervals; ACC: anterior cingulate cortex; alpha-TMS-F3+F4: alpha EEG guided-TMS over F3+F4; a-tDCS-Fp3 + c-tDCS-F8: anode tDCS over Fp3 plus cathode tDCS over F8; a-tDCS-Fz + c-tDCS-extracephalic: anode tDCS over Fz plus cathode tDCS over extracephalic region; CBT: cognitive behavioral therapy; CGI-S: clinical global impression scale-severity; cTBS: continuous theta burst stimulation; cTBS-F3+F4: cTBS over F3+F4; cTBS-Fp1: cTBS over Fp1; cTBS-Fp2: cTBS over Fp2; c-tDCS-Fp1 + a-tDCS-O2: cathode tDCS over Fp1 plus anode tDCS over O2; c-tDCS-Fp2 + a-tDCS-F5: cathode tDCS over Fp2 plus anode tDCS over F5; c-tDCS-Fz + a-tDCS-extracephalic: cathode tDCS over Fz plus anode tDCS over extracephalic region; DLPFC: dorsolateral prefrontal cortex; dmPFC: dorsal medial prefrontal cortex; DSM: diagnostic and statistical manual of mental disorders; dTMS: deep TMS; EEG: electroencephalography; Hf-dTMS-Fp1Fp2: high frequency dTMS over Fp1Fp2; Hf-rTMS-F3: high frequency rTMS over F3; Hf-rTMS-F3+F4: high frequency rTMS over F3+F4; Hf-rTMS-F4: high frequency rTMS over F4; ICD: international classification of diseases; Lf-dTMS-Fp1Fp2: low frequency dTMS over Fp1Fp2; Lf-rTMS-F3: low frequency rTMS over F3; Lf-rTMS-F4: low frequency rTMS over F4; Lf-rTMS-F5+F6: low frequency rTMS over F5+F6; Lf-rTMS-Fp1: low frequency rTMS over Fp1; Lf-rTMS-Fp2: low frequency rTMS over Fp2; Lf-rTMS-Fz: low frequency rTMS over Fz; MD: mean difference; neuronavigated Lf-rTMS-F5+F6: neuronavigated low frequency rTMS over F5+F6; neuronavigated Lf-rTMS-F6: neuronavigated low frequency rTMS over F6; NIBS: noninvasive brain stimulation; NMA: network meta-analysis; OCD: obsessive-compulsive disorder; OFC: orbitofrontal cortex; OR: odds ratio; prLf-rTMS-F3: priming and low frequency rTMS over F3; RCT: randomized controlled trial; rTMS: repetitive TMS; Sham: sham control; SMD: standardized mean difference; SUCRA: surface under the cumulative ranking curve; tDCS: transcranial direct current stimulation; TMS: transcranial magnetic stimulation; YBOCS: Yale-Brown obsessive compulsive scale

**References of all supplement tables:**

1. PAGE MJ, MCKENZIE JE, BOSSUYT PM et al. The PRISMA 2020 statement: an updated guideline for reporting systematic reviews. *Bmj.* 2021;372(n71.

2. BANDELOW B, ALLGULANDER C, BALDWIN DS et al. World Federation of Societies of Biological Psychiatry (WFSBP) guidelines for treatment of anxiety, obsessive-compulsive and posttraumatic stress disorders - Version 3. Part II: OCD and PTSD. *World J Biol Psychiatry.* 2022:1-17.

3. SHAYGANFARD M, JAHANGARD L, NAZARIBADIE M et al. Repetitive Transcranial Magnetic Stimulation Improved Symptoms of Obsessive-Compulsive Disorders but Not Executive Functions: Results from a Randomized Clinical Trial with Crossover Design and Sham Condition. *Neuropsychobiology.* 2016;74(2):115-124.

4. JAHANGARD L, HAGHIGHI M, SHYAYGANFARD M et al. Repetitive Transcranial Magnetic Stimulation Improved Symptoms of Obsessive-Compulsive Disorder, but Also Cognitive Performance: Results from a Randomized Clinical Trial with a Cross-Over Design and Sham Condition. *Neuropsychobiology.* 2016;73(4):224-232.

5. HARIKA-GERMANEAU G, RACHID F, CHATARD A et al. Continuous theta burst stimulation over the supplementary motor area in refractory obsessive-compulsive disorder treatment: A randomized sham-controlled trial. *Brain stimulation.* 2019;12(6):1565-1571.

6. MUKHERJEE A, KUMRE PK, GOYAL N, KHANRA S. Adjunctive neuronavigated accelerated continuous theta-burst stimulation in obsessive-compulsive disorder: a randomized sham-controlled study. *CNS spectrums.* 2022:1-10.

7. NARO A, BILLERI L, CANNAVO A et al. Theta burst stimulation for the treatment of obsessive-compulsive disorder: a pilot study. *Journal of neural transmission.* 2019;126(12):1667-1677.

8. THATIKONDA NS, VINOD P, BALACHANDER S, BHASKARPILLAI B, ARUMUGHAM SS, REDDY YCJ. Efficacy of Repetitive Transcranial Magnetic Stimulation on Comorbid Anxiety and Depression Symptoms in Obsessive-Compulsive Disorder: A Meta-Analysis of Randomized Sham-Controlled Trials. *Canadian journal of psychiatry Revue canadienne de psychiatrie.* 2022:7067437221121112.

9. PELLEGRINI L, GARG K, ENARA A et al. Repetitive transcranial magnetic stimulation (r-TMS) and selective serotonin reuptake inhibitor-resistance in obsessive-compulsive disorder: A meta-analysis and clinical implications. *Compr Psychiatry.* 2022;118(152339.

10. ZHOU S, FANG Y. Efficacy of Non-Invasive Brain Stimulation for Refractory Obsessive-Compulsive Disorder: A Meta-Analysis of Randomized Controlled Trials. *Brain Sci.* 2022;12(7).

11. PERERA MPN, MALLAWAARACHCHI S, MILJEVIC A, BAILEY NW, HERRING SE, FITZGERALD PB. Repetitive Transcranial Magnetic Stimulation for Obsessive-Compulsive Disorder: A Meta-analysis of Randomized, Sham-Controlled Trials. *Biol Psychiatry Cogn Neurosci Neuroimaging.* 2021;6(10):947-960.

12. MA ZR, SHI LJ. Repetitive transcranial magnetic stimulation (rTMS) augmentation of selective serotonin reuptake inhibitors (SSRIs) for SSRI-resistant obsessive-compulsive disorder (OCD): a meta-analysis of randomized controlled trials. *Int J Clin Exp Med.* 2014;7(12):4897-4905.

13. MARTIN JL, BARBANOJ MJ, PEREZ V, SACRISTAN M. Transcranial magnetic stimulation for the treatment of obsessive-compulsive disorder. *The Cochrane database of systematic reviews.* 2003;2003(3):CD003387.

14. PINTO BS, CAVENDISH BA, DA SILVA PHR et al. The Effects of Transcranial Direct Current Stimulation in Obsessive-Compulsive Disorder Symptoms: A Meta-Analysis and Integrated Electric Fields Modeling Analysis. *Biomedicines.* 2022;11(1).

15. SUHAS S, MALO PK, KUMAR V et al. Treatment strategies for serotonin reuptake inhibitor-resistant obsessive-compulsive disorder: A network meta-analysis of randomised controlled trials. *World J Biol Psychiatry.* 2022:1-16.

16. FITZSIMMONS S, VAN DER WERF YD, VAN CAMPEN AD et al. Repetitive transcranial magnetic stimulation for obsessive-compulsive disorder: A systematic review and pairwise/network meta-analysis. *Journal of affective disorders.* 2022;302(302-312.

17. LIANG K, LI H, BU X et al. Efficacy and tolerability of repetitive transcranial magnetic stimulation for the treatment of obsessive-compulsive disorder in adults: a systematic review and network meta-analysis. *Transl Psychiatry.* 2021;11(1):332.

18. AKBARI S, HASSANI-ABHARIAN P, TAJERI B. The effect of transcranial direct current stimulation (tDCS) on cerebellum in reduction of the symptoms of obsessive-compulsive disorder. *Neurocase.* 2022;28(2):135-139.

19. HARIKA-GERMANEAU G, HEIT D, CHATARD A, THIRIOUX B, LANGBOUR N, JAAFARI N. Treating refractory obsessive-compulsive disorder with transcranial direct current stimulation: An open label study. *Brain Behav.* 2020;10(7):e01648.

20. BATION R, POULET E, HAESEBAERT F, SAOUD M, BRUNELIN J. Transcranial direct current stimulation in treatment-resistant obsessive-compulsive disorder: An open-label pilot study. *Progress in neuro-psychopharmacology & biological psychiatry.* 2016;65(153-157.

21. MODIRROUSTA M, SHAMS E, KATZ C et al. The efficacy of deep repetitive transcranial magnetic stimulation over the medial prefrontal cortex in obsessive compulsive disorder: results from an open-label study. *Depression and anxiety.* 2015;32(6):445-450.

22. GREENBERG BD, GEORGE MS, MARTIN JD et al. Effect of prefrontal repetitive transcranial magnetic stimulation in obsessive-compulsive disorder: a preliminary study. *The American journal of psychiatry.* 1997;154(6):867-869.

23. TODDER D, GERSHI A, PERRY Z, KAPLAN Z, LEVINE J, AVIRAME K. Immediate Effects of Transcranial Direct Current Stimulation on Obsession-Induced Anxiety in Refractory Obsessive-Compulsive Disorder: A Pilot Study. *The journal of ECT.* 2018;34(4):e51-e57.

24. SARKHEL S, SINHA VK, PRAHARAJ SK. Adjunctive high-frequency right prefrontal repetitive transcranial magnetic stimulation (rTMS) was not effective in obsessive-compulsive disorder but improved secondary depression. *Journal of anxiety disorders.* 2010;24(5):535-539.

25. BADAWY AA, SAWY HE, HAY MAE. Efficacy of repetitive transcranial magnetic stimulation in the management of obsessive compulsive disorder. *Egypt J Neurol Psychiat Neurosurg.* 2010;47(1):393-398.

26. HOSSAIN R, SINYOR M, NESTOR S et al. Mapping the future of interventional psychiatry for the obsessive-compulsive related disorders: A scoping review. *Psychiatry research.* 2023;319(115007.

27. ADU MK, EBOREIME E, SAPARA AO, GREENSHAW AJ, CHUE P, AGYAPONG VIO. The use of repetitive transcranial magnetic stimulation for treatment of obsessive-compulsive disorder: a scoping review. *Ment Illn.* 2021;13(1):1-13.

28. BRUNELIN J, MONDINO M, BATION R, PALM U, SAOUD M, POULET E. Transcranial Direct Current Stimulation for Obsessive-Compulsive Disorder: A Systematic Review. *Brain Sci.* 2018;8(2).

29. SABA G, MOUKHEIBER A, PELISSOLO A. Transcranial cortical stimulation in the treatment of obsessive-compulsive disorders: efficacy studies. *Curr Psychiatry Rep.* 2015;17(5):36.

30. RAPINESI C, KOTZALIDIS GD, FERRACUTI S, SANI G, GIRARDI P, DEL CASALE A. Brain Stimulation in Obsessive-Compulsive Disorder (OCD): A Systematic Review. *Curr Neuropharmacol.* 2019;17(8):787-807.

31. GREEN PE, LOFTUS A, ANDERSON RA. Protocol for Transcranial Direct Current Stimulation for Obsessive-Compulsive Disorder. *Brain Sci.* 2020;10(12).

32. BALZUS L, KLAWOHN J, ELSNER B, SCHMIDT S, BRANDT SA, KATHMANN N. Non-invasive brain stimulation modulates neural correlates of performance monitoring in patients with obsessive-compulsive disorder. *Neuroimage Clin.* 2022;35(103113.

33. ADAMS TG, CISLER JM, KELMENDI B et al. Transcranial direct current stimulation targeting the medial prefrontal cortex modulates functional connectivity and enhances safety learning in obsessive-compulsive disorder: Results from two pilot studies. *Depression and anxiety.* 2022;39(1):37-48.

34. PRICE RB, GILLAN CM, HANLON C et al. Effect of Experimental Manipulation of the Orbitofrontal Cortex on Short-Term Markers of Compulsive Behavior: A Theta Burst Stimulation Study. *The American journal of psychiatry.* 2021;178(5):459-468.

35. PEDAPATI E, DIFRANCESCO M, WU S et al. Neural correlates associated with symptom provocation in pediatric obsessive compulsive disorder after a single session of sham-controlled repetitive transcranial magnetic stimulation. *Psychiatry research.* 2015;233(3):466-473.

36. GUO Q, WANG K, HAN H et al. Continuous theta burst stimulation over the bilateral supplementary motor area in obsessive-compulsive disorder treatment: A clinical randomized single-blind sham-controlled trial. *European psychiatry : the journal of the Association of European Psychiatrists.* 2022;65(1):e64.

37. JAHANBAKHSH G, ALIREZA HAJI SEYED JAVADI S, MAJIDI M, KHADEMI M, KARIMI R. Effectiveness of adjunctive low-frequency repetitive transcranial magnetic stimulation therapy over the left dorsolateral prefrontal cortex in patients with obsessive-compulsive disorder refractory to medical treatment:A double-blind, randomized clinical trial. *Asian J Psychiatr.* 2022;80(103384.

38. JOSHI M, KAR SK, DALAL PK. Safety and efficacy of early augmentation with repetitive transcranial magnetic stimulation in the treatment of drug-free patients with obsessive-compulsive disorder. *CNS spectrums.* 2022:1-7.

39. KHEDR EM, ELBEH K, SABER M, ABDELRADY Z, ABDELWARITH A. A double blind randomized clinical trial of the effectiveness of low frequency rTMS over right DLPFC or OFC for treatment of obsessive-compulsive disorder. *Journal of psychiatric research.* 2022;156(122-131.

40. VIDYA KL, RAO PG, GOYAL N. Adjuvant Priming Repetitive Transcranial Magnetic Stimulation for Treatment-Resistant Obsessive-Compulsive Disorder: In Search of a New Paradigm! *The journal of ECT.* 2022;38(1):e1-e8.

41. DUTTA P, DHYANI M, GARG S et al. Efficacy of intensive orbitofrontal continuous Theta Burst Stimulation (iOFcTBS) in Obsessive Compulsive Disorder: A Randomized Placebo Controlled Study. *Psychiatry research.* 2021;298(113784.

42. JI GJ, XIE W, YANG T et al. Pre-supplementary motor network connectivity and clinical outcome of magnetic stimulation in obsessive-compulsive disorder. *Human brain mapping.* 2021;42(12):3833-3844.

43. LIU W, SHAO H, LIAO J, YANG D, MA M, YANG J. Continuous Theta-Burst Stimulation Over the Right Orbitofrontal Cortex in Treatment-Resistant Obsessive-Compulsive Disorder Treatment: A Randomized Sham-Controlled Trial. *Int J Gen Med.* 2021;14(3109-3118.

44. MEEK BP, FOTROS A, ABO AOUN M, MODIRROUSTA M. Improvements in error-monitoring and symptoms following low-frequency rTMS of dorsal anterior cingulate cortex in obsessive compulsive disorder; a randomized, sham-controlled study. *Brain Cogn.* 2021;154(105809.

45. SILVA R, BRUNONI AR, GOERIGK S et al. Efficacy and safety of transcranial direct current stimulation as an add-on treatment for obsessive-compulsive disorder: a randomized, sham-controlled trial. *Neuropsychopharmacology : official publication of the American College of Neuropsychopharmacology.* 2021;46(5):1028-1034.

46. YOOSEFEE S, AMANAT M, SALEHI M et al. The safety and efficacy of transcranial direct current stimulation as add-on therapy to fluoxetine in obsessive-compulsive disorder: a randomized, double-blind, sham-controlled, clinical trial. *BMC psychiatry.* 2020;20(1):570.

47. BATION R, MONDINO M, LE CAMUS F, SAOUD M, BRUNELIN J. Transcranial direct current stimulation in patients with obsessive compulsive disorder: A randomized controlled trial. *European psychiatry : the journal of the Association of European Psychiatrists.* 2019;62(38-44.

48. CARMI L, TENDLER A, BYSTRITSKY A et al. Efficacy and Safety of Deep Transcranial Magnetic Stimulation for Obsessive-Compulsive Disorder: A Prospective Multicenter Randomized Double-Blind Placebo-Controlled Trial. *The American journal of psychiatry.* 2019;176(11):931-938.

49. GOWDA SM, NARAYANASWAMY JC, HAZARI N et al. Efficacy of pre-supplementary motor area transcranial direct current stimulation for treatment resistant obsessive compulsive disorder: A randomized, double blinded, sham controlled trial. *Brain stimulation.* 2019;12(4):922-929.

50. ZHANG K, FAN X, YUAN J et al. Impact of serotonin transporter gene on rTMS augmentation of SSRIs for obsessive compulsive disorder. *Neuropsychiatric disease and treatment.* 2019;15(1771-1779.

51. ARUMUGHAM SS, VS S, HN M et al. Augmentation Effect of Low-Frequency Repetitive Transcranial Magnetic Stimulation Over Presupplementary Motor Area in Obsessive-Compulsive Disorder: A Randomized Controlled Trial. *The journal of ECT.* 2018;34(4):253-257.

52. CARMI L, ALYAGON U, BARNEA-YGAEL N, ZOHAR J, DAR R, ZANGEN A. Clinical and electrophysiological outcomes of deep TMS over the medial prefrontal and anterior cingulate cortices in OCD patients. *Brain stimulation.* 2018;11(1):158-165.

53. D'URSO G, BRUNONI AR, MAZZAFERRO MP, ANASTASIA A, DE BARTOLOMEIS A, MANTOVANI A. Transcranial direct current stimulation for obsessive-compulsive disorder: A randomized, controlled, partial crossover trial. *Depression and anxiety.* 2016;33(12):1132-1140.

54. ELBEH KAM, ELSEROGY YMB, KHALIFA HE, AHMED MA, HAFEZ MH, KHEDR EM. Repetitive transcranial magnetic stimulation in the treatment of obsessive-compulsive disorders: Double blind randomized clinical trial. *Psychiatry research.* 2016;238(264-269.

55. HAWKEN ER, DILKOV D, KALUDIEV E, SIMEK S, ZHANG F, MILEV R. Transcranial Magnetic Stimulation of the Supplementary Motor Area in the Treatment of Obsessive-Compulsive Disorder: A Multi-Site Study. *Int J Mol Sci.* 2016;17(3):420.

56. PELISSOLO A, HARIKA-GERMANEAU G, RACHID F et al. Repetitive Transcranial Magnetic Stimulation to Supplementary Motor Area in Refractory Obsessive-Compulsive Disorder Treatment: a Sham-Controlled Trial. *Int J Neuropsychopharmacol.* 2016;19(8).

57. SEO HJ, JUNG YE, LIM HK, UM YH, LEE CU, CHAE JH. Adjunctive Low-frequency Repetitive Transcranial Magnetic Stimulation over the Right Dorsolateral Prefrontal Cortex in Patients with Treatment-resistant Obsessive-compulsive Disorder: A Randomized Controlled Trial. *Clin Psychopharmacol Neurosci.* 2016;14(2):153-160.

58. HAGHIGHI M, SHAYGANFARD M, JAHANGARD L et al. Repetitive Transcranial Magnetic Stimulation (rTMS) improves symptoms and reduces clinical illness in patients suffering from OCD--Results from a single-blind, randomized clinical trial with sham cross-over condition. *Journal of psychiatric research.* 2015;68(238-244.

59. MA X, HUANG Y, LIAO L, JIN Y. A randomized double-blinded sham-controlled trial of alpha electroencephalogram-guided transcranial magnetic stimulation for obsessive-compulsive disorder. *Chin Med J (Engl).* 2014;127(4):601-606.

60. NAUCZYCIEL C, LE JEUNE F, NAUDET F et al. Repetitive transcranial magnetic stimulation over the orbitofrontal cortex for obsessive-compulsive disorder: a double-blind, crossover study. *Transl Psychiatry.* 2014;4(9):e436.

61. GOMES PV, BRASIL-NETO JP, ALLAM N, RODRIGUES DE SOUZA E. A randomized, double-blind trial of repetitive transcranial magnetic stimulation in obsessive-compulsive disorder with three-month follow-up. *The Journal of neuropsychiatry and clinical neurosciences.* 2012;24(4):437-443.

62. MANSUR CG, MYCZKOWKI ML, DE BARROS CABRAL S et al. Placebo effect after prefrontal magnetic stimulation in the treatment of resistant obsessive-compulsive disorder: a randomized controlled trial. *Int J Neuropsychopharmacol.* 2011;14(10):1389-1397.

63. MANTOVANI A, SIMPSON HB, FALLON BA, ROSSI S, LISANBY SH. Randomized sham-controlled trial of repetitive transcranial magnetic stimulation in treatment-resistant obsessive-compulsive disorder. *Int J Neuropsychopharmacol.* 2010;13(2):217-227.

64. KANG JI, KIM CH, NAMKOONG K, LEE CI, KIM SJ. A randomized controlled study of sequentially applied repetitive transcranial magnetic stimulation in obsessive-compulsive disorder. *The Journal of clinical psychiatry.* 2009;70(12):1645-1651.

65. RUFFINI C, LOCATELLI M, LUCCA A, BENEDETTI F, INSACCO C, SMERALDI E. Augmentation effect of repetitive transcranial magnetic stimulation over the orbitofrontal cortex in drug-resistant obsessive-compulsive disorder patients: a controlled investigation. *Primary care companion to the Journal of clinical psychiatry.* 2009;11(5):226-230.

66. SACHDEV PS, LOO CK, MITCHELL PB, MCFARQUHAR TF, MALHI GS. Repetitive transcranial magnetic stimulation for the treatment of obsessive compulsive disorder: a double-blind controlled investigation. *Psychological medicine.* 2007;37(11):1645-1649.

67. PRASKO J, PASKOVA B, ZALESKY R et al. The effect of repetitive transcranial magnetic stimulation (rTMS) on symptoms in obsessive compulsive disorder. A randomized, double blind, sham controlled study. *Neuro endocrinology letters.* 2006;27(3):327-332.

68. ALONSO P, PUJOL J, CARDONER N et al. Right prefrontal repetitive transcranial magnetic stimulation in obsessive-compulsive disorder: a double-blind, placebo-controlled study. *The American journal of psychiatry.* 2001;158(7):1143-1145.

69. SACHDEV PS, MCBRIDE R, LOO CK, MITCHELL PB, MALHI GS, CROKER VM. Right versus left prefrontal transcranial magnetic stimulation for obsessive-compulsive disorder: a preliminary investigation. *The Journal of clinical psychiatry.* 2001;62(12):981-984.
